# Low glucose metabolite 3-phosphoglycerate switches PHGDH from serine synthesis to p53 activation to control cell fate

**DOI:** 10.1038/s41422-023-00874-4

**Published:** 2023-09-19

**Authors:** Yu-Qing Wu, Chen-Song Zhang, Jinye Xiong, Dong-Qi Cai, Chen-Zhe Wang, Yu Wang, Yan-Hui Liu, Yu Wang, Yiming Li, Jian Wu, Jianfeng Wu, Bin Lan, Xuefeng Wang, Siwei Chen, Xianglei Cao, Xiaoyan Wei, Hui-Hui Hu, Huiling Guo, Yaxin Yu, Abdul Ghafoor, Changchuan Xie, Yaying Wu, Zheni Xu, Cixiong Zhang, Mingxia Zhu, Xi Huang, Xiufeng Sun, Shu-Yong Lin, Hai-Long Piao, Jianyin Zhou, Sheng-Cai Lin

**Affiliations:** 1 State Key Laboratory of Cellular Stress Biology, Innovation Center for Cell Signaling Network, School of Life Sciences, Xiamen University, Xiamen, Fujian China; 2 Department of Hepatobiliary and Pancreatic Surgery, Zhongshan Hospital, Xiamen University, Xiamen, Fujian China; 3 Laboratory Animal Research Center, Xiamen University, Xiamen, Fujian China; 4 Fujian Provincial Key Laboratory of Tumor Biotherapy, Clinical Oncology School of Fujian Medical University, Fujian Cancer Hospital, Xiamen, Fujian China; 5 CAS Key Laboratory of Separation Science for Analytical Chemistry, Dalian Institute of Chemical Physics, Chinese Academy of Sciences, Dalian, Liaoning China

**Keywords:** Apoptosis, Nutrient signalling, Cancer

## Abstract

Glycolytic intermediary metabolites such as fructose-1,6-bisphosphate can serve as signals, controlling metabolic states beyond energy metabolism. However, whether glycolytic metabolites also play a role in controlling cell fate remains unexplored. Here, we find that low levels of glycolytic metabolite 3-phosphoglycerate (3-PGA) can switch phosphoglycerate dehydrogenase (PHGDH) from cataplerosis serine synthesis to pro-apoptotic activation of p53. PHGDH is a p53-binding protein, and when unoccupied by 3-PGA interacts with the scaffold protein AXIN in complex with the kinase HIPK2, both of which are also p53-binding proteins. This leads to the formation of a multivalent p53-binding complex that allows HIPK2 to specifically phosphorylate p53-Ser46 and thereby promote apoptosis. Furthermore, we show that PHGDH mutants (R135W and V261M) that are constitutively bound to 3-PGA abolish p53 activation even under low glucose conditions, while the mutants (T57A and T78A) unable to bind 3-PGA cause constitutive p53 activation and apoptosis in hepatocellular carcinoma (HCC) cells, even in the presence of high glucose. In vivo, PHGDH-T57A induces apoptosis and inhibits the growth of diethylnitrosamine-induced mouse HCC, whereas PHGDH-R135W prevents apoptosis and promotes HCC growth, and knockout of *Trp53* abolishes these effects above. Importantly, caloric restriction that lowers whole-body glucose levels can impede HCC growth dependent on PHGDH. Together, these results unveil a mechanism by which glucose availability autonomously controls p53 activity, providing a new paradigm of cell fate control by metabolic substrate availability.

## Introduction

The p53 protein is a master tumor suppressor, playing important roles in DNA repair, cell cycle arrest, cell death, and metabolic control.^1–4^ The stability and activity of p53 are tightly controlled through a series of posttranslational modifications such as phosphorylation and acetylation.^5,6^ Distinct patterns of phosphorylation for p53 activation, depending on the types of cells and stresses, have been identified, including phosphorylation of Ser15 and Ser20. In general, p-Ser15 and p-Ser20 are responsible for cell cycle arrest and DNA repair,^7–9^ while p-Ser46 is for proapoptotic functions.^10,11^ It was shown that phosphorylation at Ser15 and Ser20 stabilizes p53 and subsequently stimulates the expression of p21^WAF1/CIP1^,^12,13^ a CDK inhibitor; phosphorylation of Ser46 enables p53 to stimulate apoptosis through either promoting the expression of mitochondrial proapoptotic genes including PUMA, NOXA, BAX and p53AIP1,^11,14–16^ or enhancing the expression and activation of death receptor genes such as CD95/FAS.^17,18^ Phosphorylation of Ser46 also allows p53 to bind the anti-apoptotic proteins Bcl-xl, Bcl-2 and Mcl-1 to unleash BAX and BAK.^19–21^ As a result, p-p53-Ser46 promotes apoptosis by promoting BAX and BAK to form oligomers on the mitochondrial outer membrane to induce apoptosis.^19–21^ The stimulation of apoptosis by p-p53-Ser46 can be further augmented by p-Ser15 and p-Ser20, as the phosphorylation events elevate the protein levels of p53.^22,23^ Recent findings have also implicated p53 as a signaling node linking availabilities of glucose, glutamine or serine to the control of cell fate.^1,4^ It was shown that glucose limitation, often observed in the microenvironment of solid tumors, leads to activation and accumulation of p53.^1,24–27^ Naturally, AMPK, the AMP-activated protein kinase known to be activated in low glucose, has been investigated in p53 activation arising from glucose starvation.^28^ It was shown that, for example, AMPK phosphorylates the Ser15 residue of human p53 in HCT-116 cells^29^ and the Ser18 residue of mouse p53 in mouse embryonic fibroblasts (MEFs),^30^ to mediate cell cycle arrest under glucose starvation.^29,30^ Consistently, AICAR, an AMPK activator by acting as a mimetic of AMP,^31,32^ mimics the effects of low glucose on p53 activities.^29,30^ Caloric restriction (CR), which lowers whole-body glucose levels to activate AMPK, has also been demonstrated to elicit inhibitory effects on tumorigenesis,^33^ which was in part attributed to the p53-dependent cell cycle arrest.^26,34^ AMPK has also been reported to indirectly promote apoptosis in some other cell types and tissues. For example, it enhances the expression of p53 via transcriptional factors CREB and SMAR1,^35–37^ and stabilizes p53 via inhibiting ubiquitin ligases MDM2^38–40^ and MDMX^29^ in MEFs, HCT-116 cells and livers. However, there is no evidence supporting a direct role of AMPK in promoting the proapoptotic activity of p53. For one, AMPK does not phosphorylate p53-Ser46.^41,42^ We have previously shown that UV irradiation that causes DNA damage led to the formation of a supramolecular complex consisting of AXIN, p53, HIPK2, and TIP60. This complex allows phosphorylation of p53-Ser46 and initiates cell apoptosis in response to severe genotoxic stress.^43,44^ However, the innate physiological signal for p53 activation by the AXIN-scaffolded complex has remained elusive. Here, through systematic screening for glucose metabolite(s) and its sensor(s) that are required for mediating the proapoptotic p53 activation under low glucose conditions, we identify that it is phosphoglycerate dehydrogenase (PHGDH) known to be the enzyme converting the glycolytic intermediary product 3-phosphoglycerate (3-PGA) to 3-phosphohydroxypyruvate in serine synthesis, that interacts with AXIN to facilitate the formation of PHGDH–AXIN–HIPK2–TIP60–p53 complex, enabling the phosphorylation of p53-Ser46 to stimulate apoptosis of hepatocellular carcinoma (HCC) cells and suppress HCC growth in mice.

## Results

### Low glucose/3-PGA induces phosphorylation of p53 at Ser46

**Fig. 1:**
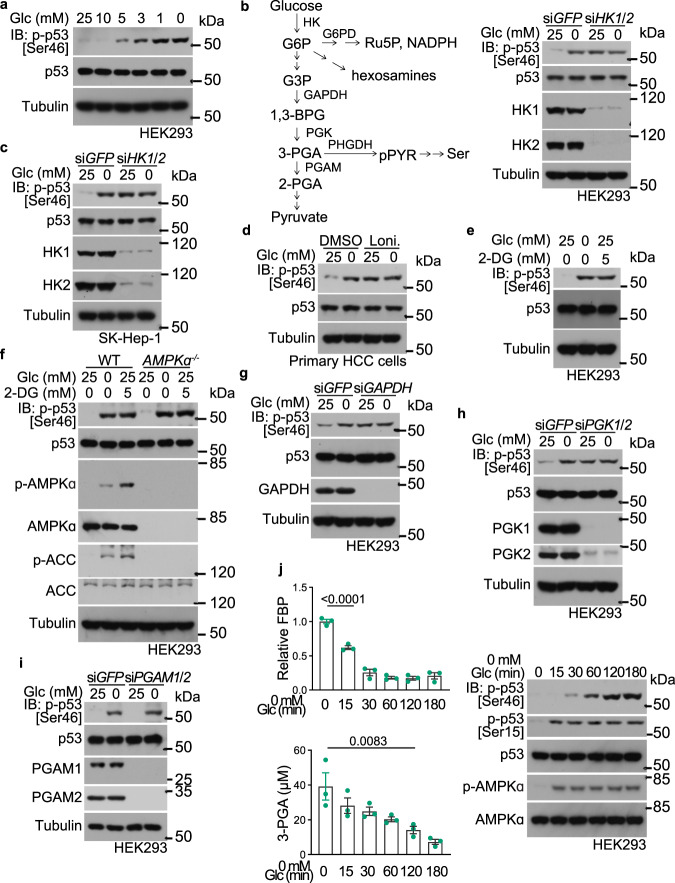
Low 3-PGA triggers phosphorylation of p53 at Ser46 (p-p53-Ser46). **a** Glucose, below 6 mM, induces p-Ser46 levels of p53. HEK293 cells were incubated in medium containing different concentrations of glucose (Glc) for 2 h, followed by determination of levels of p-p53-Ser46 by immunoblotting. **b**–**e** Knockdown or inhibition of hexokinases, which impairs glycolysis, increases p-p53-Ser46. HEK293 (**b,** **e**), SK-Hep-1 (**c**) cells with knockdown of both *HK1* and *HK2*, or human primary HCC cells (**d**), were glucose starved (**b,** **c**), or treated with 1 mM hexosekinase inhibitor lonidamine (Loni.; **d**), or with 5 mM 2-DG (**e**), all for 2 h, followed by immunoblotting to determine p-p53-Ser46. See also a schematic diagram of glycolysis and serine synthesis pathway on the left of **b**. **f** AMPK is not involved in p-p53-Ser46 induction in low glucose. HEK293 cells with knockout of both *AMPKα1* and *AMPKα2* were glucose starved or were treated with 5 mM 2-DG, for 2 h, followed by determination of p-p53-Ser46 by immunoblotting. **g**–**i** Lack of glycolytic intermediate 3-PGA is responsible for inducing p-p53-Ser46. HEK293 cells with knockdown of *GAPDH* (**g**), *PGK1* and *PGK2* (**h**), or *PGAM1* and *PGAM2* (**i**), were glucose starved for 2 h, followed by immunoblotting for p-p53-Ser46. **j** Inverse correlation between 3-PGA and the levels of p-p53-Ser46. HEK293 cells were glucose starved for the indicated time durations, followed by determination of levels of 3-PGA and other glycolytic intermediates including fructose-1,6-bisphosphate (FBP; left panel; data are means ± SEM, *n*  =  3, with *P* values calculated by one-way ANOVA, followed by Tukey). Levels of p-p53-Ser46 were determined by immunoblotting, with p-ACC and p-AMPKα serving as indicators of AMPK activity (right panel). Experiments were performed three times.

As shown in Fig. 1a, lowering glucose levels (< 5 mM) led to a specific increase of p53 phosphorylation at Ser46. We then started to search for glucose metabolite(s) that is involved in the regulation of p53. We first asked whether it is the lack of the glucose molecule per se or a downstream metabolite that is responsible for stimulating Ser46 phosphorylation. Knockdown of hexokinases (both *HK1* and *HK2*) resulted in a marked increase of p-Ser46 in HEK293 cells even in high glucose (Fig. 1b). Inhibition of the hexokinases by lonidamine showed a similar effect (Supplementary information, Fig. S1a). Promotion of p-p53-Ser46 by knockdown or inhibition of hexokinases was also observed in SK-Hep-1 HCC cell line and in primary HCC cells isolated from human patients (Fig. 1c, d). Addition of 2-deoxy-glucose (2-DG), a glucose analog that cannot proceed to the next step after hexokinases in glycolysis,^45,46^ also sufficiently promoted levels of p-Ser46 (Fig. 1e; Supplementary information, Fig. S1b, c), indicating that depletion of an intermediary metabolite(s) downstream of glucose controls the level of p-p53-Ser46. Consistent with the previous reports showing that AMPK is not involved in promoting p-Ser46,^41,42^ we observed similar levels of p-Ser46 in AMPKα-DKO (double knockout of *AMPKα1* and *AMPKα2*) HEK293 cells, either in low glucose or in the presence of 2-DG (Fig. 1f). As a control, the AMPK-mediated phosphorylation of Ser15^30^ was abolished in the AMPKα-DKO cells (Supplementary information, Fig. S1d). In addition, the two AMPK activators phenformin and AICAR^47,48^ failed to stimulate the level of p-Ser46, although p-Ser15 was stimulated in an AMPK-dependent manner (Supplementary information, Fig. S1d). We next knocked down glucose-6-phosphate dehydrogenase (*G6PD*), the rate-limiting enzyme in the pentose phosphate pathway, and found that the induction of p-Ser46 by low glucose was not affected (Supplementary information, Fig. S1e). The addition of N-acetyl-glucosamine, which maintains the hexosamine brunches in low glucose, also had no effect on p-Ser46 (Supplementary information, Fig. S1f). As p-Ser46 could still be stimulated by glucose deprivation in a medium containing pyruvate and all non-essential amino acid species (Fig. 1a), which sustained the intracellular pyruvate and amino acid levels during the glucose starvation (Supplementary information, Fig. S1g), it was concluded that the absence of an intermediary metabolite of glucose somewhere between glucose-6-phosphate and pyruvate may account for the stimulation of p53-pSer46. We next knocked down glyceraldehyde-3-phosphate dehydrogenase (*GAPDH*), which produces 1,3-bisphosphoglycerate (1,3-BPG) that can be further converted to 3-PGA, and observed elevation of p-Ser46 levels (Fig. 1g). We further found that knockdown of phosphoglycerate kinases (*PGK*; both *PGK1* and *PGK2*, converting 1,3-BPG to 3-PGA), but not phosphoglycerate mutases (*PGAM*; both *PGAM1* and *PGAM2*, converting 3-PGA to 2-phosphoglycerate), led to an increase of p-Ser46 (Fig. 1h, i). These results pointed to 3-PGA, the product of PGK, as the signaling metabolite responsible for modulating Ser46 phosphorylation. Consistently, a significant reduction of 3-PGA was observed after 2 h of glucose starvation, preceding the increase of p-p53-Ser46 (Fig. 1j). By contrast, fructose 1,6-bisphosphate was decreased much earlier, within 15 min of glucose starvation,^49^ correlating with the kinetics of AMPK activation and the increase of p-Ser15 promoted by AMPK (Fig. 1j; see also refs. ^30,49^).

### PHGDH is required for induction of Ser46 phosphorylation of p53 in low 3-PGA

**Fig. 2:**
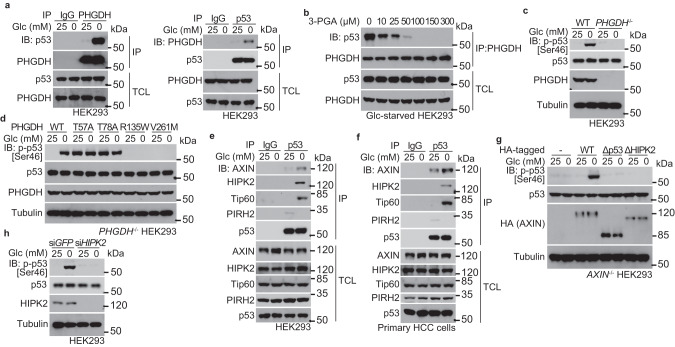
PHGDH transmits low 3-PGA signal to Ser46 phosphorylation of p53. **a** PHGDH interacts with p53. Cell lysates of HEK293 cells, regularly cultured or glucose starved for 2 h, were subjected to immunoprecipitation of endogenous PHGDH (left panel) or p53 (right panel), followed by immunoblotting of co-precipitated p53 (left panel) or PHGDH (right panel). TCL, total cell lysate. **b** Addition of 3-PGA to lysates of glucose-starved cells disrupts the PHGDH–p53 interaction. The 2-h-glucose-starved HEK293 cells were lysed, followed by addition of 3-PGA at indicated final concentrations into the lysates. Endogenous PHGDH was then immunoprecipitated, followed by immunoblotting with antibodies indicated. **c** PHGDH is required for p-p53-Ser46 induction in low glucose. HEK293 cells with knockout of *PHGDH* were glucose starved for 2 h, followed by immunoblotting to determine p-p53-Ser46. **d** Lack of occupancy of 3-PGA by PHGDH underlies the levels of p-p53-Ser46. *PHGDH*^–/–^ HEK293 cells were re-introduced with PHGDH-T57A and PHGDH-T78A that are defective in binding to 3-PGA, or PHGDH-R135W and PHGDH-V261M that are constitutively occupied with 3-PGA, and were glucose starved for 2 h, followed by immunoblotting to determine levels of p-p53-Ser46. **e**, **f** Glucose starvation induces the formation of PHGDH–AXIN–TIP60–HIPK2–p53 complex. HEK293 cells (**e**) or human primary HCC cells (**f**) were glucose starved for 2 h, followed by immunoprecipitation of endogenous p53 and immunoblotting with antibodies indicated. **g**, **h** AXIN tethers the upstream kinase HIPK2 for phosphorylation of p53-Ser46 in low glucose. HEK293 cells with knockdown of *HIPK2* (**h**), HEK293 cells with knockout of *AXIN* or with re-introduced AXIN truncation mutants defective in interacting with p53 (Δp53) or HIPK2 (ΔHIPK2) (**g**), were glucose starved for 2 h, followed by immunoblotting to determine p-p53-Ser46. Experiments were performed three times, except four times in **c** and **d**.

We next set out to identify the sensor(s) that senses the falling levels of the metabolite 3-PGA and signals to p53-Ser46 phosphorylation. We carried out mass spectrometry on protein complexes co-immunoprecipitated with p53 from HEK293 cells starved for glucose. Among the preys (listed in Supplementary information, Table S1), PHGDH was particularly intriguing, as it is the very enzyme that utilizes 3-PGA as a substrate to produce 3-phosphohydroxypyruvate for de novo serine synthesis. Immunoblotting confirmed that PHGDH interacted with p53, and such interaction was enhanced by glucose starvation (Fig. 2a). Re-addition of 3-PGA to the lysates from glucose-starved HEK293 cells dampened the PHGDH–p53 interaction (Fig. 2b). As a control, neither PGK1/2 nor PGAM1/2 interacted with p53 (Supplementary information, Fig. S1h). Moreover, p53-Ser46 failed to be phosphorylated in low glucose in *PHGDH*-knockout HEK293 cells (Fig. 2c). We then generated *PHGDH* mutants that either fail to bind 3-PGA (creating a pseudo-starvation signal in high glucose) or constitutively bind to 3-PGA (unable to proceed to the step of metabolizing 3-PGA to 3-phosphooxypyruvate, engendering a pseudo-satiety signal). It was reported that mutation of Thr57 or Thr78 to Ala (T57A or T78A) would blunt 3-PGA binding to PHGDH, because Thr57 is the residue required for 3-PGA binding (Protein Data Bank code 2g76), and the T78A mutant reduces the substrate-binding affinity (an ~10-fold higher *K*_m_, and a similar V_max_, compared with wild-type PHGDH) towards 3-PGA.^50^ The mutation of Arg135 to Trp (R135W) dampens the catalytic activity of PHGDH towards 3-PGA (by suppressing PHGDH binding and catalysis of NAD^+^ that is the co-substrate of PHGDH in conversion of 3-PGA to 3-phosphohydroxypyruvate) but retains a close-to-wild-type affinity towards 3-PGA,^51,52^ thereby rendering more 3-PGA-bound PHGDH in low glucose. Similar to the R135W mutant, we found that PHGDH-V261M displays a *K*_m_ for 3-PGA similar to that of wild-type PHGDH, but with a much lower V_max_ (Supplementary information, Fig. S2a). After re-introduction into the *PHGDH*-knockout HEK293 cells, both of the 3-PGA binding-defective mutants T78A and T57A elevated the levels of p-p53-Ser46 (Fig. 2d). In comparison, the constitutively 3-PGA-bound mutants R135W and V261M blocked the stimulation of p-p53-Ser46 by low glucose (Fig. 2d). Given that all of the four PHGDH mutants show significantly impaired catalytic activities,^50,52^ but exhibit distinct and even opposite roles in regulating p53, the results above strongly suggested that PHGDH is a direct sensor of 3-PGA, irrespective of catalytic activity, and transmits the signal of low glucose to Ser46 phosphorylation of p53.

### PHGDH forms a supramolecular complex with AXIN–HIPK2–p53

We next investigated the mechanism by which glucose starvation induces p53 phosphorylation at Ser46 in a PHGDH-dependent manner. It was known that p53-Ser46 phosphorylation is catalyzed by HIPK2 in response to UV irradiation,^53,54^ for which the scaffold protein AXIN serves as a platform.^43^ Remarkably, each of these proteins within the AXIN-based complex possesses one or more sites for p53 binding.^43,44^ It was also known that AXIN is occupied by the negative regulator PIRH2 in the absence of DNA damage; upon UV irradiation, the acetyltransferase TIP60 is recruited to the AXIN-based complex, and PIRH2 is replaced with HIPK2. This leads to the formation of the AXIN–TIP60–HIPK2–p53 complex, where HIPK2 phosphorylates Ser46 of and activates p53 to induce apoptosis.^43,44^ We next wondered whether AXIN may play a role in p53 regulation after sensing physiological changes of glucose. Indeed, the formation of the AXIN–TIP60–HIPK2–p53 complex was induced in HEK293 cells in low glucose (Fig. 2e). Similar results were obtained in primary HCC cells (Fig. 2f). Knockout of *AXIN*, or re-introduction of AXIN mutants lacking the regions for binding to p53 (AXIN^Δ209-338 & Δ678-753^, see ref. ^43^) or to HIPK2 (AXIN^Δ678-753^, see ref. ^43^) into the *AXIN*-knockout HEK293 cells failed to boost p-Ser46 of p53 in low glucose (Fig. 2g). Knockdown of *HIPK2* also impaired glucose starvation-induced p-Ser46 in HEK293 cells (Fig. 2h). These data suggested that the previously identified DNA damage-triggered p53-activating complex centered on AXIN enables a specialized pathway for mediating glucose starvation-induced p53 activation.

**Fig. 3:**
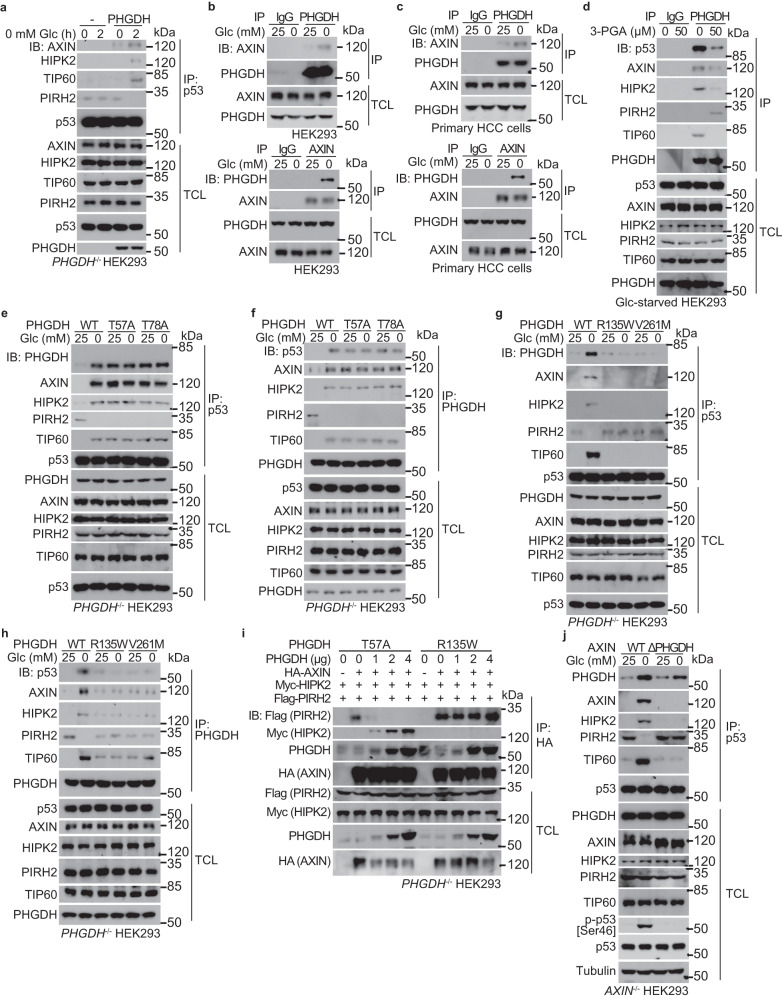
3-PGA-unoccupied PHGDH promotes the formation of PHGDH–AXIN–HIPK2–p53 complex. **a** PHGDH is required for the low glucose-induced complex formation with AXIN–HIPK2–p53 complex. *PHGDH*^–/–^ HEK293 cells were re-introduced with PHGDH or vector control, and were glucose starved for 2 h, followed by immunoprecipitation of endogenous p53 and immunoblotting with antibodies indicated. **b**, **c** Glucose starvation promotes the interaction between PHGDH and AXIN. HEK293 cells (**b**) or primary human HCC cells (**c**) were regularly cultured, or were glucose starved for 2 h, followed by immunoprecipitation of endogenous PHGDH (upper panel) or AXIN (lower panel) and immunoblotting for co-precipitated AXIN (upper panel) or PHGDH (lower panel). **d** 3-PGA dissociates the PHGDH–AXIN–HIPK2–p53 complex. The 2-h-glucose-starved HEK293 cells were lysed, followed by addition of 50 μM 3-PGA at indicated final concentrations into the lysates. Endogenous PHGDH was then immunoprecipitated, followed by immunoblotting. **e**–**h** Non-occupation of 3-PGA underlies the formation of PHGDH with AXIN–p53–HIPK2 complex. HEK293 cells with *PHGDH* knockout were re-introduced with PHGDH-T57A and PHGDH-T78A (**e,** **f**), or PHGDH-R135W and PHGDH-V261M (**g,** **h**), and were glucose starved for 2 h, followed by immunoprecipitation of endogenous p53 (**e,** **g**) or PHGDH (**f,** **h**). **i** The 3-PGA-unoccupied PHGDH dissociates PIRH2 from and recruits HIPK2 to AXIN. HEK293T cells were transfected with 2 μg of HA-tagged AXIN, 2 μg of FLAG-tagged PIRH2, 2 μg of FLAG-tagged HIPK2, along with FLAG-tagged PHGDH at indicated amounts. Cells were then lysed, followed by immunoprecipitation of HA-tag. **j** AXIN interaction with PHGDH is required for the formation of PHGDH–AXIN–HIPK2–p53 complex and phosphorylation of p53-Ser46 in low glucose. HEK293 cells with *AXIN* knockout were re-introduced with full-length AXIN or AXIN truncation mutant defective in binding to PHGDH (ΔPHGDH), and were glucose starved for 2 h, followed by analyses of proteins co-precipitated with p53, and of the levels of p-p53-Ser46. Experiments were performed three times.

We next asked how the PHGDH protein converges on the AXIN-based p53-activating complex. First of all, PHGDH is required for the low glucose-induced formation of AXIN-based p53-activating complex, as knockout of *PHGDH* blocked the formation of this complex in HEK293 cells (Fig. 3a). As a control, the formation of AXIN-based p53-activating complex could still be observed under UV irradiation in the *PHGDH*^–/–^ HEK293 cells (Supplementary information, Fig. S2b). Moreover, we found that the PHGDH interacted with endogenous or ectopically expressed AXIN in HEK293 cells and primary HCC cells, and the interaction was enhanced in low glucose (Fig. 3b, c; Supplementary information, Fig. S2c). Remarkably, co-immunoprecipitation assays showed that re-addition of 3-PGA to cell lysates dissociated PHGDH from the AXIN-based complex, with robust reductions of co-precipitated AXIN, HIPK2, p53 and TIP60 (Fig. 3d). In addition, PHGDH-T57A and -T78A mutants that fail to bind 3-PGA gave rise to similarly high affinities to AXIN regardless of the presence or absence of glucose (Fig. 3e); these pseudo-starvation mutants also excluded PIRH2 from binding to AXIN, and promoted the formation of AXIN–TIP60–HIPK2–p53 complex even in high glucose (Fig. 3e, f). In contrast, the constitutively-3-PGA-occupied mutants R135W and V261M of PHGDH were unable to interact with AXIN even in low glucose (Fig. 3g, h), and the AXIN complex retained PIRH2, forming the complex of AXIN–PIRH2–p53 instead of the AXIN–TIP60–HIPK2–p53 complex, even in low glucose (Fig. 3g, h). We also found that ectopically expressed PHGDH-T57A but not PHGDH-R135W dampened the interaction between AXIN and PIRH2 in high glucose, thereby increasing the association of AXIN with HIPK2 (Fig. 3i; Supplementary information, Fig. S2d). PHGDH-T57A also promoted the interaction between TIP60, HIPK2 and AXIN, and the interaction between p53 and AXIN in high glucose (Supplementary information, Fig. S2e, f). The results above suggested that PHGDH, when unoccupied by 3-PGA, promotes the formation of AXIN–p53–HIPK2–TIP60 complex. Domain mapping experiments revealed that the segment of amino acids 321–400 comprises the interface on AXIN for binding to PHGDH (Supplementary information, Fig. S2g), and deletion of this segment blocked the AXIN–PHGDH association and subsequent stimulation of p-Ser46, in low glucose (Fig. 3j).

### PHGDH-induced p-p53-Ser46 mediates low glucose-induced apoptosis and tumor suppression

**Fig. 4:**
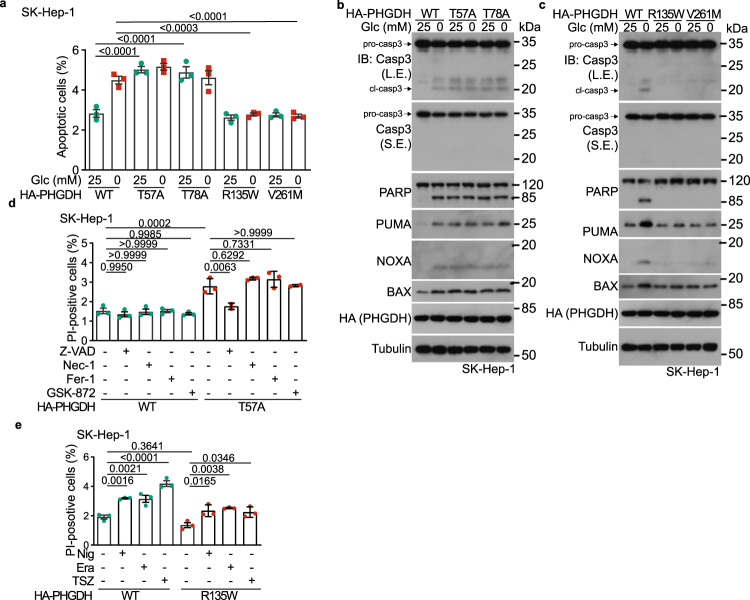
PHGDH mediates glucose starvation-induced apoptosis. **a**–**c** 3-PGA-unoccupied PHGDH induces apoptosis in SK-Hep-1 cells starved for glucose. Cells were infected with lentiviruses carrying HA-tagged PHGDH-T57A or PHGDH-T78A (**a,** **b**), or PHGDH-R135W or PHGDH-V261M (**a,** **c**) expressed under a doxycycline-inducible promoter. Cells were then incubated in RPMI 1640 medium or glucose-free RPMI 1640 medium, both containing doxycycline (100 ng/mL), for 8 h, followed by determining the levels of apoptotic cells via flow cytometry (**a**, see gating strategy for quantifying the populations of apoptotic cells in Supplementary information, Fig. S3a, and representative density plots in Supplementary information, Fig. S3b), and the levels of apoptotic markers by immunoblotting (**b,** **c**). Data in **a** are means ± SD, *n*  =  3, with *P* values calculated by two-way ANOVA, followed by Tukey. L.E., long exposure; S.E., short exposure. **d**, **e** PHGDH does not induce necroptosis, pyroptosis or ferroptosis in low glucose. SK-Hep-1 cells with inducible expression (as in **a**) of PHGDH-T57A (**d**) or PHGDH-R135W (**e**) were treated with 15 μM Necrostatin-1 (Nec-1; **d**), 0.75 μM GSK-872 (**d**), 0.5 μM Ferrostatin-1 (Fer-1; **d**), 20 μM Z-VAD (**d**), 10 nM Nigericin (Nig; **e**), 10 μM Erastin (Era; **e**), a combination of 10 IU/mL TNFα, 2.5 μM SM-164, and 10 μM Z-VAD (TSZ; **e**), all for 8 h, followed by determining the numbers of dead (PI-positive) cells via flow cytometry. Data are means ± SD, *n* = 3, with *P* values calculated by one-way ANOVA, followed by Tukey. See representative density plots in Supplementary information, Fig. S3h, i. Experiments were performed three times, except four times in **a**.

**Fig. 5:**
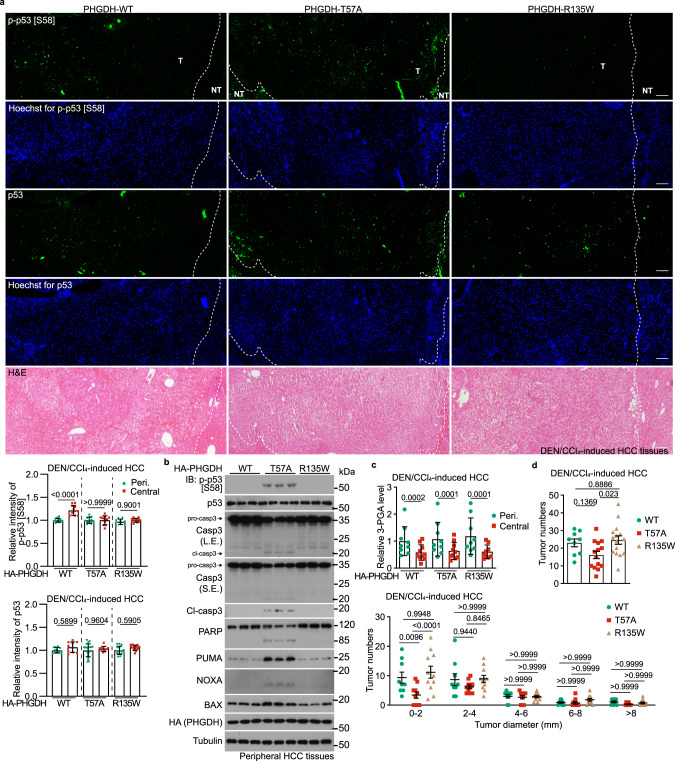
PHGDH mediates the inhibition of tumor growth in low glucose. **a**, **b** 3-PGA binding of PHGDH controls apoptosis in liver tissues. Hepatic PHGDH mutant-expressing mice (established by injecting AAVs carrying individual PHGDH mutants) were treated with DEN plus CCl_4_, as depicted in Supplementary information, Fig. S4a to induce HCC, and the livers were excised, followed by determining the p-Ser58-p53 by immunohistochemistry (IHC) (**a**; data are means ± SD, *n* = 10–13 fields from 7 mice, with *P* values calculated by one-way ANOVA, followed by Dunnet; Peri., peripheral) or by immunoblotting (**b**). See also apoptotic markers in HCC tissues in **b** (immunoblots) and Supplementary information, Fig. S5a, b (IHC images). T, tumor; NT, non-tumor. The scale bars are 100 μm. **c** Apoptosis levels are inversely correlated with levels of 3-PGA in the regions of liver tissues. Peripheral and central regions of HCC tissues were homogenized, followed by determining levels of glycolytic intermediates via CE-MS. Data are means ± SD, *n*  =  18–24, with *P* values calculated by two-way ANOVA, followed by Tukey. See levels of other glycolytic intermediates in Supplementary information, Fig. S5d. **d** Lack of 3-PGA binding of PHGDH inhibits tumor growth. Statistics of total tumor numbers of each mouse (upper panel; shown as means ± SD, *n*  =  9–15, with *P* values calculated by one-way ANOVA, followed by Tukey), as well as the numbers of tumor in each size/diameter: 0–2 mm, 2–4 mm, 4–6 mm, 6–8 mm, or > 8 mm (lower panel; shown as means ± SD, *n*  =  9–11, with *P* values calculated by two-way ANOVA, followed by Tukey) in each genotype, were shown. Experiments were performed three times.

**Fig. 6:**
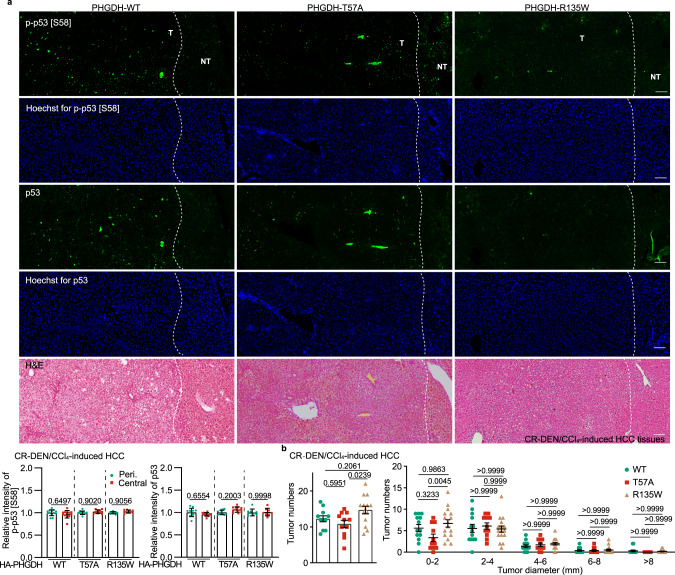
CR mimics the effects of PHGDH-T57A on HCC suppression. **a** CR induces p-Ser58-p53 and apoptosis to an extent similar to PHGDH-T57A in HCC tissues. Mice with hepatic expression of wild-type PHGDH and PHGDH mutants were induced to develop HCC as in Fig. 5a, except that these mice were also subjected to CR starting at 16 weeks old. The level of p-Ser58-p53 was then determined by IHC (data are means ± SD, *n* = 8–13 fields from 7 mice, with *P* values calculated by one-way ANOVA, followed by Tukey). See also apoptotic markers in HCC tissues in Supplementary information, Fig. S6a. The scale bars are 100 μm. **b** CR mimics the effects of PHGDH-T57A on HCC development. Statistics of tumor numbers were determined and are shown as in Fig. 5d. Data are means ± SEM, *n*  =  11–14 (left) or 9–11 (right), with *P* values calculated by one-way ANOVA (left) or two-way (right) ANOVA, followed by Tukey. Experiments were performed three times.

**Fig. 7:**
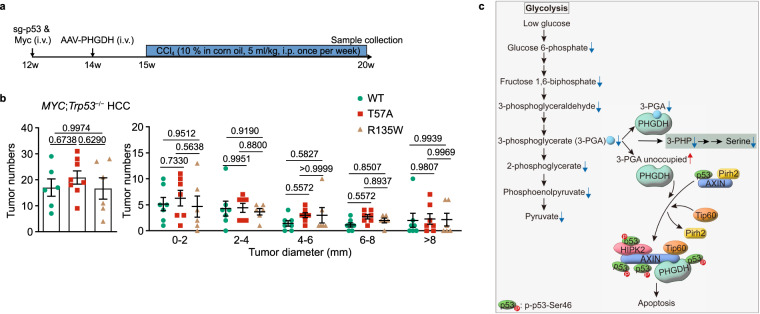
PHGDH modulates HCC growth through p53. **a**, **b** Liver-specific knockout of *Trp53* abolishes the effects of PHGDH on apoptosis and HCC development. The *MYC*;*Trp53*^−/−^ HCC mouse models with liver-specific expression of PHGDH mutants were established (**a**). Statistics of tumor numbers were determined and are shown in **b** (determined as in Fig. 5d; data are means ± SD, *n*  =  6–8 (left) or 6–7 (right), with *P* values calculated by one-way (left) or two-way (right) ANOVA, followed by Tukey). See also apoptotic markers of liver tissues from HCC mice in Supplementary information, Fig. S7a. **c** A schematic diagram depicting that PHGDH senses low 3-PGA to induce apoptosis in the control of cell fate. In low glucose, increased portion of PHGDH becomes unoccupied with 3-PGA, and displays a stronger affinity towards AXIN. As a result, TIP60 is recruited to AXIN, which helps dissociate PIRH2 from and promotes HIPK2 binding to AXIN. This leads to the formation of the AXIN–TIP60–HIPK2–p53 complex, where HIPK2 phosphorylates Ser46 of and activates p53 to induce apoptosis. Experiments were performed three times.

We next examined pathophysiological relevance of the low glucose-triggered assembly of the p53-activating complex orchestrated by PHGDH and AXIN. We found that, the re-introduction of the 3-PGA binding-defective (pseudo-starvation) PHGDH mutants T57A and T78A through the Tet-On system into *PHGDH*^–/–^ HEK293 cells caused apoptotic cell death regardless of glucose concentrations, as evidenced by the cleavage of caspase-3 and PARP, increased protein levels of PUMA, NOXA and BAX, and the positive staining of Annexin V (Supplementary information, Fig. S3c–e; see also gating strategy in Supplementary information, Fig. S3a). The pro-apoptotic roles of PHGDH-T57A and PHGDH-T78A, ectopically expressed through the Tet-On system, could also be observed in wild-type SK-Hep-1 cells, again indicative of dominant-positive roles of these mutants (Fig. 4a, b; Supplementary information, Fig. S3b). In comparison, expression of PHGDH-R135W and PHGDH-V261M mutants that constitutively bind to 3-PGA (pseudo-satiety) blocked the 8-h glucose starvation-induced apoptosis in both cell lines (Fig. 4a, c; Supplementary information, Fig. S3b, d, f). We also found that the re-introduction of the phosphorylation mimetic, Ser46 to Asp mutant (S46D) of p53 through the Tet-On system into *p53*^–/–^ HEK293 cells caused apoptotic cell death in high glucose, while the unphosphorylable Ser46 to Ala mutant (S46A) of p53 blocked the 8-h glucose starvation-induced apoptosis (Supplementary information, Fig. S3g). It is noteworthy that although phosphorylation of p53-Ser46 has also been shown to be related to necrotopsis,^55,56^ ferrotopsis^57^ and pyroptosis,^58^ the PHGDH-mediated cell death appears to be strictly apoptotic, because neither the necroptosis inhibitors Necrostatin-1^59^ and GSK-872^60^ nor the ferroptosis inhibitor Ferrostatin-1^61^ could dampen the cell death observed in SK-Hep-1 cells expressing PHGDH-T57A or PHGDH-T78A (Fig. 4d; Supplementary information, Fig. S3h). In addition, PHGDH-R135W and PHGDH-V261M failed to block the nigericin-induced pyroptosis, the erastin-induced ferroptosis, or the TNFα/SM-164/Z-VAD-induced necroptosis (Fig. 4e; Supplementary information, Fig. S3i). Of note, the PHGDH-p53-mediated apoptosis only occurred at early stage, i.e., 8-h of glucose starvation, as we found that prolonged, 16-h glucose starvation-induced apoptosis could not be blocked by PHGDH-R135W, PHGDH-V261M, or by p53-S46A (Supplementary information, Fig. S3j–m). Together, our findings indicate that after sensing low levels of the substrate 3-PGA, PHGDH induces conventional apoptosis through p53 activation. To test the role of PHGDH in animals, we utilized the mouse HCC model induced by diethylnitrosamine (DEN) plus CCl_4_ (ref. ^62^ and depicted in Supplementary information, Fig. S4a). We found that adeno-associated virus (AAV)-mediated ectopic expression of the PHGDH-T57A defective in binding to 3-PGA, significantly elevated the levels of p53-Ser58 (the mouse homolog of human p53-Ser46, see ref. ^63^) phosphorylation, in a dominant-positive manner, in both the tumor and non-tumor liver tissues from HCC mice (Fig. 5a, c; Supplementary information, Fig. S4c, see validation data of p53-Ser58 antibody in Supplementary information, Fig. S4b). The apoptosis markers were also increased in the PHGDH-T57A-expressing liver tissues, along with a decrease of the proliferative markers Ki67 and PCNA (Fig. 5b; Supplementary information, Figs. S4d–g, S5a–c). Of note, the dominant-positive role of 3-PGA binding-defective PHGDH-T57A in phosphorylation of p53-Ser58 was observed across the entire liver of mice without starvation (Supplementary information, Fig. S4c); inside the DEN-induced HCC liver, the increase of the p-Ser58 signal was most evident in the peripheral areas of HCC tumors but not in the most nutrient-stripped central areas of the tumor, where the levels of p-Ser58 were already constitutively high (Fig. 5a, b; see also refs. ^64,65^). Consistently, in the central areas of the HCC, levels of apoptosis were constitutively high irrespectively of the presence or absence of PHGDH mutants due to inherently low 3-PGA therein (Fig. 5c; Supplementary information, Fig. S5a–d). The inverse correlation between 3-PGA and p-Ser46 could also be observed in the HCC tissues from human patients (Supplementary information, Fig. S5e). Consistently, re-introduction of PHGDH-T57A decreased tumor numbers and volumes in the mouse HCC models (Fig. 5d). In comparison, the pseudo-satiety mutant PHGDH-R135W led to a significant inhibition of apoptosis, particularly in the central areas of the HCC tissues (Fig. 5a, b; Supplementary information, Figs. S4c–e, S5a, b). As a result, a significant promotion of tumor growth in the PHGDH-R135W-expressing mice was observed (Fig. 5d). The AAV-mediated ectopic expression of PHGDH-T57A and PHGDH-R135W in the liver of wild-type mice that retains normal serine synthesis did not change the levels of 3-PGA or other glycolytic intermediates (Fig. 5c; Supplementary information, Fig. S5d), emphasizing a causative role of availability of 3-PGA itself in cancer growth. As an additional control, the DEN-induced HCC mice expressing wild-type PHGDH or its mutants were subjected to CR. We found that under CR, mice expressing wild-type PHGDH showed decreased numbers and volumes of HCC tumors, in which increased levels of p-Ser58 and apoptosis were detected (Fig. 6a, b; Supplementary information, Fig. S6a–c). Interestingly, under CR that lowers whole-body glucose, the differences in tumor size and number between wild-type PHGDH and PHGDH-T57A groups were diminished, but the differences between wild-type PHGDH and PHGDH-R135W (Fig. 6b) were enlarged, reinforcing that glucose/3-PGA availability controls HCC development. We also determined the roles of PHGDH mutants in HCC development in a mouse model with liver-specific *Trp53* knockout and Myc expression (*MYC*;*Trp53*^−/−^ HCC,^66^ as depicted in Fig. 7a). In the absence of hepatic p53, the levels of apoptosis markers were constitutively low in both PHGDH-T57A- and PHGDH-R135W-expressing liver tissues (Supplementary information, Fig. S7a). Consistently, no differences in HCC tumor numbers or volumes between the livers expressing these two mutants could be observed (Fig. 7b). These results suggest that PHGDH modulates HCC growth through p53.

## Discussion

Our current findings have demonstrated that physiologically low glucose can autonomously initiate the assembly of the PHGDH–AXIN–HIPK2–TIP60–p53 complex, and that this complex formation is required for apoptosis (Fig. 7c). First of all, levels of p-Ser46 display an inverse correlation with glucose concentrations: low glucose, anywhere below 5 mM, can induce p-Ser46 levels of p53 even prior to the accumulation of the p53 protein levels. Second, the induction of p-Ser46 strictly depends on the presence of PHGDH, and mutations that fail to bind (T57A and T78A) or constitutively bind 3-PGA (R135W and V261M) increase or decrease p-Ser46, respectively. Moreover, although both of the two groups of PHGDH mutants exhibit lower catalytic activities than wild-type PHGDH (see refs. ^50,52^ and Supplementary information, Fig. S2a), only the 3-PGA binding-defective mutants T57A and T78A can induce Ser46 phosphorylation of p53 and the consequent apoptosis. We also found that HCC tissues expressing the 3-PGA binding-defective PHGDH mutants (via AAV) in the background of wild-type PHGDH expression that maintains serine synthesis showed a strong elevation of apoptosis even in peripheral areas with relatively high glucose, while the PHGDH mutants constitutively bound to 3-PGA prevented apoptosis in central areas, even under CR. Moreover, a strong correlation between hyperglycemia and hepatocarcinogenesis has been established in human patients,^67^ and dietary restriction significantly decreases the risk of incident HCC,^68^ in line with the findings that low PHGDH protein expression^69^ prevents apoptosis and hence drives metastatic dissemination during tumor metastasis in which the levels of 3-PGA are low.^70^ It thus stands to reason that it is the occupancy of the substrate 3-PGA that determines PHGDH to modulate p53 pro-apoptotic activity via controlling the phosphorylation of Ser46. Importantly, the regulation of cell fate by PHGDH is unrelated to the rates of serine synthesis, at least in HEK293 cells and in HCCs, consistent with the study showing that the enzymatic activity of PHGDH is dispensable for the development of HCC.^71^ Therefore, we have found a non-catalytic function of PHGDH, in tumor suppression, as opposed to the previously established tumor-promoting role of PHGDH, which depends on its catalytic function to upregulate serine synthesis (reviewed in refs. ^72,73^). It is possible that there are two opposing functions of PHGDH in regulating HCC development in different contests. While the non-catalytic function may clear cancer cells at early cancerous stages or within the established cancer tissues wherever glucose starvation occurs, e.g., in the central area of HCCs, the catalytic function, often coming from the PHGDH overexpression during HCC progression, promotes serine synthesis to support the growth of tumor when glucose supply is abundant. Interestingly, apart from the serine synthesis, PHGDH has been shown to promote tumor growth by acting as a transcription factor and a translational factor in the development of pancreatic cancer^74^ and HCC,^75^ respectively.

Evidently, normal cells, e.g., primary hepatocytes, are resistant to apoptosis in low glucose, unlike cancerous cells in HCC, according to data of our current study and from others,^26^ suggesting that the low glucose signaling has discriminative effects. This could be explained at least in part by the in general low expression of p53 in normal livers.^76^ During HCC development, the expression levels of p53 tend to increase,^77^ and in particular it may undergo multiple rounds of mutation.^78^ Whether the low glucose-triggered Ser46-specific phosphorylation may have functions in addition to induction of apoptosis, such as causation of cell cycle arrest, induction of immune response and hepatic fibrosis, or regulation of stemness of hepatic progenitor cells and HCC stem cells during HCC formation (as does the p53 protein; reviewed in refs. ^3,79^), awaits future studies. In sum, we have demonstrated that the depletion of 3-PGA, under physiological conditions such as intermittent starvation, can constitute a signal to control cell fate, providing a new perspective on the pleiotropic effects of glycolytic intermediates on metabolic homeostasis and health.

## Materials and methods

### Antibodies

Rabbit anti-phospho-p53-Ser58 (1:1000 dilution for immunoblotting (IB) and 1:1000 for immunohistochemistry (IHC); for detection of p-Ser58 in mouse tissues) was generated as described previously,^63^ except that the peptide GSH-QDVEEFFEGPS(P) was used. Rabbit anti-AXIN (cat# 2074, RRID: AB_2062419; 1:1000 for IB and 1:1000 for immunoprecipitation (IP)), anti-phospho-AMPKα-Thr172 (cat# 2535, RRID: AB_331250; 1:1000 for IB), anti-AMPKα (cat# 2532, RRID: AB_330331; 1:1000 for IB), anti-phospho-ACC-Ser79 (cat# 3661, RRID: AB_330337; 1:1000 for IB), anti-ACC (cat# 3662, RRID: AB_2219400; 1:1000 for IB), anti-phospho-p53-Ser46 (cat# 2521, RRID: AB_10828689; 1:500 for IB and 1:100 for IHC; for detection of p-Ser46 in human cells and tissues), anti-phospho-p53-Ser15 (cat# 9284, RRID: AB_331464; 1:1000 for IB), anti-PARP (cat# 9532, RRID: AB_659884; 1:1000 for IB), anti-BAX (cat# 2772, RRID: AB_10695870; 1:1000 for IB), anti-PUMA (cat#  98672; 1:1000 for IB), anti-PCNA (cat# 13110, RRID: AB_2636979; 1:1000 for IHC), anti-cleaved caspase-3 (cl-casp3; cat# 9661, RRID: AB_2341188; 1:1000 for IB and 1:400 for IHC), anti-caspase-3 (casp3; cat# 14220, RRID: AB_2798429; 1:1000 for IB), anti-HK1 (cat# 2024, RRID: AB_2116996; 1:1000 for IB), anti-HK2 (cat# 2867, RRID: AB_2232946; 1:1000 for IB), anti-PGK1 (cat# 63536; 1:1000 for IB), anti-G6PD (cat# 12263, RRID: AB_2797861; 1:1000 for IB), anti-GAPDH (cat#  5174, RRID: AB_10622025; 1:1000 for IB), anti-HA-tag (cat# 3724, RRID: AB_1549585; 1:1000 for IB), and mouse anti-Myc-tag (cat# 2276, RRID: AB_331783; 1:1000 for IB and 1:100 for IP), and HRP-conjugated mouse anti-rabbit IgG (conformation-specific, cat# 5127, RRID: AB_10892860; 1:2000 for IB) and rabbit anti-mouse IgG (light chain-specific, cat# 58802, RRID: AB_2799549; 1:2000 for IB) antibodies were purchased from Cell Signaling Technology. Rabbit anti-PHGDH (cat# HPA021241, RRID: AB_1855299; 1:1000 for IB) and rabbit anti-FLAG (cat# F7425, RRID: AB_439687; 1:4000 for IB) antibodies were purchased from Sigma. Rabbit anti-p53 (cat# sc-6243, RRID: AB_653753; 1:1000 for IB), mouse anti-p53 (cat# sc-126, RRID: AB_628082; 1:50 for IP, 1:1000 for IB and 1:100 for IHC), anti-PHGDH (cat# sc-100317, RRID: AB_2165393; 1:50 for IP), anti-HA (cat# sc-7392, RRID: AB_2894930; 1:100 for IP), and normal mouse control IgG (cat# sc-2025, RRID: AB_737182; 1:100 for IP) antibodies were purchased from Santa Cruz Biotechnology. Rabbit anti-Pirh2 (cat# ab189907; 1:1000 for IB) and anti-P53-AIP1 (cat# ab3678, RRID: AB_303996; 1:1000 for IHC) antibodies were purchased from Abcam. Rabbit anti-TIP60 (cat# 10827-1-AP, RRID: AB_2128431; 1:1000 for IB), anti-PGK2 (cat# 13686-1, RRID: AB_2161237; 1:1000 for IB), anti-PGAM1 (cat# 16126-1, RRID: AB_2160786; 1:1000 for IB), anti-PGAM2 (cat# 15550-1, RRID: AB_2299336; 1:1000 for IB), anti-HIPK2 (cat# 55408-1, RRID: AB_2881323; 1:1000 for IB), and mouse anti-tubulin (cat# 66031-1-Ig, RRID: AB_11042766; 1:20,000 for IB) antibodies were purchased from Proteintech. Rabbit anti-Ki67 (cat# MA5-14520, RRID: AB_10979488; 1:200 for IHC) and Alexa Fluor™ 488-conjugated, goat anti-mouse IgG (cat# R37120, RRID: AB_255654; 1:300 for IHC) and goat anti-rabbit IgG (cat# A-11034, RRID: AB_2576217; 1:300 for IHC) antibodies were purchased from Thermo. Mouse anti-NOXA (cat# OP180, RRID: AB_2268468; 1:1000 for IB) antibody was purchased from Calbiochem. Normal rabbit control IgG (cat# CR1; 1:100 for IP) was purchased from Sino Biological. HRP-conjugated, goat anti-mouse IgG (cat# 115-035-003, RRID: AB_10015289; 1:5000 for IB) and goat anti-rabbit IgG (cat# 111-035-003, RRID: AB_2313567; 1:5000 for IB) antibodies were purchased from Jackson ImmunoResearch.

### Chemicals and assay kits

DMSO (cat# D2650), glucose (cat# G7021), CsCl (cat# 289329), NaHCO_3_ (cat# S5761), 3-PGA (cat# P8877), N-acetylglucosamine (GlcNAc; cat# A3286), Trizma^®^ base (Tris; cat# T1503), NaCl (cat# S7653), EDTA (cat# E6758), EGTA (cat# E3889), SDS (cat# 436143), formaldehyde solution (formalin; cat# F8775), sodium pyrophosphate (cat# P8135), β-glycerophosphate (cat# 50020), 2-DG (cat# D8375), phenformin (cat# P7045), AICAR (cat# A9978), tamoxifen (cat# T5648), phosphate-buffered saline (PBS; cat# P5493), IPTG (cat# I6758), Na_2_HPO_4_ (cat# S7907), NaH_2_PO_4_ (cat# S8282), glycerol (cat# G5516), imidazole (cat# I5513), Triton^TM^ X-100 (cat# T9284), L-glutathione reduced (GSH; cat# G4251), hydrazine (cat# 309400), glutamine (cat# 49419), NAD^+^ (cat# N3014), NADH (cat# N6005), Tween-20 (cat# P9416), polybrene (cat# H9268), lonidamine (cat# L4900), N-nitrosodiethylamine (DEN; cat# N0756), Necrostatin-1 (cat# N9037), GSK-872 (cat# 5303890001), Ferrostatin-1 (cat# SML0583), Erastin (cat# E7781), BSA (cat# A2153), Nonfat-Dried Milk bovine (cat# M7409), methoxyamine hydrochloride (cat# 89803), MTBSTFA (with 1% t-BDMCS; cat# M-108), hexane (cat# 34859), pyridine (cat# 270970), myristic-d27 acid (cat# 68698), bisBenzimide H 33342 trihydrochloride (Hoechst; cat# B2261), HIS-Select Nickel Affinity Gel (cat# P6611), EZview™ Red Anti-c-Myc Affinity Gel (cat# E6654) and corn oil (cat# C8267) were purchased from Sigma. rProtein A Sepharose Fast Flow (cat# 17127904) and Protein G Sepharose 4 Fast Flow (cat# 17061806) beads, and Superdex 200 Increase 10/300 GL (cat# 28990944) column were purchased from Cytiva. Nigericin (cat# HY-127019), SM-164 (cat# HY-15989) and Z-VAD (cat# HY-16658) were purchased from MedChemExpress. Doxycycline (cat# S4163) was purchased from Selleckchem. TNFα (cat# Z01001) was purchased from GenScript. Polyethylenimine (PEI; cat# 23966) was purchased from Polysciences. WesternBright^TM^ ECL and Peroxide solutions (cat# 210414-73) were purchased from Advansta. Protease inhibitor cocktail (cat# 70221) was purchased from Roche. ProLong™ Diamond Antifade Mountant (cat# P36970), Lipofectamine^TM^ 2000 (cat# 11668500), DMEM, high glucose (cat# 12800082), DMEM, no glucose (cat# 11966025), RPMI 1640 medium (cat# 11875093), RPMI 1640, no glucose (cat# 11879020), MEM non-essential amino acids solution (cat# 11140050), fetal bovine serum (cat# 10099141 C), penicillin-streptomycin (cat# 15140163), GlutaMAX^TM^ (cat# 35050061), sodium pyruvate (cat# 11360070), 0.25% trypsin (cat# 15050057) and Prestained Protein MW Marker (cat# 26612) were purchased from Thermo. CCl_4_ was purchased from Macklin. Apo-BrdU In Situ DNA Fragmentation Assay Kit (cat# K401) was purchased from BioVision. Annexin V-FITC/PI Apoptosis Detection Kit (cat# 40302ES20) was purchased from Yeasen. [U-13C]-3-PGA (cat# P358008) was purchased from Toronto Research Chemicals.

### Animals

All mouse experiments were approved by the Institutional Animal Care and the Animal Committee of Xiamen University (XMULAC20180028 and XMULAC20220050).

Wild-type C57BL/6 mice (000664) and *Trp53*-floxed mice (008462, established as described previously,^80^ and kindly provided by Dr. Anton Berns from University of Amsterdam) were obtained from The Jackson Laboratory. The *Trp53*-floxed mice were then crossed with *Alb*-*CreERT2* mice to generate inducible liver-specific *Trp53* knockout mice. Hepatic *Trp53* was deleted by injecting intraperitoneally the mice with tamoxifen (dissolved in corn oil) at 200 mg/kg, 3 times a week. Mice with PHGDH, p53 and their mutant expression were generated by injection of AAVs carrying PHGDH, p53, or their mutants via the tail vein. Levels of the PHGDH or p53 proteins were analyzed at 4 weeks after the virus injection.

Mice were housed with free access to water and standard diet (65% carbohydrate, 11% fat, 24% protein) under specific pathogen-free conditions. The light was on from 8 a.m. to 8 p.m., with temperature kept at 21–24 °C, and humidity at 40%–70%. For CR, mice were individually caged for 1 month for precondition; each mouse was fed with 2.5 g of standard diet (70% of ad libitum food intake for a mouse at 3 months old and above) at 5 p.m. at each day. Male littermate controls were used throughout the study.

### Human primary HCC cells

Human HCC cells were isolated from surgically removed liver tissues. Fresh tissues were minced, followed by digesting in 0.25% (w/v) trypsin supplemented with 0.5 mg/mL collagenase type IV for 10 min at 37 °C. Cells were then immediately plated (at 90% confluence) in collagen-coated 6-well plates in DMEM plus 10% FBS, 100 IU penicillin and 100 mg/mL streptomycin. After 6 h of attachment, the medium was refreshed, and cells were cultured for another 12 h before further use. This study was approved by the Human Research Ethics Committee of the Zhongshan Hospital, affiliated to Xiamen University (XMZSYYKY 2022-072), following the principles of the Declaration of Helsinki. Written informed consent was obtained from all participants.

### Mouse HCC induction

The DEN/CCl_4_-induced mouse HCC model was established as described previously,^62^ with minor modifications. Briefly, mice at 4 weeks old and 5 weeks old were intraperitoneally injected with 80 mg/mL DEN twice, followed by intraperitoneally injected with 10% (v/v in corn oil) CCl_4_ at a dose of 5 mL/kg every week from 8 weeks old. AAVs carrying PHGDH and its mutants were intravenously injected every 8 weeks from 16 weeks. Liver samples were collected at 40 weeks old. CR was started at 12 weeks of age.

The *MYC*;*Trp53*^−/−^ mouse HCC model was established as described previously,^66^ with minor modifications. Briefly, mice of 12 weeks old were used, and a mixture containing 30 μg of pX330-sg-p53,^81^ 30 μg of PT3-EF1a-Myc, and 7.5 μg (4:1 ratio) of pCMV/SB10 transposase-encoding plasmids dissolved in 2 mL of 0.9% NaCl solution was prepared before the hydrodynamic tail-vein injection. For each mouse, a total volume of mixture corresponding to 10% of body weight was injected into the lateral tail vein in 5–7 s. Mice were then intraperitoneally injected with 10% (v/v in corn oil) CCl_4_ at a dose of 5 mL/kg every week beginning at 1 week after hydrodynamic tail-vein injection. AAVs carrying PHGDH and its mutants were intravenously injected once at 2 weeks after hydrodynamic tail-vein injection. Liver samples were collected at 8 weeks after the hydrodynamic tail-vein injection.

### Histology

For hematoxylin & eosin (H&E) staining, liver tissues excised from blood-drained mice were cut into 0.5-cm^3^ cubes, and were fixed in 4% (v/v) paraformaldehyde for 24 h at room temperature, then transferred to embedding cassettes. The cassettes were then washed in running water for 12 h, followed by successive soaking each for 1 h in 70% ethanol (v/v in water), 80% ethanol, and 95% ethanol. The fixed tissues were further dehydrated in anhydrous ethanol for 1 h twice, followed by immersing in 50% xylene (v/v in ethanol) for 30 min, and two changes of xylene, 15 min each. Tissues were then immersed in 50% paraffin wax (58–60 °C; dissolved in equal volume of xylene) for 1 h, followed by two changes of paraffin wax, 1 h each. The dehydrated tissues were embedded in paraffin on a HistoCore Arcadia Paraffin Embedding Machine (Leica). Paraffin blocks were sectioned at a thickness of 5 μm, spread at 42 °C in a water bath for 3 min (HI1210, Leica), dried on an adhesion microscope slide at 42 °C on a heated flattening table (HI1220, Leica) for 12 h, and then incubated at 45 °C in a hot-air dryer for another 4 h. The sections were then de-paraffinized at 70 °C in a hot-air dryer for 4 h, followed by re-hydrating in the following order: two changes of xylene at 70 °C, 10 min each, two changes of anhydrous ethanol 5 min each, two changes of 95% ethanol 5 min each, one change each for 5 min of 80% ethanol, 70% ethanol, and 50% ethanol and then briefly in water. The sections were then stained in hematoxylin solution for 8 min, then washed in running water for 5 min, differentiated in 1% hydrochloric acid (in ethanol) for 30 s, washed in running water for 1 min, and immersed in 0.2% (v/v in water) ammonium hydroxide solution for 30 s, washed in running water for 1 min, and stained in eosin Y solution for 30 s. The stained sections were dehydrated in 70% ethanol for 5 min, twice in 95% ethanol 5 min each, twice in anhydrous ethanol 5 min each, two changes of xylene 15 min each. The stained sections were mounted with Canada balsam and visualized on an Axioscan 7 (Zeiss).

For IHC staining of phospho-p53 and apoptotic markers, liver tissues were fixed, dehydrated, embedded, sectioned and re-hydrated as in H&E staining, and were washed with water for three times, 5 min each at room temperature. The sections were then incubated in pre-heated (~95 °C) citrate antigen retrieval buffer (1 mM sodium citrate, pH 6.0, 0.05% (v/v) Tween-20) for 1.5 min, followed by cooling at room temperature for another 30 min. The sections were then washed with washing buffer (0.1% (v/v) Tween-20 in PBS) twice, 5 min each at room temperature, and then incubated in 10% (w/w) H_2_O_2_ (in methanol) solution at room temperature for 5 min, followed by washing with washing buffer for three times, 5 min each at room temperature. The sections were then incubated in 1% (w/v) BSA (diluted with PBS), at room temperature for 20 min. After draining, the liver sections were circled by a PAP pen (cat# Z377821, Sigma), followed by incubation with primary antibodies (diluted in 1% BSA solution) for 16 h at 4 °C in a dark, humidified chamber, followed by washing with washing buffer for 3 times, 5 min each at room temperature. The sections were then incubated with Alexa Fluor 488 goat anti-rabbit IgG or goat anti-mouse IgG (diluted in 1% BSA solution) for 30 min at room temperature in a dark, humidified chamber, followed by washing with washing buffer for 3 times, 5 min each at room temperature. After draining, sections were incubated with 0.4 μg/mL Hoechst solution for 15 min at room temperature, and were mounted with Canada balsam and visualized on an Axioscan 7 (Zeiss), except Supplementary information, Fig. S4b, c, e–g on a DM4 B (Leica).

The TUNEL/BrdU staining was performed as described previously,^82^ with minor modifications. Briefly, the liver tissues were fixed, dehydrated, embedded, sectioned and re-hydrated as in H&E staining, and were stained using the Apo-BrdU In Situ DNA Fragmentation Assay Kit according to the manufacturer’s instructions. Briefly, the re-hydrated sections were incubated in 0.85% for 5 min, PBS for 5 min, and 4% (v/v) formaldehyde for 15 min, all at room temperature. The sections were rinsed with PBS twice, 5 min each at room temperature, followed by incubating in 20 μg/mL of Proteinase K Solution (prepared by mixing 2 μL of 10 mg/mL Protease K with 998 μL of 100 mM Tris-HCl, pH 8.0 and 50 mM EDTA to generate a 1-mL solution) and rinsing with PBS, 5 min each at room temperature. The sections were then fixed with 4% (v/v) formaldehyde for 5 min at room temperature, followed by rinsing with PBS for 5 min at room temperature. The sections were washed with Wash Buffer (included in the Apo-BrdU In Situ DNA Fragmentation Assay Kit) twice, 5 min each at room temperature, and then incubated with DNA Labeling Solution (by mixing 10 μL of TdT Reaction Buffer, 0.75 μL of TdT Enzyme, 8 μL of Br-dUTP with 32.25 μL of double distilled water to generate a 51 μL solution) for 1 h at 37 °C in a dark, humidified chamber. The sections were then washed with PBS twice, 5 min each at room temperature, followed by incubating with Antibody Solution (by mixing 5 μL of anti-BrdU-FITC antibody and 95 μL of Rinse Buffer) for 0.5 h at room temperature in a dark, humidified chamber. The sections were then incubated with Propidium Iodide (PI)/RNase A solution for 0.5 h at room temperature in a dark, humidified chamber, and then washed twice with double distilled water, 5 min each at room temperature. Sections were mounted with ProLong Diamond Antifade Mountant, and were visualized on an Axioscan 7, except Supplementary Information, Fig. S4d on a DM4 B.

Histochemical images using DM4 B were processed by LAS X software (v.3.0.2.16120, Leica), and those on Axioscan 7 by Zen Blue 3.4 software (Zeiss). Images were formatted by Photoshop 2022 software (Adobe).

### Plasmid

Full-length cDNAs used in this study were obtained either by PCR using cDNA from MEFs or by purchasing from Origene, Sino Biological or Genescript. Mutations of PHGDH and AXIN were performed by PCR-based site-directed mutagenesis using PrimeSTAR HS polymerase (Takara). pX330-sg-p53 (#59910, Addgene) plasmid was a kind gift from Dr. Tyler Jacks, PT3-EF1a-Myc (#92046, Addgene) from Dr. Xin Chen, pCMV/SB10 (#24551, Addgene) from Dr. Perry Hackett, and pAAV2/9n (#112865, Addgene) from Dr. James M. Wilson. Expression plasmids for various epitope-tagged proteins were constructed in the pcDNA3.3 vector (#K830001, Thermo) for transfection (ectopic expression), in the pBOBI vector for lentivirus packaging (stable expression), in the pLVX-IRES (#631849, Takara) for doxycycline-inducible expression, or in the pET-28a (#69864-3, Novagen) for bacterial expression. All expression plasmids constructed in this study have been deposited to Addgene (https://www.addgene.org/Sheng-cai_Lin/). The lentivirus-based vector pLV-H1-EF1a-puro (#SORT-B19, Biosettia) was used for the expression of siRNA in HEK293 cells and SK-Hep-1 cells, and the AAV-based vector pAAV2 for mouse liver. The control (si*GFP*) siRNA was constructed as described previously.^83^ All constructs were verified by sequencing (Invitrogen, China). The sequences for each siRNA are as follows: 5’-GGCGGTGAAACAGGATTTA-3’ for human *HIPK2*, 5’-GCTGTCCCAAGCATCAAAT-3’ for human *PGK1*, 5’-GGTGGTGGAATGGCTTATA-3’ for human *PGK2*, 5’-GGTCTCAATAAAGCAGAAA-3’ for human *PGAM1*, and 5’-CCCTACTACAACTCCATTA-3’ for human *PGAM2*. The siRNAs against human *HK1*, *HK2*, *G6PD* and *GAPDH* were constructed and validated as described previously.^49,84^ *Escherichia coli* strain DH5α (cat# PTA-1977) was purchased from ATCC and Stbl3 (cat# C737303) from Thermo. All plasmids were amplified in *E*. *coli* strain DH5α, except those mutagenesis in Stbl3. All plasmids were verified by sequencing and were purified using the CsCl density gradient ultracentrifugation method.

### Cell culture

In this study, no cell line used is on the list of known misidentified cell lines maintained by the International Cell Line Authentication Committee (https://iclac.org/databases/cross-contaminations/). HEK293T cells (cat# CRL-3216) and HEK293 cells (cat# CRL-1573) were purchased from ATCC, and SK-Hep-1 cells (cat# CL-0212) from Procell Life Science&Technology Co., Ltd. All cell lines were verified to be free of mycoplasma contamination and authenticated by STR sequencing.

HEK293T cells and HEK293 cells were maintained in DMEM (high glucose) supplemented with 3.7 g/L NaHCO_3_, 10% FBS, 100 IU penicillin, 100 mg/mL streptomycin at 37 °C in a humidified incubator containing 5% CO_2_. PEI at a final concentration of 10 μM was used to transfect HEK293T cells. The total DNA to be transfected for each plate was adjusted to the same amount by using the relevant empty vector. Transfected cells were harvested at 24 h after transfection. SK-Hep-1 cells were maintained in RPMI 1640 medium supplemented with MEM non-essential amino acids, sodium pyruvate, GlutaMAX^TM^, 10% FBS, 100 IU penicillin, and 100 mg/mL streptomycin at 37 °C in a humidified incubator containing 5% CO_2_.

Lentiviruses, including those for knockdown or stable expression, were packaged in HEK293T cells by transfection using Lipofectamine 2000. At 30 h post transfection, medium (DMEM supplemented with MEM non-essential amino acids; ~2 mL) was collected and centrifuged at 5000× *g* for 3 min at room temperature. The supernatant was then mixed with 10 μg/mL (final concentration; for HEK293) and 20 μg/mL polybrene (final concentration; for SK-Hep-1), followed by centrifuging at 3000× *g* for 30 min at room temperature (spinfection). Cells were incubated for another 12 h before further treatments.

The genes (*PRKAA1*, *PRKAA2*, *PHGDH*, *p53* and *AXIN*) were deleted from HEK293 cells using the CRISPR-Cas9 system. Nucleotides were annealed to their complements containing the cloning tag aaac, and inserted into the back-to-back BsmB I restriction sites of lentiCRISPRv2 vector. The sequence for each sgRNA is as follows: 5’-GAAGATCGGCCACTACATTC-3’ for *PRKAA1*, 5’-GGCGGCTCTTTCAGCAGATT-3’ for *PRKAA2*, 5’-GAAGGGGAAATCTCTCACGG-3’ for *PHGDH*, 5’-TCTCGAAGCGCTCACGCCCA-3’ for *p53*, and 5’-GGGGTTGACTGGCTCCCGCC-3’ for *AXIN*. The constructs were then subjected to lentivirus packaging by transfecting 2 µg of relevant DNA using Lipofectamine 2000 transfection reagent into 10^5^ HEK293T cells cultured in a well of a 6-well plate. At 30 h post transfection, the virus (~2 mL) was collected for infecting HEK293 cells at 40% confluence for 36 h. When cells were approaching to confluence, they were single-cell sorted into 96-well dishes. Clones were expanded and evaluated for knockout status by sequencing.

For glucose starvation, cells were rinsed twice with PBS, and then incubated in glucose-free DMEM or RPMI 1640 supplemented with 10% FBS and 1 mM sodium pyruvate for desired periods of time at 37 °C. For UV irradiation, cells were exposed to UV at a dose of 75 J/m^2^ on a UV crosslinker (CL-508; UVITEC) after removal of culture medium (DMEM) and plate lid. The irradiated cells were then cultured in DMEM for another 16 h before harvest.

### Packaging and injection of AAV

AAVs were packaged in HEK293T cells using the protocol from Grieger et al. ^85^ In brief, cells used for in-house viral production were maintained in 150-mm dishes. A total of 7 μg of pAAV-RC2/9 (AAV2 inverted terminal repeat (ITR) vectors pseudo-typed with AAV9 capsid) plasmid, 21 μg of pAAV-helper plasmid and 7 μg of pAAV2 plasmid (carrying PHGDH or its mutant) were added to 4 mL of DMEM without phenol red, followed by mixing with 175 μL of PEI solution (1 mg/mL, pH 7.5). The mixtures were then incubated at room temperature for 20 min and then added to the dishes. At 60 h after transfection, cells were collected by scraping and centrifugation. The viral particles were purified from the pellet using an Optiprep gradient as previously described.^85^ The titers of purified AAV were determined by real-time qPCR as previously described.^86^ Viruses were stored at –80 °C before use, and were delivered to mice intravenously by lateral tail-vein injection. For each mouse, 1 × 10^11^ particles of virus, adjusted to 200 μL of final volume (with PBS, pH 7.4) was injected.

### Immunoprecipitation and immunoblotting

For immunoprecipitation (IP) of endogenous p53, PHGDH and AXIN, a 10-cm dish of HEK293 cells, SK-Hep-1 cells (grown to 95% confluence), or HCC cells from 0.5 g of patient liver tissue were collected. Cells were lysed with 400 μL/dish of ice-cold lysis buffer (20 mM Tris-HCl, pH 7.5, 150 mM NaCl, 1 mM EDTA, 1 mM EGTA, 1% (v/w) Triton X-100, 2.5 mM sodium pyrophosphate, 1 mM β-glycerophosphate, with protease inhibitor cocktail), followed by sonication and centrifugation at 4 °C for 15 min. Cell lysates were incubated with respective antibodies overnight. Overnight protein aggregates were pre-cleared by centrifugation at 20,000× *g* for 10 min, and protein A/G beads (by mixing rProtein A Sepharose Fast Flow beads and Protein G Sepharose 4 Fast Flow beads in the 1:1 proportion, and then balanced with 100 times volume of lysis buffer twice) were then added into the lysate/antibody mixture (to 1% (v/v) final concentration) for another 1 h at 4 °C. The beads were centrifuged and washed with 100 times volume of lysis buffer for 3 times (by centrifuging at 2000× *g*) at 4 °C and then mixed with an equal volume of 2× SDS sample buffer and boiled for 10 min before immunoblotting.

For IP of ectopically expressed AXIN or PHGDH, HEK293T cells of a 6 cm-dish were used. Cells were transfected with different expression plasmids, and were collected at 24 h posttransfection. For IP of stably expressed AXIN or PHGDH, HEK293 cells of a 10 cm-dish were collected. Cells were lysed in 500 µL of ice-cold lysis buffer, followed by sonication and centrifugation at 4 °C for 15 min. Anti-HA (1:100) or anti-Myc (1:100) antibodies, along with protein A/G beads (1:100), were added into the supernatant and mixed for 4 h at 4 °C. The beads were washed with 200 times volume of lysis buffer for 3 times at 4 °C and then mixed with an equal volume of 2× SDS sample buffer and boiled for 10 min before immunoblotting.

To analyze the levels of p-p53, p-AMPKα and p-ACC in HEK293 cells, SK-Hep-1 cells and primary HCC cells, these cells were grown to 95% confluence in a well of a 6-well dish (except for HCC cells from 0.5 g of patient liver tissues) and were lysed with 250 μL of ice-cold lysis buffer. To analyze the levels of p-p53 and apoptotic markers in mouse livers, 100 mg of freshly excised tissue was lysed with ice-cold lysis buffer (10 μL/mg liver weight), followed by homogenization by a hand-hold homogenizer T 10 basic ULTRA-TURRAX^®^ equipped with an S 10 N - 5 G dispersing tool, IKA. The lysates were sonicated and then centrifuged at 20,000× *g* for 10 min at 4 °C. After discarding the pellet, an equal volume of 2× SDS sample buffer was added into the supernatant. Samples were then boiled for 10 min before gel electrophoresis and immunoblotting.

All protein samples were subjected to immunoblotting on the same day of preparation without freeze-thaw cycle.

For immunoblotting, the SDS-polyacrylamide gels were prepared as described previously.^86^ Samples of less than 10 μL (except caspase-3, 20 μL) were loaded into wells, and the electrophoresis was run at 100 V by a Mini-PROTEAN Tetra Electrophoresis Cell (BIO-RAD). The thickness of gels used in this study was 1.0 mm, except for caspase-3, 1.5 mm. All samples were resolved on 8% resolving gels, except PGK1, PGK2, PGAM1, PGAM2, GAPDH and PIRH2 on 10%, caspase-3 on 13%, and PUMA, NOXA and BAX 15%. The resolved proteins were then transferred to the PVDF membrane (0.45 μm, cat# IPVH00010, Merck) as described previously.^86^ The blotted PVDF membrane was then blocked by 5% (w/v) BSA (for all antibodies against phosphorylated proteins) or 5% (w/v) non-fat milk (for all antibodies against total proteins) dissolved in TBST (40 mM Tris, 275 μM NaCl, 0.2% (v/v) Tween-20, pH 7.6) for 2 h on an orbital shaker at 60 rpm at room temperature, followed by rinsing with TBST for twice, 5 min each. The PVDF membrane was incubated with desired primary antibody overnight at 4 °C on an orbital shaker at 60 rpm, followed by rinsing with TBST for three times, 5 min each at room temperature, and then the secondary antibodies for 3 h at room temperature with gentle shaking. The secondary antibody was then removed, and the PVDF membrane was further washed with TBST for 3 times, 5 min each at room temperature. PVDF membranes were incubated in ECL mixture (by mixing equal volumes of ECL solution and Peroxide solution for 5 min), then life with Medical X-Ray Film (FUJIFILM). The films were then developed with X-OMAT MX Developer and Replenisher and X-OMAT MX Fixer and Replenisher solutions (Carestream) on a Medical X-Ray Processor (Carestream) using Developer (Model 002, Carestream). The developed films were scanned using a Perfection V850 Pro scanner (Epson) with an Epson Scan software (v.3.9.3.4), and were cropped using Photoshop 2022 software (Adobe). Levels of total proteins and phosphorylated proteins were analyzed on separate gels, and representative immunoblots are shown.

### Determination of apoptosis by flow cytometry

Effects of low glucose on apoptosis were determined by a flow cytometry-based method using the Annexin V-FITC/PI Apoptosis Detection Kit. Briefly, HEK293 cells and SK-Hep-1 cells grown to 70%–80% confluence in a 6-well dish were washed with 3 mL of PBS (pre-heated to 37 °C) for 3 times, and then trypsinized with 0.25% trypsin, followed by centrifugation at 300× *g* for 5 min at 4 °C. Some 1 × 10^5^ of trypsinized cells were washed with 0.5 mL of ice-cold PBS twice (by centrifuging at 300× *g* for 5 min at 4 °C). Cell suspensions were centrifuged at 300× *g* for 5 min at 4 °C, and then resuspended with 100 μL of Binding Buffer, followed by staining with 5 μL of Annexin V-FITC solution and 10 μL of PI solution for another 15 min at room temperature in the dark. Cell suspensions were then diluted with 400 μL of Binding Buffer, and then immediately subjected to flow cytometry analysis. Flow cytometry was performed on a Cytoflex LX (Beckman Coulter), with the 488-nm (50 mW) laser and the 525/40 filter used to excite and detect the fluorescence of Annexin V-FITC, and the 561-nm (30 mW) laser and 585/42 filter PI. Detector voltages were optimized using a modified voltage titration approach.^87^ Gating strategies used during the analysis were: (a) the FSC-A and SSC-A for selecting intact cells, and (b) FSC-H and FSC-width for excluding doublets (shown in Supplementary information, Fig. S3a). Gate boundaries were set either based on control samples, or followed density distributions based on best practices. Data were collected by the FACSDiva software (v8.0.2, BD Biosciences), followed by exporting as the FCS 3.1 format. The numbers of apoptotic cells (sum of Q2 and Q3, as indicated in each panel) were quantified with the FlowJo software (v10.4.0, BD Biosciences). During the analysis, a combination of manual gating and computational analysis approaches^88^ was used.

### Measurement of glycolytic intermediates

Levels of glycolytic intermediates were analyzed by capillary electrophoresis-based mass spectrometry (CE-MS), and each measurement required cells collected from one 10-cm dish (60%–70% confluence) or 100 mg of HCC tissue. Cells were washed with 25 mL of 5% (m/v) mannitol solution (dissolved in water), and were instantly frozen in liquid nitrogen. After thawing, cells were then lysed with 1 mL of methanol containing IS1 (internal standard 1; 50 µM L-methionine sulfone, 50 µM D-campher-10-sulfonic acid, dissolved in water; 1:500 (v/v) added to the methanol and used to standardize the metabolite intensity and to adjust the migration time). For analysis of metabolites in the liver, the tissue was freshly excised by freeze-clamping, then washed in pre-cooled 5% mannitol solution and ground in 1 mL of methanol containing IS1. The lysate was then mixed with 1 mL of chloroform and 400 μL of water, followed by 20 s of vortexing. After centrifugation at 15,000× *g* for 15 min at 4 °C, 450 μL of aqueous phase was collected and was then filtrated through a 5 kDa cutoff filter (cat# OD003C34, PALL) by centrifuging at 12,000× *g* for 3 h at 4 °C. In parallel, quality control samples were prepared by combining 10 μL of the aqueous phase from each sample and then filtered alongside the samples. The filtered aqueous phase was then freeze-dried in a vacuum concentrator (a LABCONCO #7310037 centrifuge connected to a LABCONCO #7460037 cold trap and an EDWARDS nXDS15i pump) at 4 °C for 12 h, and then dissolved in 100 μL of water containing IS2 (50 µM 3-aminopyrrolidine dihydrochloride, 50 µM N,N-diethyl-2-phenylacetamide, 50 µM trimesic acid, 50 µM 2-naphtol-3,6-disulfonic acid disodium salt, dissolved in methanol; used to adjust the migration time). A total of 20 μL of re-dissolved solution was then loaded into an injection vial (cat# 9301-0978, Agilent Technologies; equipped with a snap cap (cat# 5042-6491, Agilent Technologies)). Before CE-MS analysis, the fused-silica capillary (cat# TSP050375, i.d. 50 µm × 80 cm; Polymicro Technologies) was installed in a CE/MS cassette (cat# G1603A, Agilent Technologies) on the CE system (Agilent Technologies 7100). The capillary was then pre-conditioned with Conditioning Buffer (25 mM ammonium acetate, 75 mM diammonium hydrogen phosphate, pH 8.5) for 30 min, followed by balancing with Running Buffer (50 mM ammonium acetate, pH 8.5; freshly prepared) for another 1 h. CE-MS analysis was run at anion mode, during which the capillary was washed by Conditioning Buffer, followed by injection of the samples at a pressure of 50 mbar for 25 s, and then separation with a constant voltage at –30 kV for another 40 min. Sheath Liquid (0.1 μM hexakis(1H, 1H, 3H-tetrafluoropropoxy)phosphazine, 10 μM ammonium trifluoroacetate, dissolved in methanol/water (50% v/v); freshly prepared) was flowed at 1 mL/min through a 1:100 flow splitter (Agilent Technologies 1260 Infinity II; actual flow rate to the MS: 10 μL/min) throughout each run. The parameters of mass spectrometer (Agilent Technologies 6545) were set as: (a) ion source: Dual AJS ESI; (b) polarity: negative; (c) nozzle voltage: 2000 V; (d) fragmentor voltage: 110 V; (e) skimmer voltage: 50 V; (f) OCT RFV: 500 V; (g) drying gas (N_2_) flow rate: 7 L/min; (h) drying gas (N_2_) temperature: 300 °C; (i) nebulizer gas pressure: 8 psig; (j) sheath gas temperature: 125 °C; (k) sheath gas (N_2_) flow rate: 4 L/min; (l) capillary voltage (applied onto the sprayer): 3500 V; (m) reference (lock) masses: m/z 1,033.988109 for hexakis(1H, 1H, 3H-tetrafluoropropoxy)phosphazine, and m/z 112.985587 for trifluoroacetic acid; (n) scanning range: 50–1100 m/z; and (n) scanning rate: 1.5 spectra/s. Data were collected using MassHunter LC/MS acquisition 10.1.48 (Agilent), and were processed using Qualitative Analysis B.06.00 (Agilent). For quantification of 3-PGA, [U-13C]-3-PGA was used to generate standard curves by plotting the ratios of detected labeled 3-PGA (areas) to the areas of IS1 and IS3, against the added concentrations of labeled 3-PGA. The amount of 3-PGA was then estimated according to equations generated from standard curves. The average cell volume was determined to be 2000 μm^3^ as described previously,^89^ and cell density 1.1 g/mL according to ref. ^90^

### Measurement of intracellular amino acids

Levels of pyruvate and amino acids were determined by gas chromatography-mass spectrometry (GC-MS) as described previously,^91^ with minor modifications. Briefly, HEK293 cells from one 10-cm dish (60%–70% confluence) were collected for each measurement. Cells were incubated with DMEM (no glucose) supplemented with 1 mM sodium pyruvate, 10% FBS and 25 mM glucose or not (for starvation) for 2 h, and then lysed with 1 mL of 80% methanol (v/v in water) containing 10 µg/mL myristic-d27 acid as an internal standard, followed with 20 s of vortexing. After centrifugation at 15,000× *g* for 15 min at 4 °C, 800 µL of each supernatant (aqueous phase) was freeze-dried at 4 °C for 24 h. The lyophilized samples were then subjected to derivatization by vortexing for 1 min after mixing each with 50 µL of freshly prepared methoxyamine hydrochloride (20 mg/mL in pyridine), followed by incubation at 4 °C for 1 h. The mixtures were sonicated at 0 °C by bathing in ice slurry for 10 min, and were then incubated at 37 °C for 1.5 h, followed by mixing with 50 µL of MTBSTFA and incubated at 55 °C for 1 h. Before subjecting to GC-MS, samples were centrifuged at 15,000× *g* for 10 min, and some 60 μL of each supernatant was loaded into an injection vial (cat# 5182-0714, Agilent; with an insert (cat. HM-1270, Zhejiang Hamag Technology)) equipped with a snap cap (cat# HM-0722, Zhejiang Hamag Technology). GC was performed on an HP-5MS column (30 m × 0.25 mm i.d., 0.25 μm film thickness; cat# 19091S-433; Agilent) using a GC/MSD instrument (7890-5977B, Agilent). Briefly, the injector temperature of GC/MSD was set at 260 °C. The column oven temperature was first held at 70 °C for 2 min, then increased to 180 °C at the rate of 7 °C/min, then to 250 °C at 5 °C/min, then to 310 °C at 25 °C/min, where it was held for 15 min. The MSD transfer temperature was 280 °C. The MS quadrupole and source temperature were maintained at 150 °C and 230 °C, respectively. Measurements were performed in both a scan mode and a selected ion monitoring mode. The following m/z values were used for each compound: 174 for pyruvate; 260 for L-alanine; 418 for L-aspartic acid; 432 for L-glutamic acid; 246 for L-glycine; 258 for L-proline; 390 for L-serine; 200 for L-leucine and L-lsoleucine; 302 for L-phenylalanine; 302 for L-tyrosine; 244 for L-tryptophan; 302 for L-threonine; 406 for L-cysteine; 320 for L-methionine; 417 for L-asparagine; 431 for L-glutamine; 300 for L-lysine; and 440 for L-histidine. Data were collected using the MassHunter GC/MS Acquisition software (v.B.07.04.2260, Agilent). For quantification, peaks were extracted and integrated using GC-MS MassHunter Workstation Qualitative Analysis software (v.B.07.01SP1, Agilent).

### Protein expression

The pET-28a-PHGDH, pET-28a-PHGDH-V261M and pET-28a-PSAT1 plasmids were transformed into the *E*. *coli* strain BL21 (DE3) (cat# EC0114, Thermo), followed by culturing in LB medium in a shaker at 200 rpm at 37 °C. The cultures of transformed cells were induced with 0.1 mM IPTG at an OD_600_ of 1.0. After incubating for another 12 h at 160 rpm at 16 °C, the cells were collected, followed by homogenization in a His binding buffer (50 mM sodium phosphate, pH 7.4, 150 mM NaCl, 1% Triton X-100, 5% glycerol, and 10 mM imidazole) on ice. The homogenates were then sonicated on ice, and were subjected to centrifugation at 150,000× *g* for 30 min at 4 °C, followed by incubating with Nickel Affinity Gel (pre-balanced with His binding buffer; 1:100) on a rotator for 2 h at 4 °C. The Nickel Affinity Gel was then washed with 100 times the volume of ice-cold His wash buffer (50 mM sodium phosphate, pH 7.4, 150 mM NaCl, and 20 mM imidazole), followed by incubating with His elution buffer (50 mM sodium phosphate, pH 7.4, 150 mM NaCl, and 250 mM imidazole) at 4 °C. Proteins were concentrated to ~3 mg/mL by ultrafiltration (UFC905096, Millipore) at 4 °C, then subjected to gel filtration (Superdex 200) balanced with a buffer containing 50 mM Tris-HCl, pH 7.4 and 150 mM NaCl at 4 °C.

### Determination of PHGDH enzymatic activity

The enzymatic activity of PHGDH was determined as described previously,^50^ with minor modifications. Briefly, 10 μg of PHGDH and 5 µg of PSAT1 were pre-incubated in 100 μL of Reaction buffer (333 mM Tris-HCl, pH 9.0 and 1.7 mM EDTA, 3.3 mM GSH, 333 mM hydrazine, 10 mM glutamine and 2 mM NAD^+^) at 37 °C for 5 min in a well of a glass-bottom, 96-well microplate (cat# 3635, Corning). The reaction was initiated by pipetting 200 μL of 3-PGA solution (at desired concentrations; dissolved in Reaction Buffer) pre-warmed at 37 °C, into the well, followed by mixing and recording OD_340_ at 30-s intervals on a SpectraMax M5 microplate reader (Molecular Devices). The PHGDH activities were assessed with the initial velocities of NADH formation calculated according to the standard curve by plotting the values of OD_340_ against the added concentrations of NADH. All measurements were carried out in triplicate. Data were collected using the SoftMax Pro software (v.5.4.1.1, Molecular Devices) and exported to Graphpad Prism software (v.9.5.1, Graphpad) for further analysis.

### Identification of p53-binding proteins

Twenty 10-cm dishes of HEK293 cells glucose-starved for 2 h were lysed with 10 mL of ice-cold lysis buffer, followed by sonication. After centrifugation, supernatants were incubated with 200 μL of mouse anti-p53 antibody or mouse control IgG antibody at 4 °C for 4 h. Protein aggregates were cleared by centrifugation at 20,000× *g* for 15 min, and 200 μL of protein A/G beads (pre-balanced with 100 times volume of lysis buffer twice) were then added into the lysate/antibody mixture for another 1 h at 4 °C. The beads were centrifuged and washed with 100 times volume of lysis buffer for 3 times (by centrifuging at 2000× *g*) at 4 °C and then mixed with a 1/5 volume of 5× SDS sample buffer and boiled for 10 min, followed by SDS-PAGE. The gels were stained with Coomassie Brilliant Blue R-250 dye, followed by decoloring. Gels were then excised, and were subjected to in-gel trypsin digestion and dried. Samples were analyzed on a nanoElute (Bruker) coupled to a timsTOF Pro (Bruker) equipped with a CaptiveSpray source. Peptides were dissolved in 5 μL of 0.1% formic acid (v/v) and were loaded onto a homemade C18 column (35 cm × 75 μm, ID of 1.9 μm, 100 Å). Samples were then eluted for 60 min with linear gradients of 3%–35% acetonitrile (v/v, in 0.1% formic acid) at a flow rate of 0.3 μL/min. MS data were acquired with a timsTOF Pro mass spectrometer (Bruker) operated in PASEF mode, and were analyzed using Peaks Studio software (X^+^, Bioinformatics Solutions). The mouse UniProt Reference Proteome database was used during data analysis.

### Statistical analysis

Statistical analyzes were performed using Prism 9 (GraphPad Software). Each group of data was subjected to Kolmogorov-Smirnov test, Anderson–Darling test, D’Agostino–Pearson omnibus test or Shapiro–Wilk test for normal distribution when applicable. An unpaired two-tailed Student’s *t*-test was used to determine significance between two groups of normally distributed data. Welch’s correction was used for groups with unequal variances. An unpaired two-tailed Mann–Whitney test was used to determine significance between data without a normal distribution. For comparisons between multiple groups, an ordinary one-way or two-way ANOVA was used, followed by Tukey, Sidak, Dunnett or Dunn as specified in the figure legends. The assumptions of homogeneity of error variances were tested using F-test (*P* >  0.05). For comparison between multiple groups with two fixed factors, an ordinary two-way ANOVA was used, followed by Tukey’s or Sidak’s multiple comparisons test as specified in the figure legends. Geisser–Greenhouse’s correction was used where applicable. The adjusted means and SEM, or SD, were recorded when the analysis met the above standards. Differences were considered significant when *P* < 0.05, or *P* > 0.05 with large differences of observed effects (as suggested in refs. ^92,93^).

### Supplementary information

Fig. S1

Fig. S2

Fig. S3

Fig. S4

Fig. S5

Fig. S6

Fig. S7

Fig. S8

Supplementary information Table S1

### Source data

Source data for all graphs

## Data Availability

The MS proteomics data have been deposited to the ProteomeXchange Consortium (http://proteomecentral.proteomexchange.org) through the iProX partner repository^94,95^ with the dataset identifier PXD041361. The data supporting the findings of this study are available within the paper and Supplementary information. Uncropped immunoblots and the source data for all graphs are uploaded. The analysis was performed using standard protocols with previously described analysis tools.
