# Supplementary material for: Low glucose metabolite 3-phosphoglycerate switches PHGDH from serine synthesis to p53 activation to control cell fate
Source: Cell Res. 2023 Sep 19;33(11):835–50. doi: 10.1038/s41422-023-00874-4 (PMC10624847; doi:10.1038/s41422-023-00874-4)
Supplement: Supplementary file 1 — Fig. S1 [file 41422_2023_874_MOESM1_ESM.pdf]

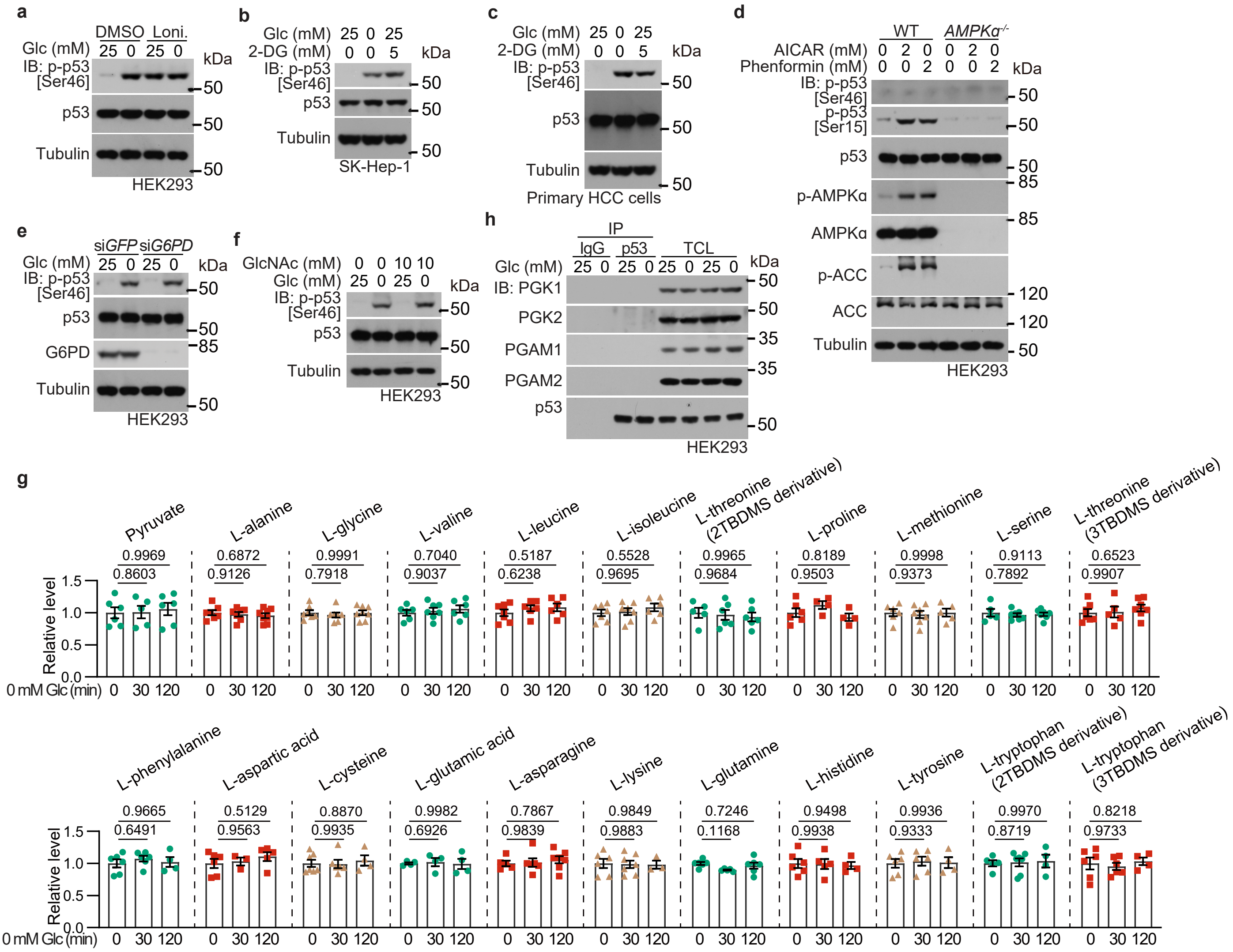

Fig. S1 Determination of glycolytic intermediate(s) that inversely correlates with phosphorylation of p53-Ser46. a-c Inhibition of hexokinases triggers p-p53-Ser46. HEK293 (**a**), SK-Hep-1 (**b**) or human primary HCC (**c**) cells were treated with 1 mM lonidamine (**a**), 5 mM 2-DG (**b**, **c**), or glucose starved (**a-c**), all for 2 h, followed by immunoblotting for p-p53-Ser46. **d** AMPK does not phosphorylate p53-Ser46. HEK293 cells with knockout of both *AMPKα1* and *AMPKα2* were treated with 2 mM AICAR or phenformin for 2 h, followed by immunoblotting for p-p53-Ser46. **e**, **f** Pentose phosphate pathway and hexosamine shunts of glycolysis are not involved in the phosphorylation of p53-Ser46. HEK293 cells (**f**) or HEK293 cells with *G6PD* knockdown (**e**) were glucose starved, or treated with 10 mM of N-acetyl-glucosamine (GlcNAc; **f**), both for 2 h, followed by immunoblotting for p-p53-Ser46. **g** Glucose starvation does not change intracellular amino acids and pyruvate levels. HEK293 cells were glucose starved for 2 h, followed by determination of levels of amino acids and pyruvate by GC-MS; data are means ± SEM, n = 3-8, with p values calculated by one-way ANOVA, followed by Tukey). **h** PGK and PGAM do not interact with p53. HEK293 cells were glucose starved for 2 h, followed by immunoprecipitation using anti-p53 antibody and immunoblotting of PGK1, PGK2, PGAM1 and PGAM2. Experiments in this figure were performed three times.
