# Supplementary material for: Low glucose metabolite 3-phosphoglycerate switches PHGDH from serine synthesis to p53 activation to control cell fate
Source: Cell Res. 2023 Sep 19;33(11):835–50. doi: 10.1038/s41422-023-00874-4 (PMC10624847; doi:10.1038/s41422-023-00874-4)
Supplement: Supplementary file 3 — Fig. S3 [file 41422_2023_874_MOESM3_ESM.pdf]

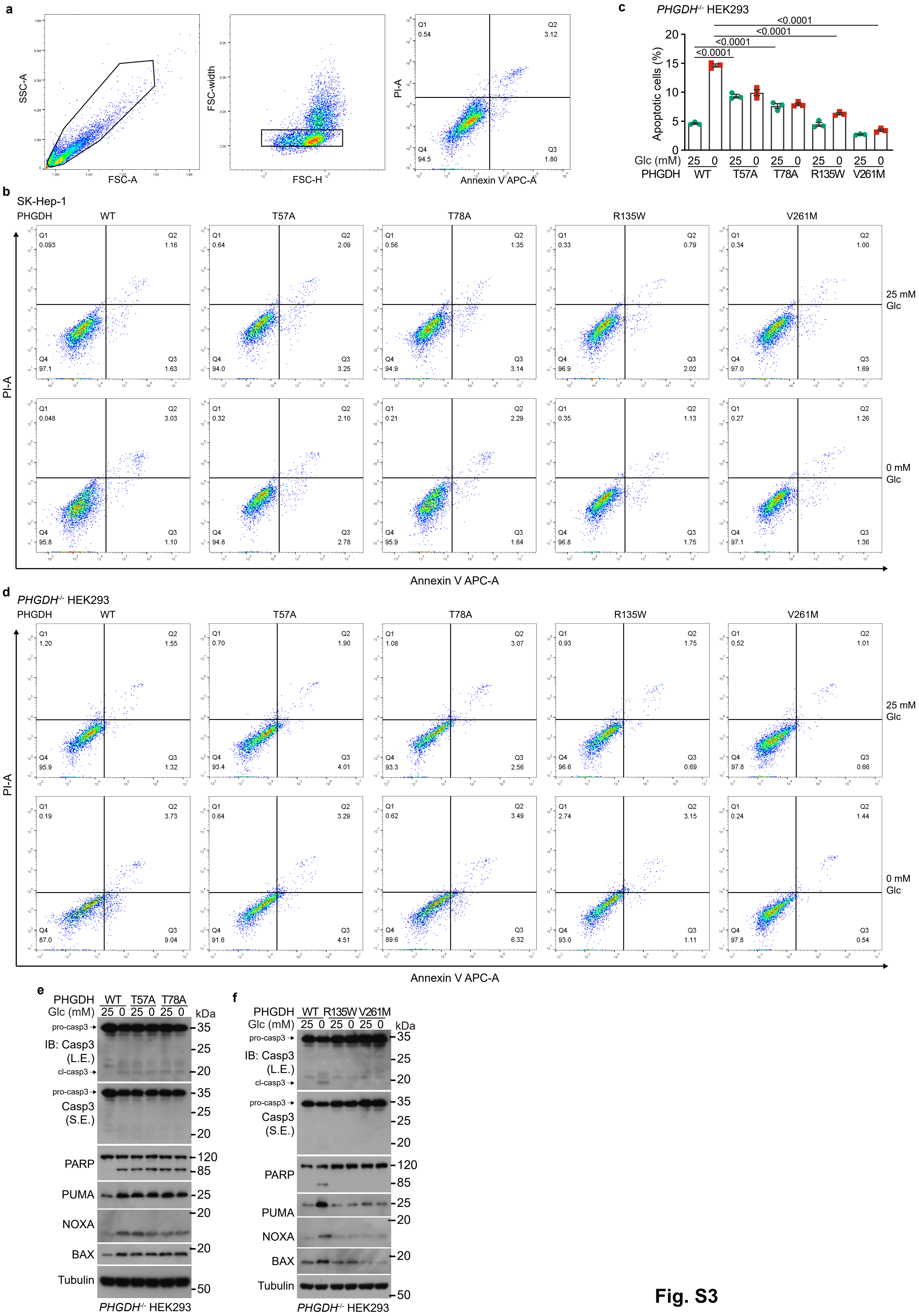
a
SSC-A
FSC-A
FSC-width
FSC-H
PI-A
Annexin V APC-A
c
PHGDH-/- HEK293
Apoptotic cells (%)
<0.0001
Glc (mM) 25 0 25 0 25 0 25 0 25 0
PHGDH WT T57A T78A R135W V261M
b
SK-Hep-1
PHGDH WT T57A T78A R135W V261M
25 mM Glc
0 mM Glc
PI-A
Annexin V APC-A
d
PHGDH-/- HEK293
PHGDH WT T57A T78A R135W V261M
25 mM Glc
0 mM Glc
PI-A
Annexin V APC-A
e
PHGDH WT T57A T78A
Glc (mM) 25 0 25 0 25 0
kDa
pro-casp3
IB: Casp3 (L.E.)
cl-casp3
Casp3 (S.E.)
PARP
PUMA
NOXA
BAX
Tubulin
PHGDH-/- HEK293
f
PHGDH WT R135W V261M
Glc (mM) 25 0 25 0 25 0
kDa
pro-casp3
IB: Casp3 (L.E.)
cl-casp3
Casp3 (S.E.)
PARP
PUMA
NOXA
BAX
Tubulin
PHGDH-/- HEK293

**Fig. S3**

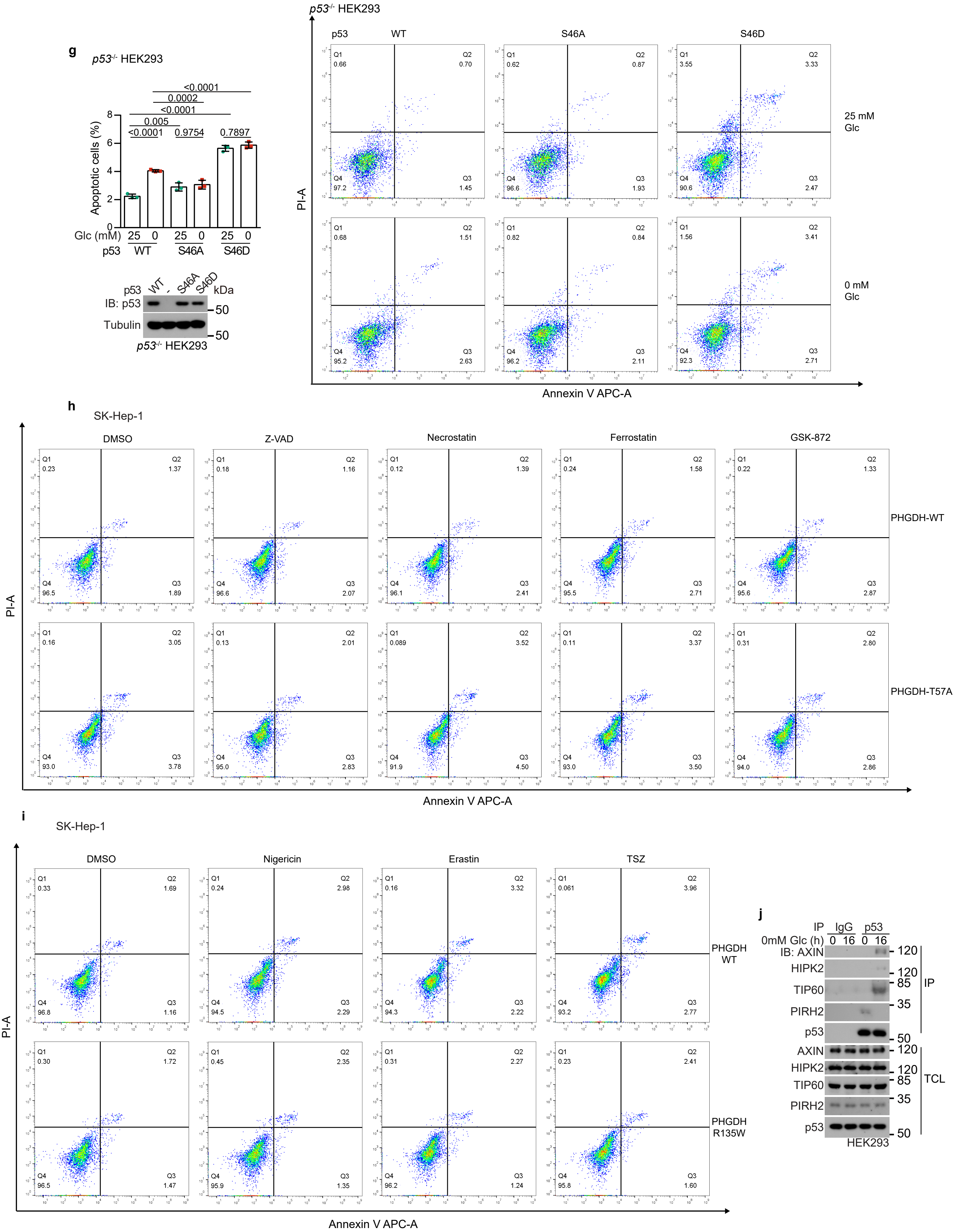
g
p53-/- HEK293
Apoptotic cells (%)
<0.0001
0.0002
<0.0001
0.005
<0.0001
0.9754
0.7897
Glc (mM) 25 0 25 0 25 0
p53 WT S46A S46D
p53 WT - S46A S46D kDa
IB: p53 -50
Tubulin -50
p53-/- HEK293
p53-/- HEK293
p53 WT S46A S46D
Q1 0.66 Q2 0.70 Q4 97.2 Q3 1.45
Q1 0.62 Q2 0.87 Q4 96.6 Q3 1.93
Q1 3.55 Q2 3.33 Q4 90.6 Q3 2.47
25 mM Glc
Q1 0.68 Q2 1.51 Q4 95.2 Q3 2.63
Q1 0.82 Q2 0.84 Q4 96.2 Q3 2.11
Q1 1.56 Q2 3.41 Q4 92.3 Q3 2.71
0 mM Glc
PI-A
Annexin V APC-A
h
SK-Hep-1
DMSO Z-VAD Necrostatin Ferrostatin GSK-872
Q1 0.23 Q2 1.37 Q4 96.5 Q3 1.89
Q1 0.18 Q2 1.16 Q4 96.6 Q3 2.07
Q1 0.12 Q2 1.39 Q4 96.1 Q3 2.41
Q1 0.24 Q2 1.58 Q4 95.5 Q3 2.71
Q1 0.22 Q2 1.33 Q4 95.6 Q3 2.87
PHGDH-WT
Q1 0.16 Q2 3.05 Q4 93.0 Q3 3.78
Q1 0.13 Q2 2.01 Q4 95.0 Q3 2.83
Q1 0.089 Q2 3.52 Q4 91.9 Q3 4.50
Q1 0.11 Q2 3.37 Q4 93.0 Q3 3.50
Q1 0.31 Q2 2.80 Q4 94.0 Q3 2.86
PHGDH-T57A
PI-A
Annexin V APC-A
i
SK-Hep-1
DMSO Nigericin Erastin TSZ
Q1 0.33 Q2 1.69 Q4 96.8 Q3 1.16
Q1 0.24 Q2 2.98 Q4 94.5 Q3 2.29
Q1 0.16 Q2 3.32 Q4 94.3 Q3 2.22
Q1 0.061 Q2 3.96 Q4 93.2 Q3 2.77
PHGDH WT
Q1 0.30 Q2 1.72 Q4 96.5 Q3 1.47
Q1 0.45 Q2 2.35 Q4 95.9 Q3 1.35
Q1 0.31 Q2 2.27 Q4 96.2 Q3 1.24
Q1 0.23 Q2 2.41 Q4 95.8 Q3 1.60
PHGDH R135W
PI-A
Annexin V APC-A
j
IP IgG p53
0mM Glc (h) 0 16 0 16
IB: AXIN -120
HIPK2 -120
TIP60 -85
PIRH2 -35
p53 -50
IP
AXIN -120
HIPK2 -120
TIP60 -85
PIRH2 -35
p53 -50
TCL
HEK293

**Fig. S3 (cont.)**

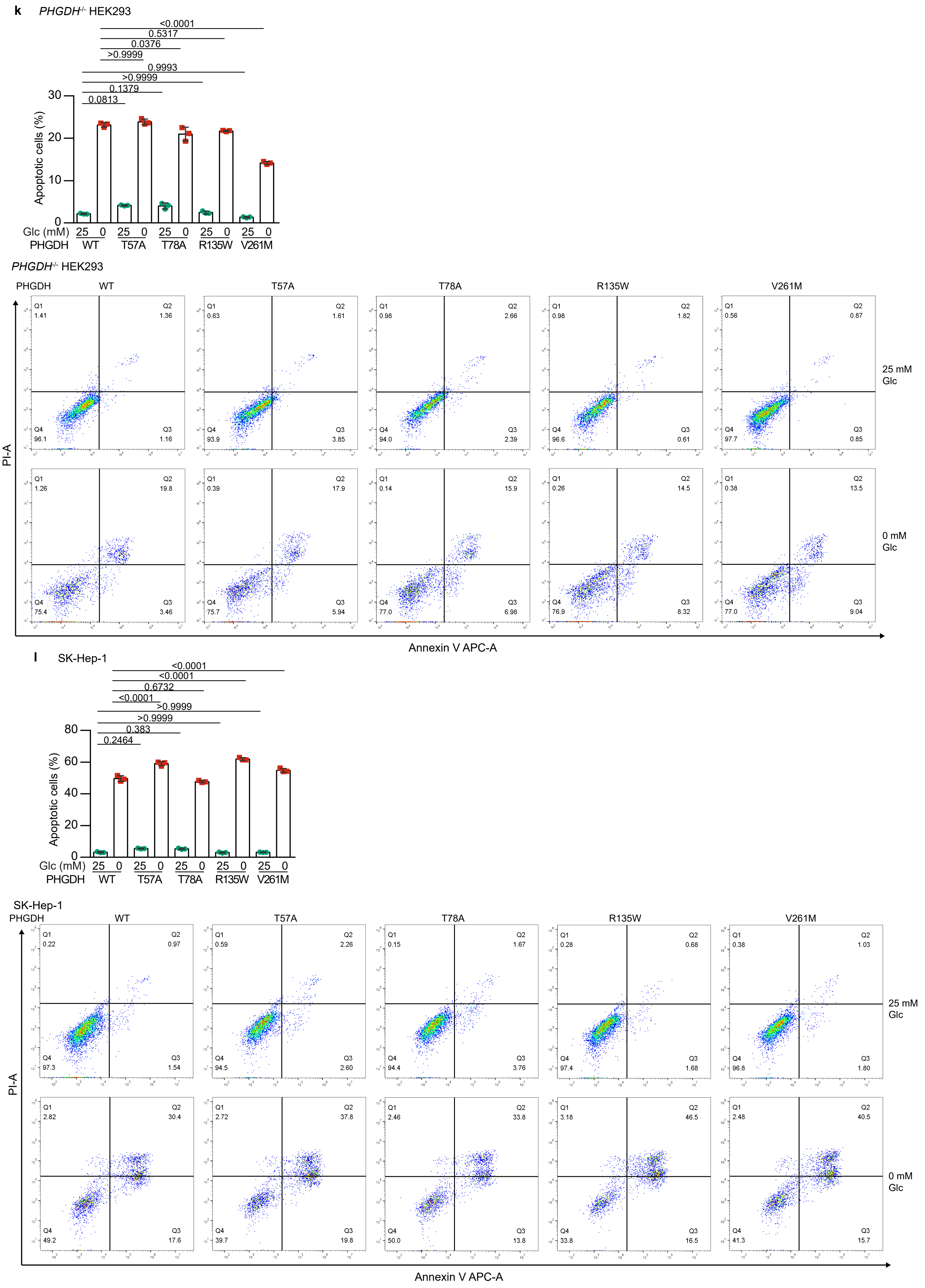

k
PHGDH-/- HEK293
<0.0001
0.5317
0.0376
>0.9999
0.9993
>0.9999
0.1379
0.0813
Apoptotic cells (%)
Glc (mM) 25 0 25 0 25 0 25 0 25 0
PHGDH WT T57A T78A R135W V261M
PHGDH-/- HEK293
PHGDH WT T57A T78A R135W V261M
Q1 1.41 Q2 1.36 Q4 96.1 Q3 1.16
Q1 0.63 Q2 1.61 Q4 93.9 Q3 3.85
Q1 0.98 Q2 2.66 Q4 94.0 Q3 2.39
Q1 0.98 Q2 1.82 Q4 96.6 Q3 0.61
Q1 0.56 Q2 0.87 Q4 97.7 Q3 0.85
25 mM Glc
Q1 1.26 Q2 19.8 Q4 75.4 Q3 3.46
Q1 0.39 Q2 17.9 Q4 75.7 Q3 5.94
Q1 0.14 Q2 15.9 Q4 77.0 Q3 6.98
Q1 0.26 Q2 14.5 Q4 76.9 Q3 8.32
Q1 0.38 Q2 13.5 Q4 77.0 Q3 9.04
0 mM Glc
PI-A
Annexin V APC-A
l
SK-Hep-1
<0.0001
<0.0001
0.6732
<0.0001
>0.9999
>0.9999
0.383
0.2464
Apoptotic cells (%)
Glc (mM) 25 0 25 0 25 0 25 0 25 0
PHGDH WT T57A T78A R135W V261M
SK-Hep-1
PHGDH WT T57A T78A R135W V261M
Q1 0.22 Q2 0.97 Q4 97.3 Q3 1.54
Q1 0.59 Q2 2.26 Q4 94.5 Q3 2.60
Q1 0.15 Q2 1.67 Q4 94.4 Q3 3.76
Q1 0.28 Q2 0.68 Q4 97.4 Q3 1.68
Q1 0.38 Q2 1.03 Q4 96.8 Q3 1.80
25 mM Glc
Q1 2.82 Q2 30.4 Q4 49.2 Q3 17.6
Q1 2.72 Q2 37.8 Q4 39.7 Q3 19.8
Q1 2.46 Q2 33.8 Q4 50.0 Q3 13.8
Q1 3.18 Q2 46.5 Q4 33.8 Q3 16.5
Q1 2.48 Q2 40.5 Q4 41.3 Q3 15.7
0 mM Glc
PI-A
Annexin V APC-A


**Fig. S3 (cont.)**

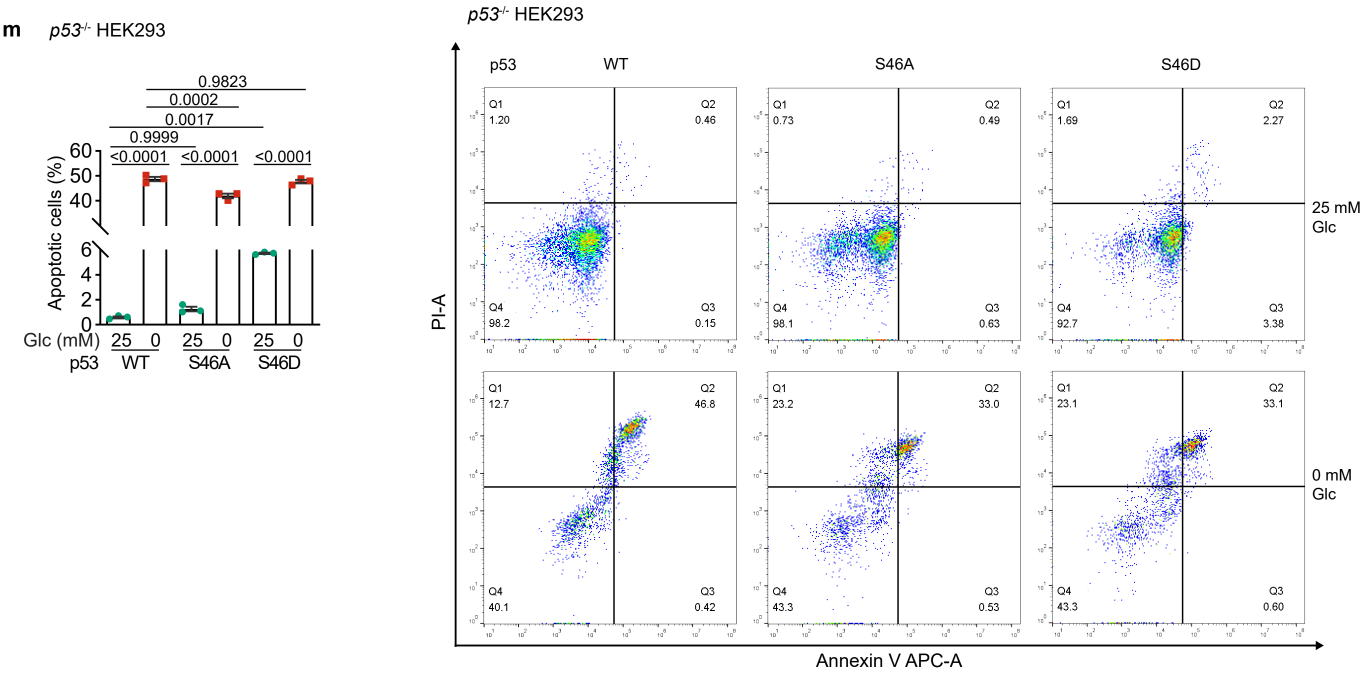


**Fig. S3 PHGDH unable to bind 3-PGA constitutively promotes apoptosis. a** Gating strategies used for quantifying the populations of apoptotic cells. During the analysis, intact cells from each sample were selected by FSC-A and SSC-A (left, using a linear scale), followed by FSC-H and FSC-width to exclude doublets (middle, using a linear scale). The fluorescence intensities of propidium iodide (PI) and Annexin V-FITC were then determined and displayed as density plots (right, using a log scale). The plot was divided into four quadrants (Q1, Q2, Q3, and Q4) in which Q2 and Q3 contain apoptotic cells while Q1 and Q2 are PI-positive cells. The percentage of apoptotic cells was then calculated. **b** Representative density plots of Fig. 4a. **c-f** 3-PGA-unoccupied PHGDH mutants induce apoptosis in HEK293 cells starved for glucose. Cells were treated as in Fig. 4a, except that the *PHGDH*$^{-/-}$ HEK293 cells were used. The levels of apoptotic cells were then determined via flow cytometry (**c**, data are means ± SD, n = 3, with p values calculated by two-way ANOVA, followed by Tukey; see representative density plots in **d**), and the apoptotic markers by immunoblotting (**e** and **f**). **g** Glucose starvation-induced apoptosis depends on the phosphorylation of p53-Ser46. The *p53*$^{-/-}$ HEK293 cells with p53-S46A or p53-S46D expressed under a doxycycline-inducible promoter were used and treated as in Fig. 4a. The levels of apoptotic cells were determined via flow cytometry (data are means ± SD, n = 3, with p values calculated by two-way ANOVA, followed by Sidak; see validation data of p53 expression levels in the right panel, and the representative density plots in the left panel). **h**, **i** Representative density plots of Fig. 4d, e. **j-m** PHGDH and Ser46-p53 phosphorylation are not required for apoptosis induced by prolonged glucose starvation. *PHGDH*$^{-/-}$ HEK293 cells (**k**) or SK-Hep-1 cells (**l**) with PHGDH-T57A, PHGDH-T78A, PHGDH-R135W or PHGDH-V261M expression, or *p53*$^{-/-}$ HEK293 cells with p53-S46A or p53-S46D expression (**m**), all under the doxycycline-inducible promoter, incubated in DMEM or glucose-free DMEM, both containing doxycycline (100 ng/ml), for 16 h, followed by determining the levels of apoptotic cells via flow cytometry. Data are means ± SD, n = 3, with p values calculated by two-way ANOVA, followed by Sidak; see representative density plots in the lower panel. See also PHGDH-AXIN-p53-HIPK2 complex in **j**. Experiments in this figure were performed three times.
