# Supplementary material for: Low glucose metabolite 3-phosphoglycerate switches PHGDH from serine synthesis to p53 activation to control cell fate
Source: Cell Res. 2023 Sep 19;33(11):835–50. doi: 10.1038/s41422-023-00874-4 (PMC10624847; doi:10.1038/s41422-023-00874-4)
Supplement: Supplementary file 4 — Fig. S4 [file 41422_2023_874_MOESM4_ESM.pdf]

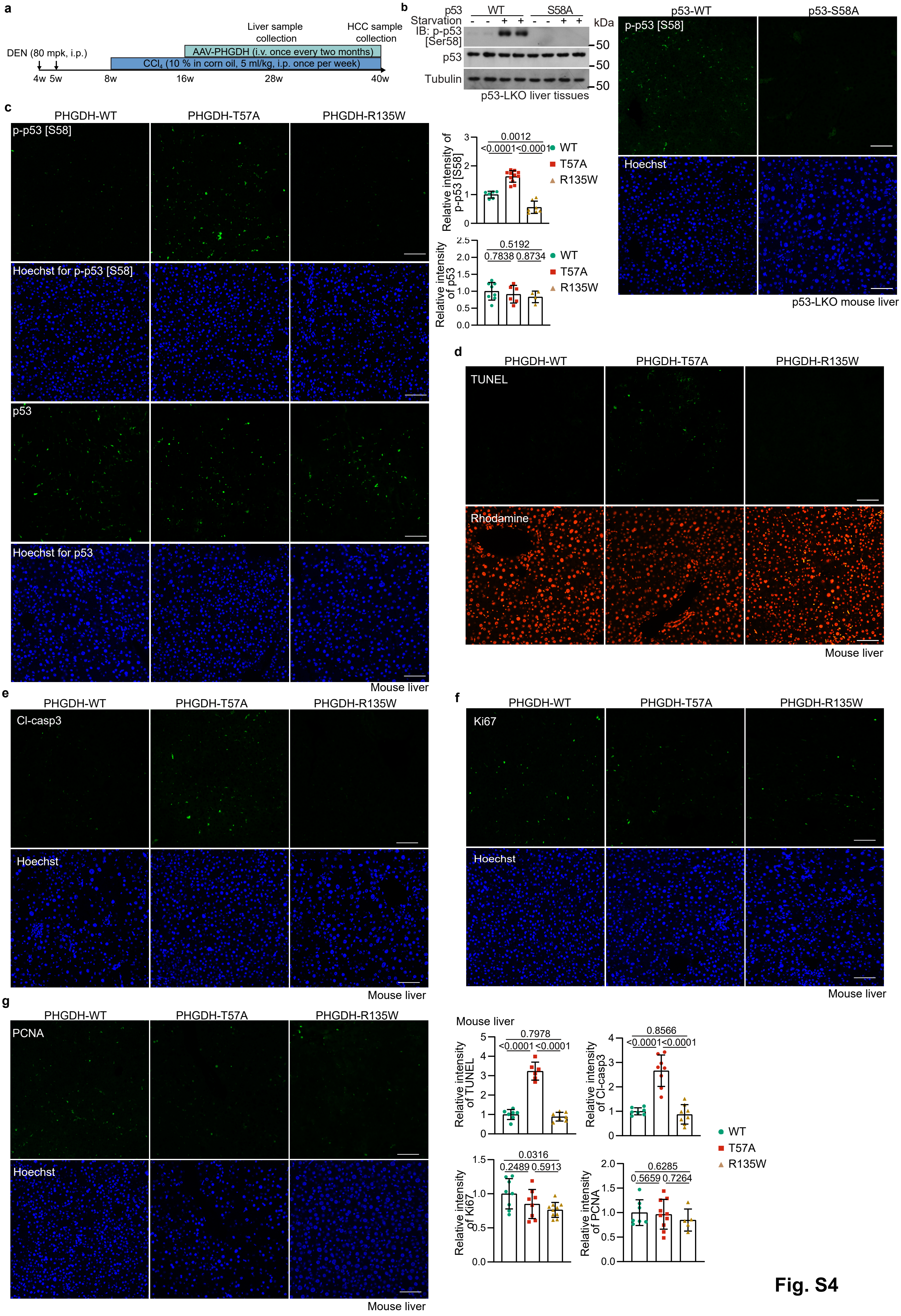
a
DEN (80 mpk, i.p.)
Liver sample collection
HCC sample collection
AAV-PHGDH (i.v. once every two months)
CCl4 (10 % in corn oil, 5 ml/kg, i.p. once per week)
4w 5w 8w 16w 28w 40w
b
p53 WT S58A
Starvation - - + + - - + +
IB: p-p53 [Ser58]
p53
Tubulin
kDa
50
50
50
p53-LKO liver tissues
p53-WT p53-S58A
p-p53 [S58]
Hoechst
p53-LKO mouse liver
c
PHGDH-WT PHGDH-T57A PHGDH-R135W
p-p53 [S58]
Hoechst for p-p53 [S58]
p53
Hoechst for p53
Mouse liver
Relative intensity of p-p53 [S58]
0.0012
<0.0001 <0.0001
WT
T57A
R135W
Relative intensity of p53
0.5192
0.7838 0.8734
d
PHGDH-WT PHGDH-T57A PHGDH-R135W
TUNEL
Rhodamine
Mouse liver
e
PHGDH-WT PHGDH-T57A PHGDH-R135W
Cl-casp3
Hoechst
Mouse liver
f
PHGDH-WT PHGDH-T57A PHGDH-R135W
Ki67
Hoechst
Mouse liver
g
PHGDH-WT PHGDH-T57A PHGDH-R135W
PCNA
Hoechst
Mouse liver
Mouse liver
Relative intensity of TUNEL
0.7978
<0.0001 <0.0001
Relative intensity of Cl-casp3
0.8566
<0.0001 <0.0001
Relative intensity of Ki67
0.0316
0.2489 0.5913
Relative intensity of PCNA
0.6285
0.5659 0.7264
WT
T57A
R135W

Fig. S4

Fig. S4 PHGDH mediates apoptosis in mouse liver by low glucose. a A schematic showing the experimental timeline for the establishment of DEN/CCl_4-induced HCC mouse model with hepatic expression of PHGDH mutants. **b** Validation data showing the specificity of antibody against p-Ser58-p53 (equivalent to human p-Ser46-p53). The liver-specific *Trp53* knockout (p53-LKO) mice were injected with AAV-p53 or AAV-p53-S58A. Two weeks after AAV injection, followed by immunoblotting (left panel) or immunohistochemistry (right panel) analysis of liver tissues. **c** 3-PGA binding of PHGDH controls p-Ser58-p53 in liver tissues. The DEN/CCl_4-induced HCC mice expressing PHGDH mutants were sacrificed at 28 weeks old (at which no discernible HCC was formed), followed by determination of hepatic p-Ser58-p53 by immunohistochemistry. Data are means ± SD, n = 6-11 fields from 6 mice, with p values calculated by one-way ANOVA, followed by Tukey. **d-g** 3-PGA binding of PHGDH controls apoptosis and proliferation in liver tissues. Mice were treated as in (**c**), and liver tissues were excised, followed by determination of the apoptotic activity (TUNEL, in **d**; and cl-casp3, in **e**) and proliferative markers (Ki67, in **f**; and PCNA, in **g**) by immunohistochemistry. Data are means ± SD, n = 5-10 fields from 7 mice, with p values calculated by one-way ANOVA, followed by Tukey. The scale bar in this figure is 10 μm. Experiments in this figure were performed three times.
