# Supplementary material for: Low glucose metabolite 3-phosphoglycerate switches PHGDH from serine synthesis to p53 activation to control cell fate
Source: Cell Res. 2023 Sep 19;33(11):835–50. doi: 10.1038/s41422-023-00874-4 (PMC10624847; doi:10.1038/s41422-023-00874-4)
Supplement: Supplementary file 5 — Fig. S5 [file 41422_2023_874_MOESM5_ESM.pdf]

a

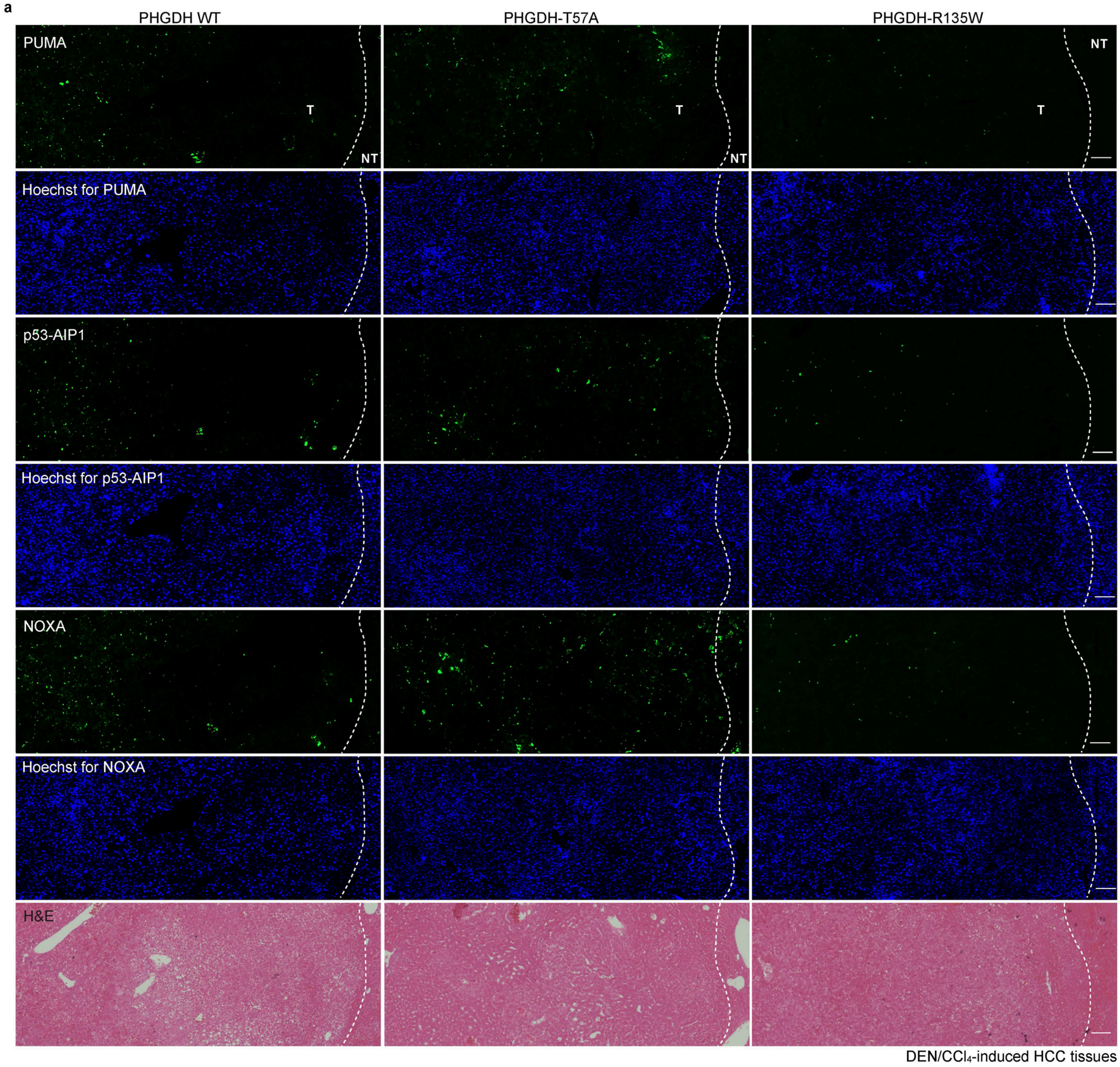
PHGDH WT
PHGDH-T57A
PHGDH-R135W
PUMA
T
NT
Hoechst for PUMA
p53-AIP1
Hoechst for p53-AIP1
NOXA
Hoechst for NOXA
H&E
DEN/CCl4-induced HCC tissues

b

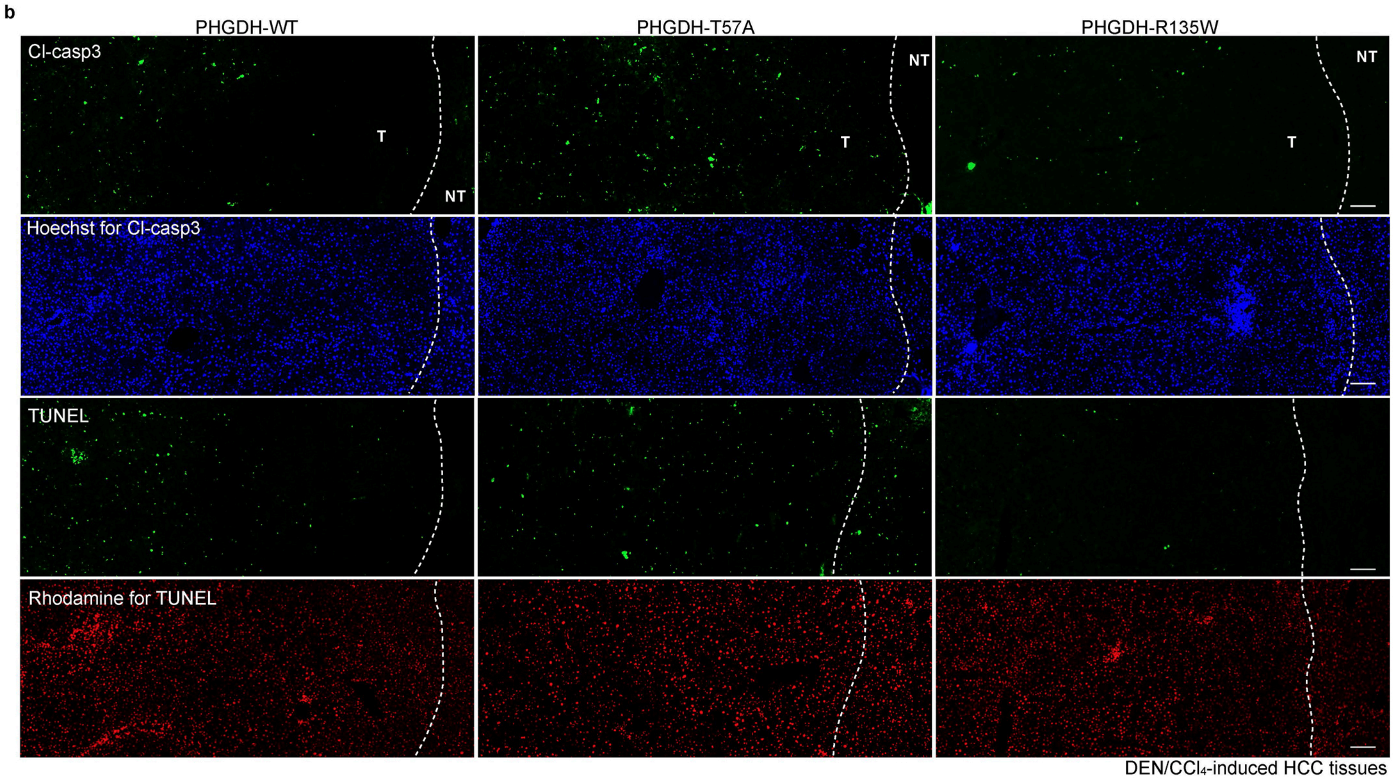
PHGDH-WT
PHGDH-T57A
PHGDH-R135W
Cl-casp3
T
NT
Hoechst for Cl-casp3
TUNEL
Rhodamine for TUNEL
DEN/CCl4-induced HCC tissues

Fig. S5

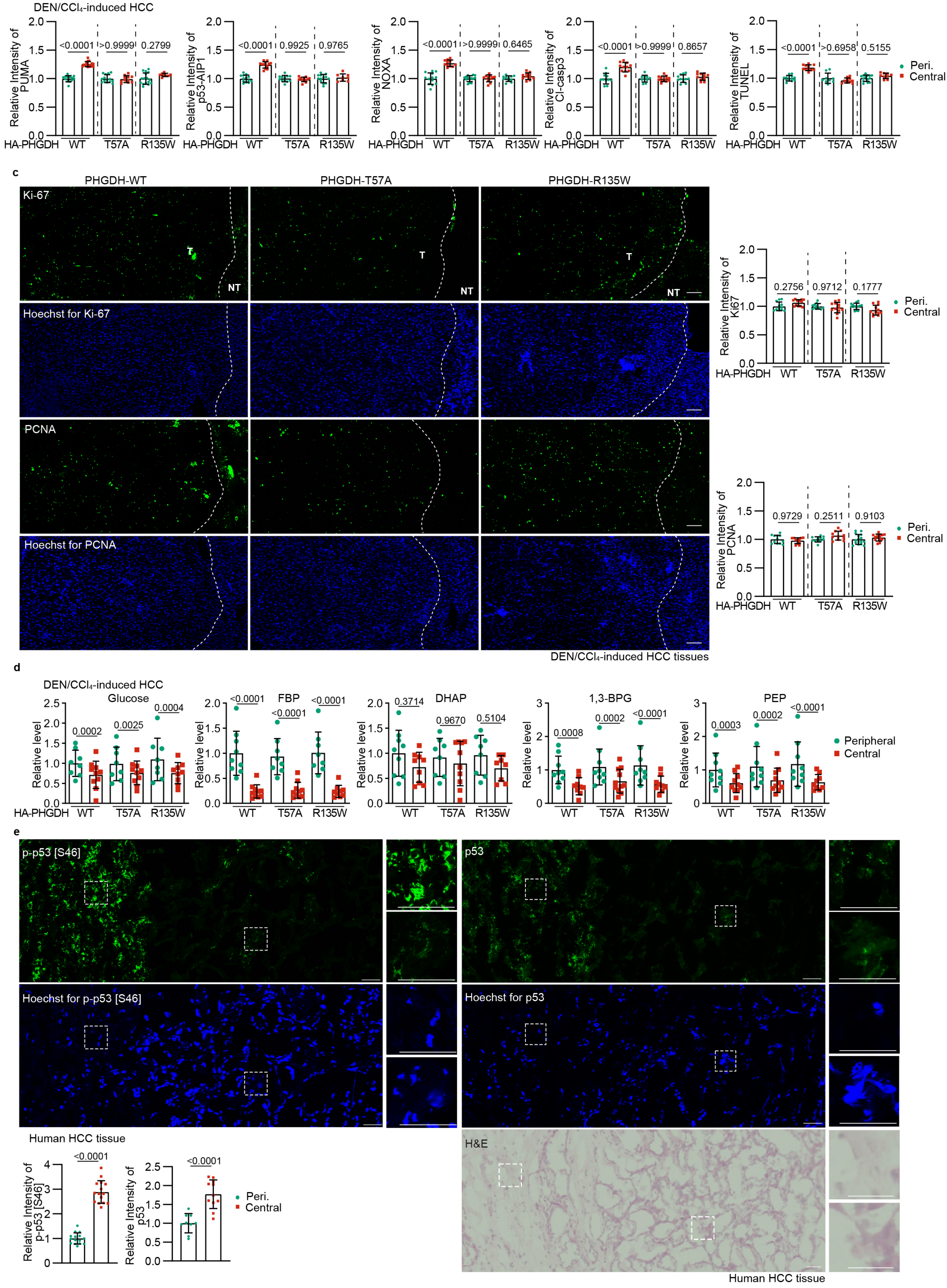

DEN/CCl4-induced HCC
Relative Intensity of PUMA
Relative Intensity of p53-AIP1
Relative Intensity of NOXA
Relative Intensity of Cl-casp3
Relative Intensity of TUNEL
HA-PHGDH
WT
T57A
R135W
<0.0001
>0.9999
0.2799
0.9925
0.9765
0.6465
0.8657
>0.6958
0.5155
Peri.
Central
c
PHGDH-WT
PHGDH-T57A
PHGDH-R135W
Ki-67
Hoechst for Ki-67
PCNA
Hoechst for PCNA
T
NT
Relative Intensity of Ki67
0.2756
0.9712
0.1777
Relative Intensity of PCNA
0.9729
0.2511
0.9103
DEN/CCl4-induced HCC tissues
d
DEN/CCl4-induced HCC
Glucose
FBP
DHAP
1,3-BPG
PEP
Relative level
0.0002
0.0025
0.0004
0.3714
0.9670
0.5104
0.0008
0.0003
Peripheral
Central
e
p-p53 [S46]
p53
Hoechst for p-p53 [S46]
Hoechst for p53
H&E
Human HCC tissue
Relative Intensity of p-p53 [S46]
Relative Intensity of p53

Fig. S5 (cont.)

Fig. S5 PHGDH mediates inhibition of tumor growth by low glucose. a-c 3-PGA binding of PHGDH controls apoptosis and proliferation in HCC tissues. Livers from HCC mice expressing PHGDH mutants (induced as in Fig. 5a) were excised, followed by determination of the apoptotic activity (**a**, **b**) and proliferative markers (**c**) by immunohistochemistry. Data are means ± SD, n = 8-16 fields from 7 mice, with p values calculated by one-way ANOVA, followed by Dunnet (**a**) or Tukey (**b**, **c**). **d** Levels of glycolytic intermediates in peripheral and central regions of HCC. Experiments were performed as in Fig. 5d, and data are means ± SD, n = 18-24, with p values calculated by one-way ANOVA, followed by Tukey. **e** Increased levels of apoptosis in the center of human HCC tissues. Data are means ± SD, n = 9-14 fields from 8 patients, with p values calculated by two-sided Student's *t*-test. Experiments were performed as in Fig. 5a, except that the human HCC tissues were stained for p-Ser46-p53. The scale bar in **a-c** is 100 μm and **e** 40 μm. Experiments in this figure were performed three times.
