# Supplementary material for: Low glucose metabolite 3-phosphoglycerate switches PHGDH from serine synthesis to p53 activation to control cell fate
Source: Cell Res. 2023 Sep 19;33(11):835–50. doi: 10.1038/s41422-023-00874-4 (PMC10624847; doi:10.1038/s41422-023-00874-4)
Supplement: Supplementary file 6 — Fig. S6 [file 41422_2023_874_MOESM6_ESM.pdf]

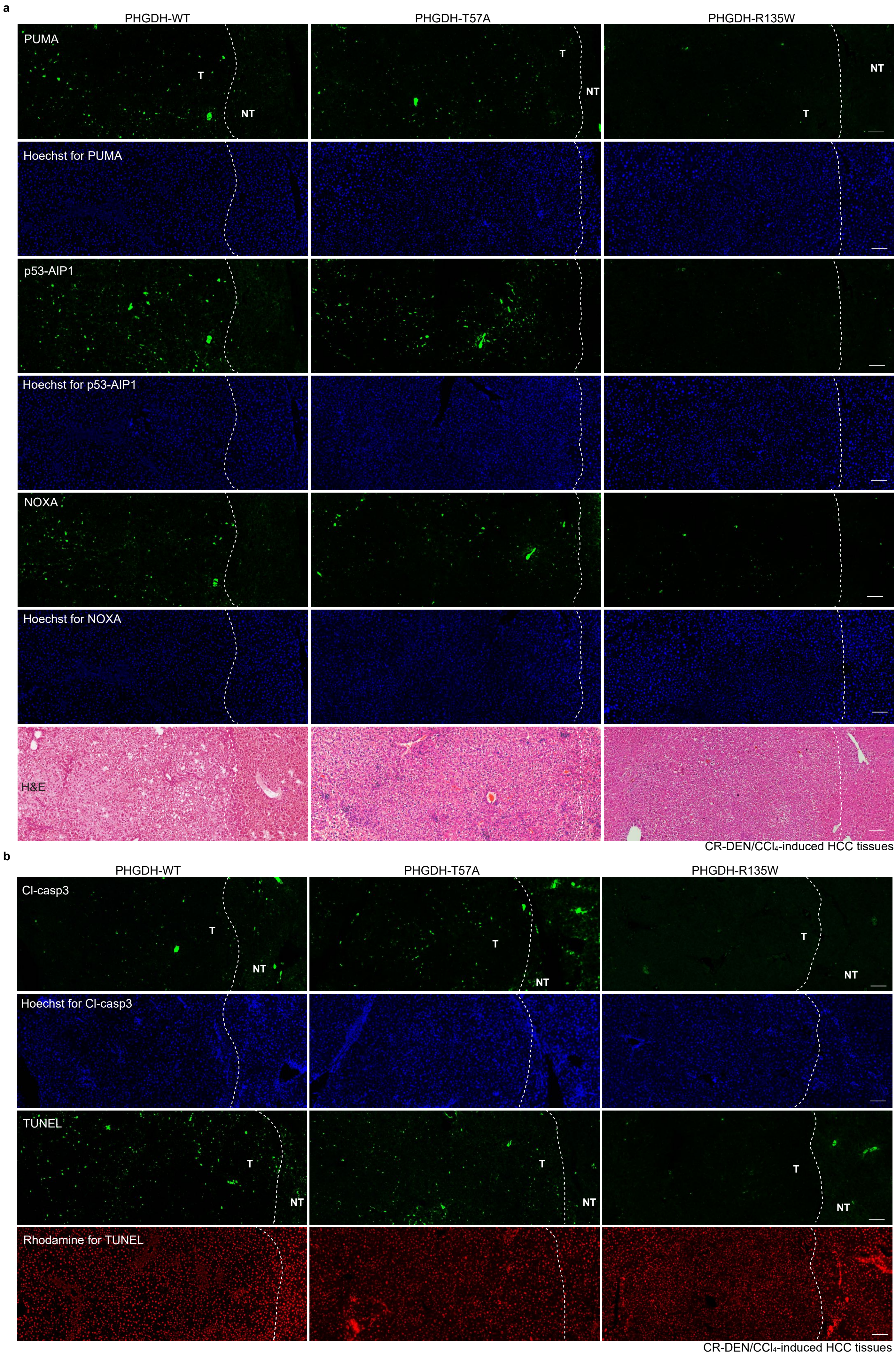
a
PHGDH-WT
PHGDH-T57A
PHGDH-R135W
PUMA
T
NT
Hoechst for PUMA
p53-AIP1
Hoechst for p53-AIP1
NOXA
Hoechst for NOXA
H&E
CR-DEN/CCl_4-induced HCC tissues
b
PHGDH-WT
PHGDH-T57A
PHGDH-R135W
Cl-casp3
T
NT
Hoechst for Cl-casp3
TUNEL
Rhodamine for TUNEL
CR-DEN/CCl_4-induced HCC tissues

Fig. S6

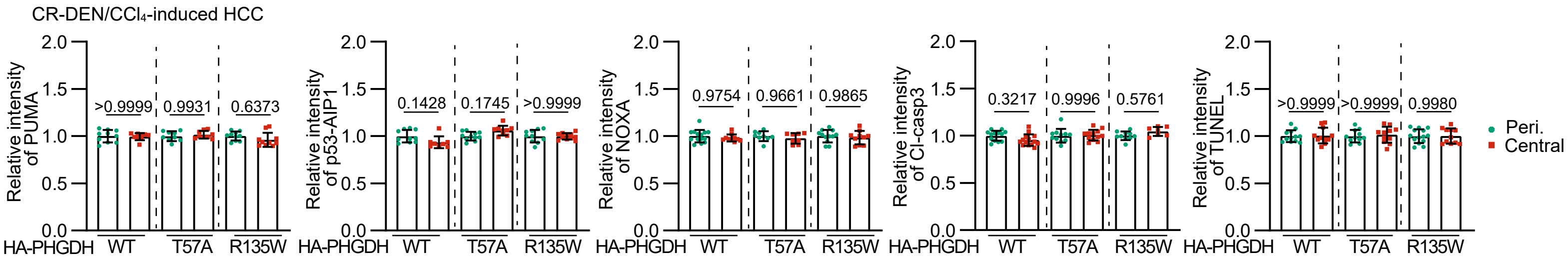

c

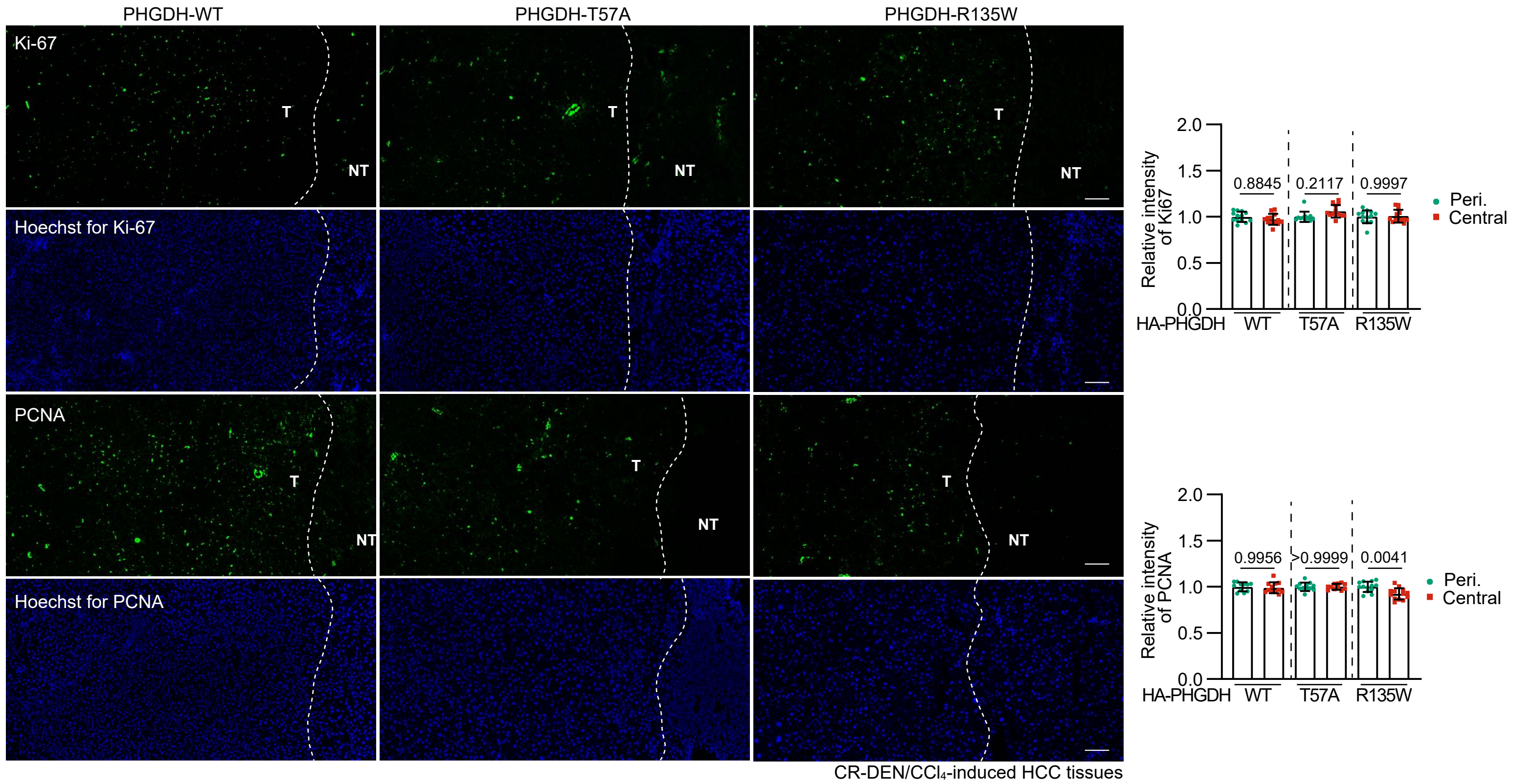

Fig. S6 Caloric restriction mimics the effects of PHGDH-T57A on HCC suppression. a-c CR promotes apoptosis and suppresses proliferation in HCC tissues. Livers from HCC mice expressing PHGDH mutants (induced as in Fig. 5a, except that the mice were also subjected to CR at 16 weeks old) were excised, followed by determination of the apoptotic activity (**a**, **b**) and proliferative markers (**c**) by immunohistochemistry. Data are means ± SD, n = 8-14 fields from 7 mice, with p values calculated by one-way ANOVA, followed by Tukey. The scale bar in this figure is 100 µm. Experiments in this figure were performed three times.
