# Supplementary material for: Low glucose metabolite 3-phosphoglycerate switches PHGDH from serine synthesis to p53 activation to control cell fate
Source: Cell Res. 2023 Sep 19;33(11):835–50. doi: 10.1038/s41422-023-00874-4 (PMC10624847; doi:10.1038/s41422-023-00874-4)
Supplement: Supplementary file 7 — Fig. S7 [file 41422_2023_874_MOESM7_ESM.pdf]

a

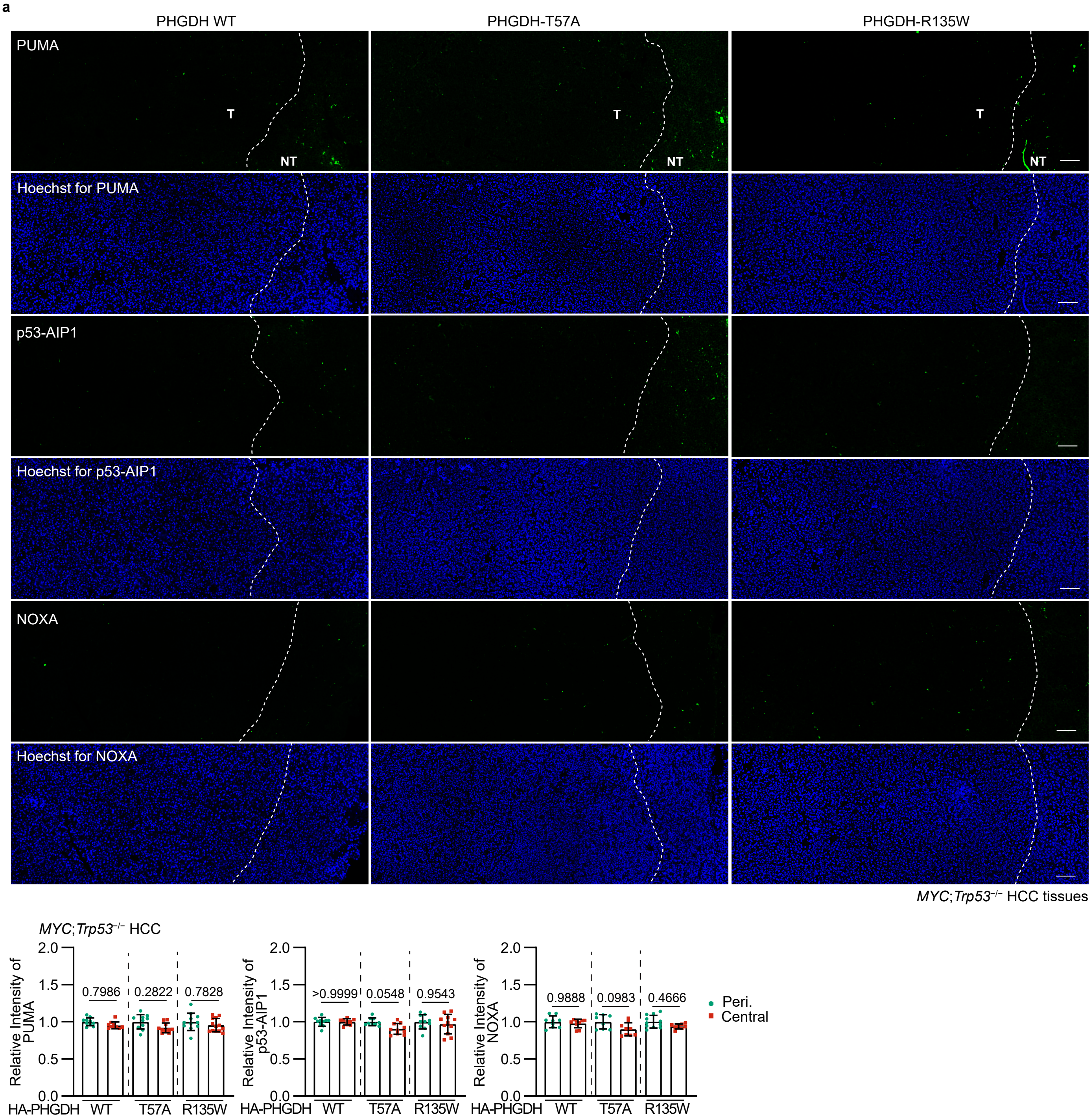

Fig. S7 PHGDH modulates HCC growth through p53. a Liver-specific knockout of *Trp53* abolishes the effects of PHGDH on apoptosis. Livers from HCC mice expressing PHGDH mutants (induced as in Fig. 7a) were excised, followed by determination of the apoptotic markers by immunohistochemistry. Data are means ± SD, n = 8-13 fields from 7 mice, with p values calculated by one-way ANOVA, followed by Tukey. The scale bar in this figure is 100 µm. Experiments in this figure were performed three times.
