# Supplementary material for: Low glucose metabolite 3-phosphoglycerate switches PHGDH from serine synthesis to p53 activation to control cell fate
Source: Cell Res. 2023 Sep 19;33(11):835–50. doi: 10.1038/s41422-023-00874-4 (PMC10624847; doi:10.1038/s41422-023-00874-4)

Blots, as shown below, were cut into slices before incubation with primary antibodies. The Pierce™ Prestained Protein MW Marker, Cat. 26612, from ThermoFisher Scientific, was used as the protein marker.

Fig. 1a

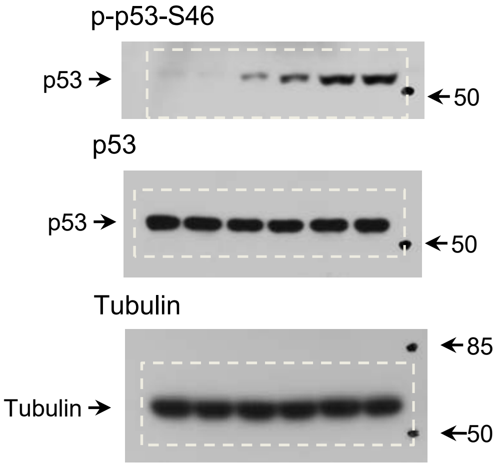

Fig. 1b

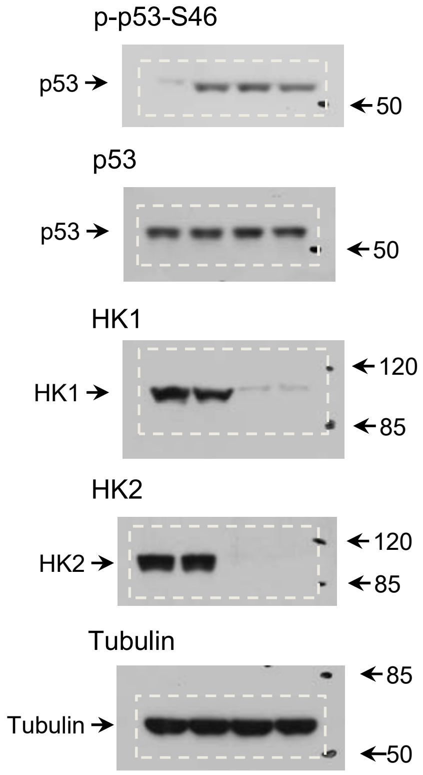

Fig. 1c

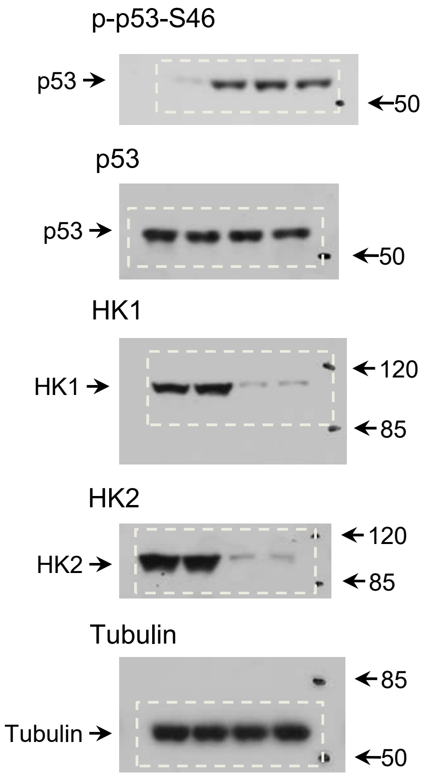

Fig. 1d

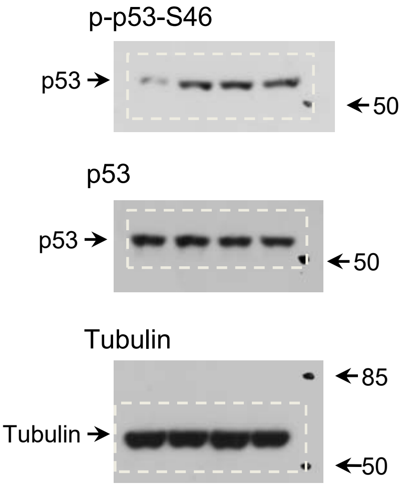

Fig. 1e

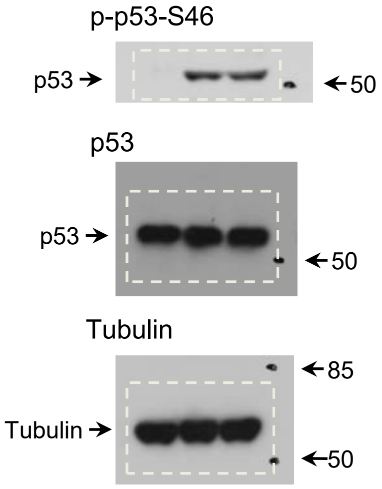

Fig. 1f

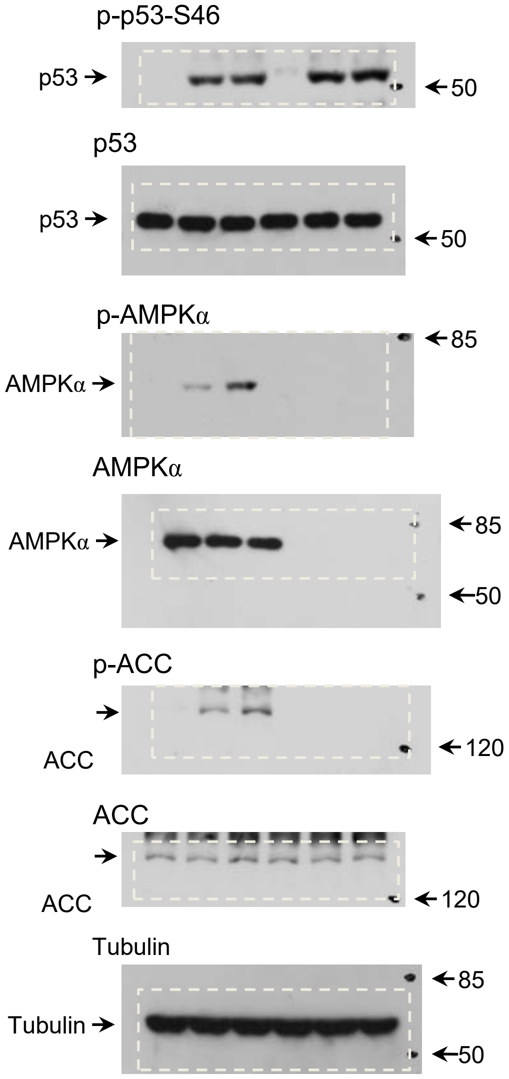

Fig. 1g

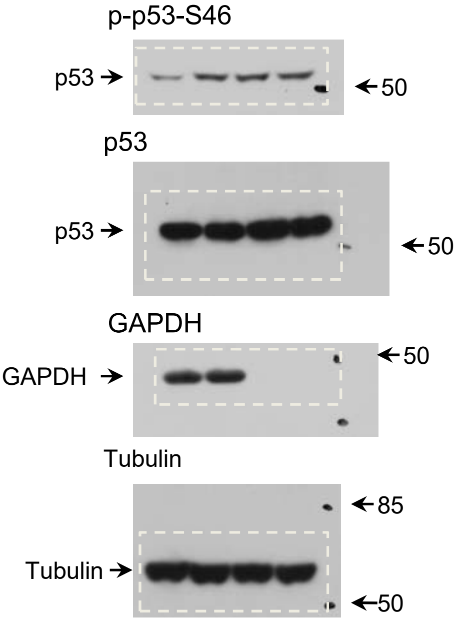

Fig. 1h

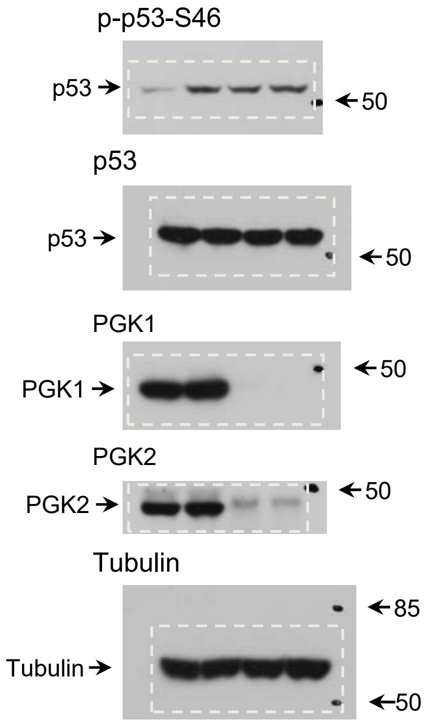

Fig. 1i

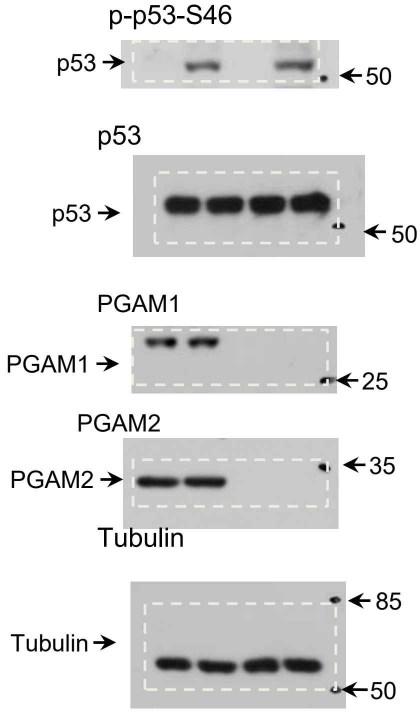

Fig. 1j

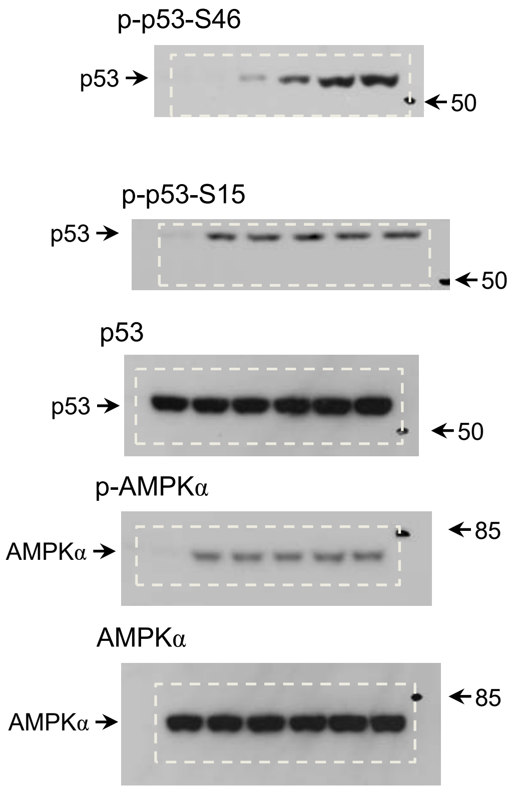

Fig. 2a (left)

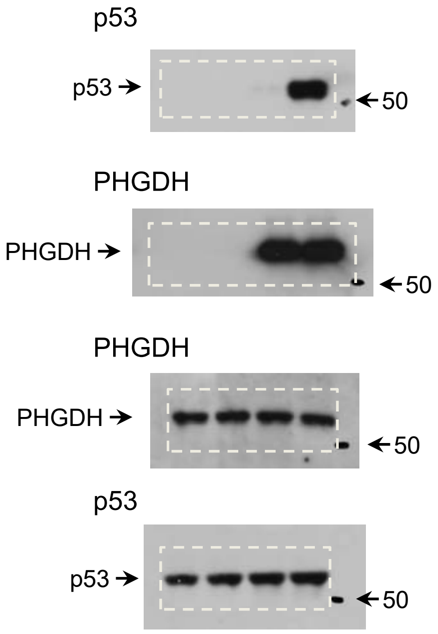

Fig. 2a (right)

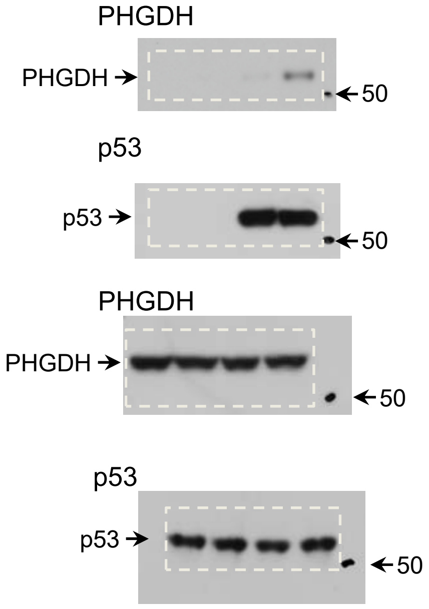

Fig. 2b

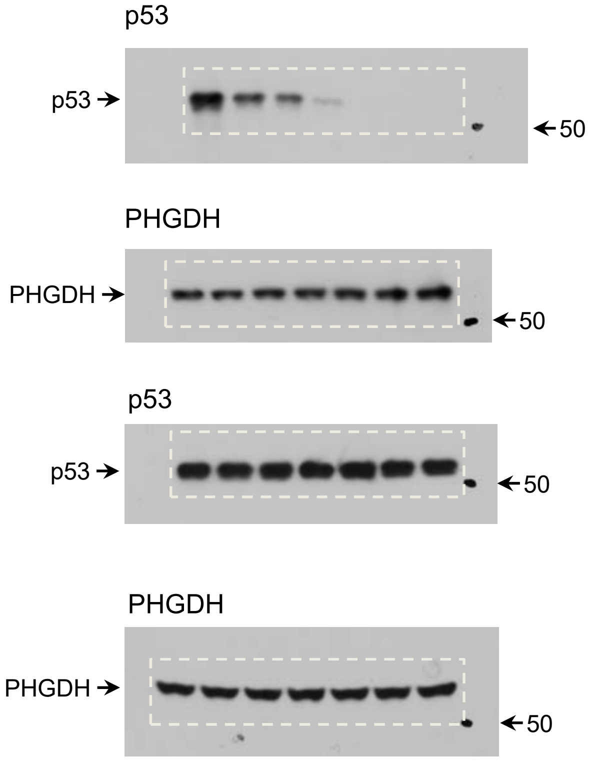

Fig. 2c

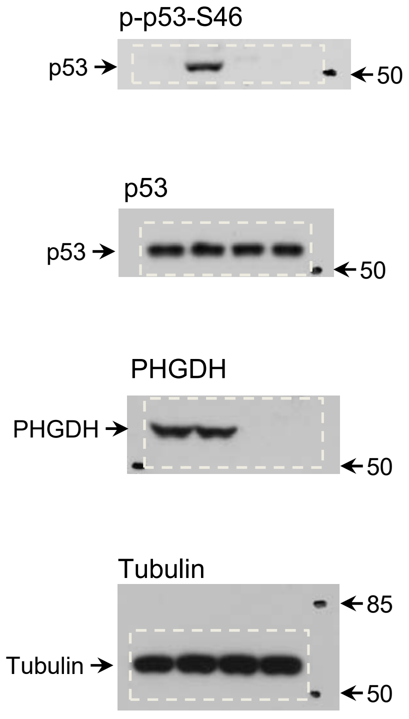

Fig. 2d

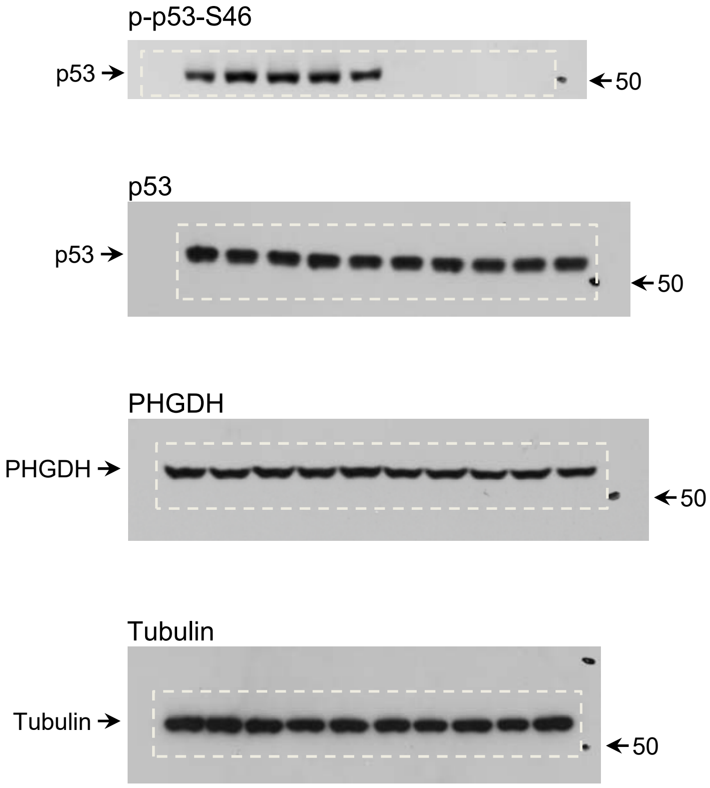
p-p53-S46
p53
50
p53
p53
50
PHGDH
PHGDH
50
Tubulin
Tubulin
50

Fig. 2e

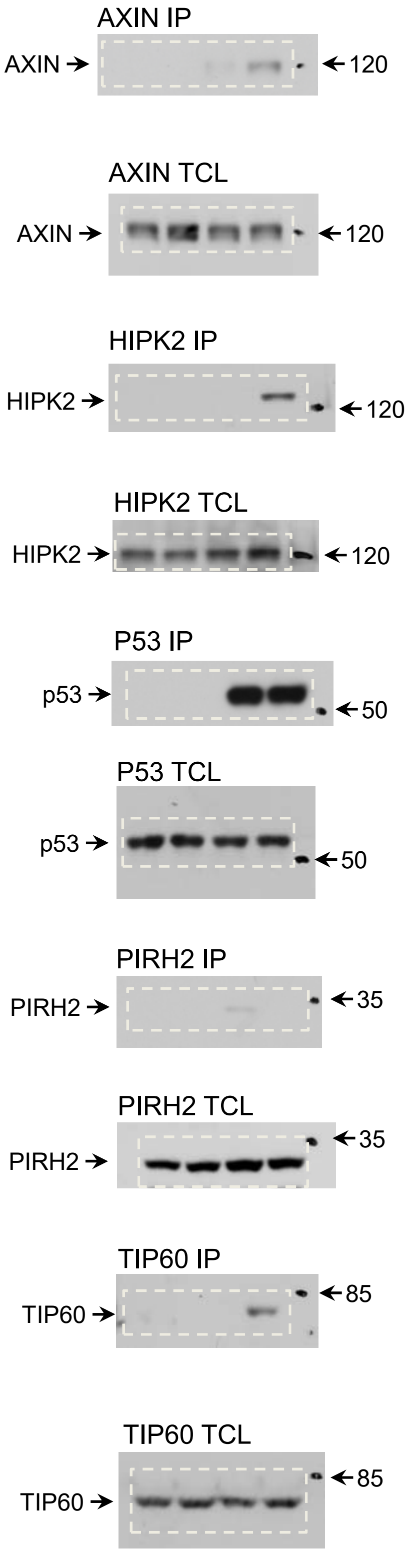
AXIN IP
AXIN
120
AXIN TCL
AXIN
120
HIPK2 IP
HIPK2
120
HIPK2 TCL
HIPK2
120
P53 IP
p53
50
P53 TCL
p53
50
PIRH2 IP
PIRH2
35
PIRH2 TCL
PIRH2
35
TIP60 IP
TIP60
85
TIP60 TCL
TIP60
85

Fig. 2f

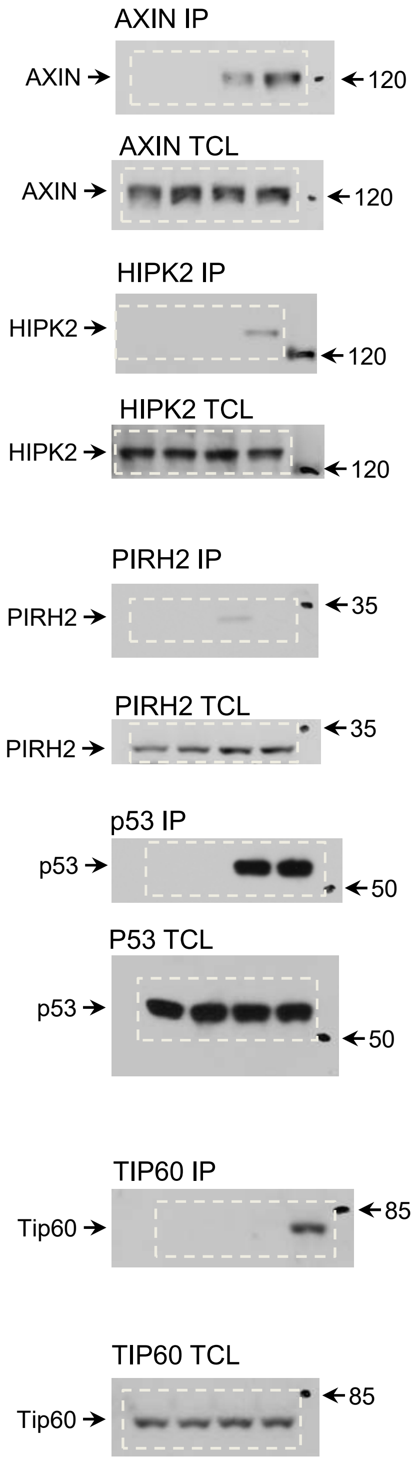

Fig. 2h

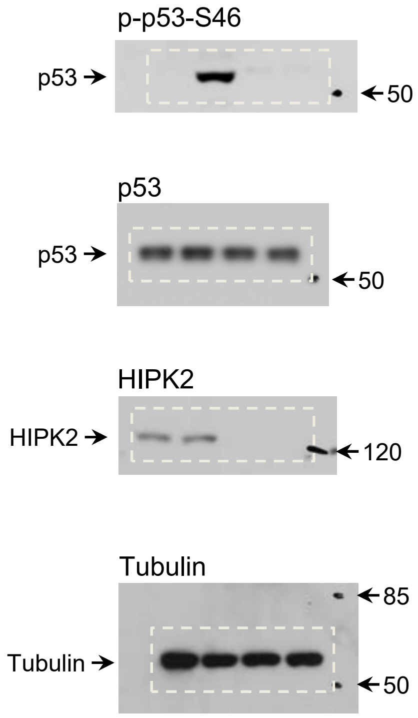

Fig. 2g

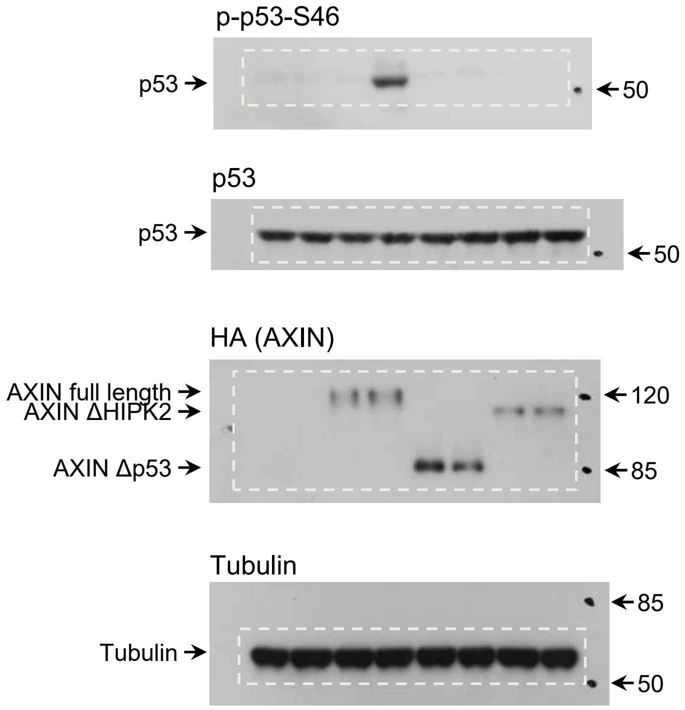

Fig. 3a

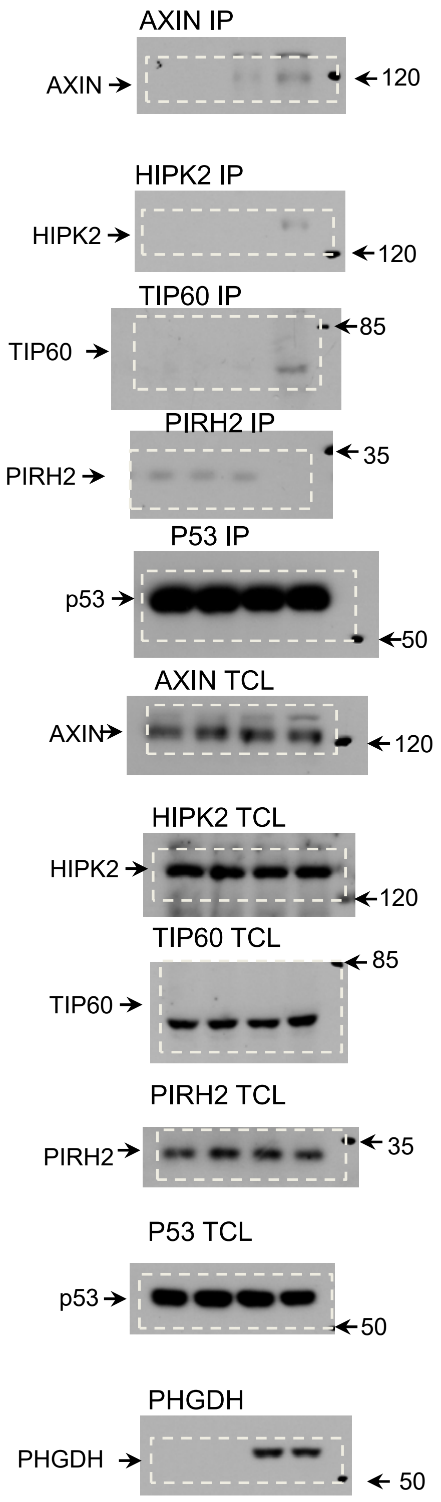
AXIN IP
AXIN
120
HIPK2 IP
HIPK2
120
TIP60 IP
TIP60
85
PIRH2 IP
PIRH2
35
P53 IP
p53
50
AXIN TCL
AXIN
120
HIPK2 TCL
HIPK2
120
TIP60 TCL
TIP60
85
PIRH2 TCL
PIRH2
35
P53 TCL
p53
50
PHGDH
PHGDH
50

Fig. 3b (upper)

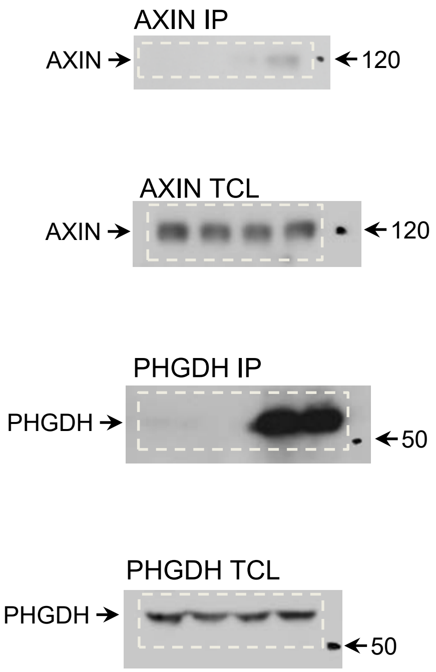

Fig. 3b (lower)

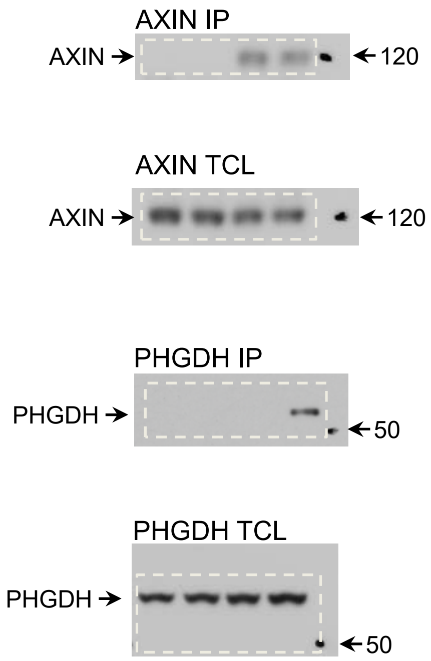

Fig. 3c (upper)

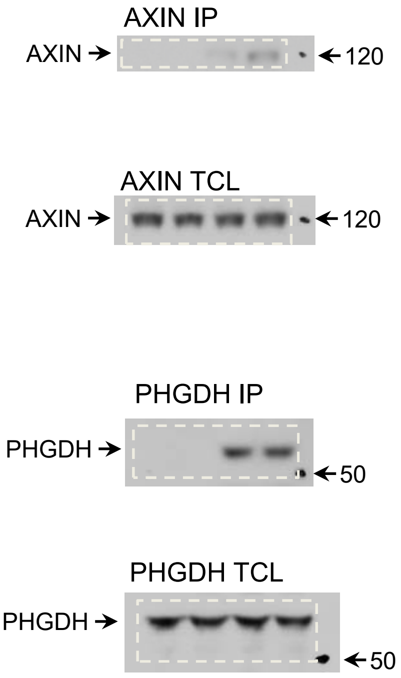

Fig. 3c (lower)

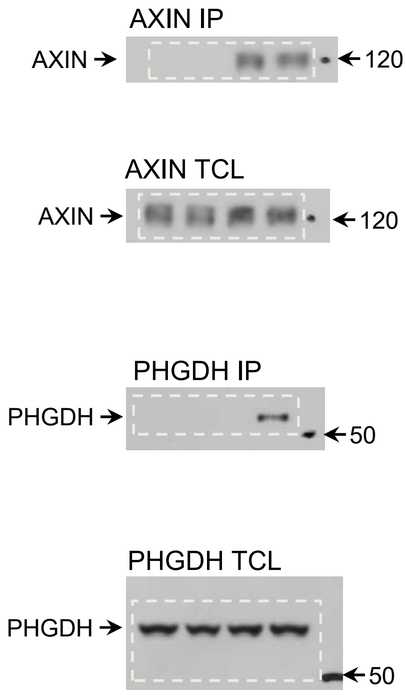

Fig. 3d

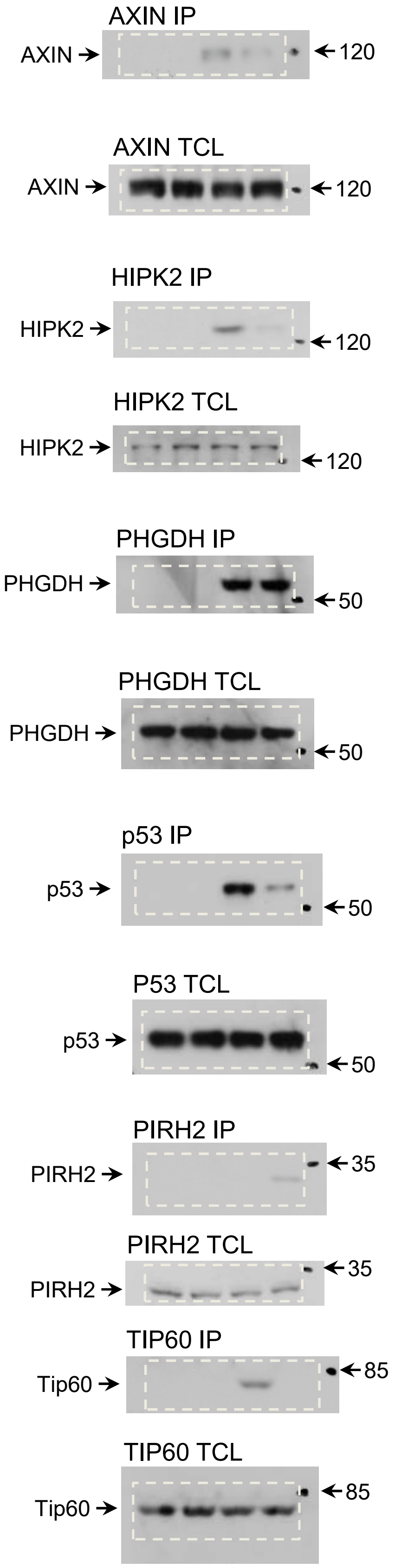
AXIN IP
AXIN
120
AXIN TCL
AXIN
120
HIPK2 IP
HIPK2
120
HIPK2 TCL
HIPK2
120
PHGDH IP
PHGDH
50
PHGDH TCL
PHGDH
50
p53 IP
p53
50
P53 TCL
p53
50
PIRH2 IP
PIRH2
35
PIRH2 TCL
PIRH2
35
TIP60 IP
Tip60
85
TIP60 TCL
Tip60
85

Fig. 3e

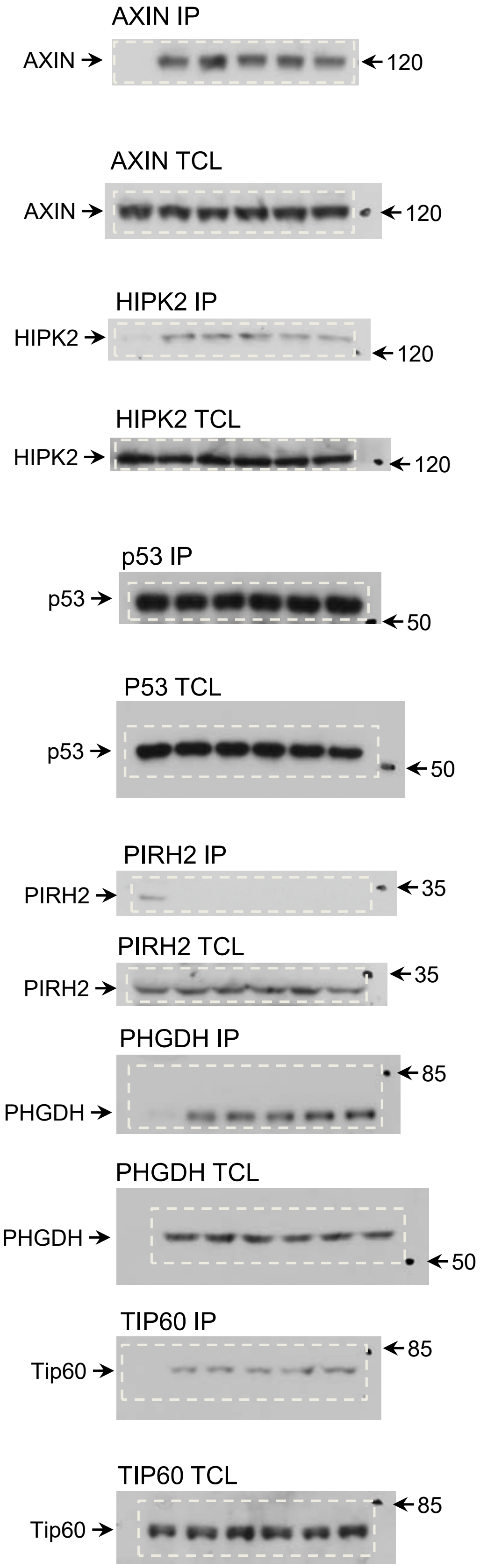
AXIN IP
AXIN
120
AXIN TCL
AXIN
120
HIPK2 IP
HIPK2
120
HIPK2 TCL
HIPK2
120
p53 IP
p53
50
P53 TCL
p53
50
PIRH2 IP
PIRH2
35
PIRH2 TCL
PIRH2
35
PHGDH IP
PHGDH
85
PHGDH TCL
PHGDH
50
TIP60 IP
Tip60
85
TIP60 TCL
Tip60
85

Fig. 3f

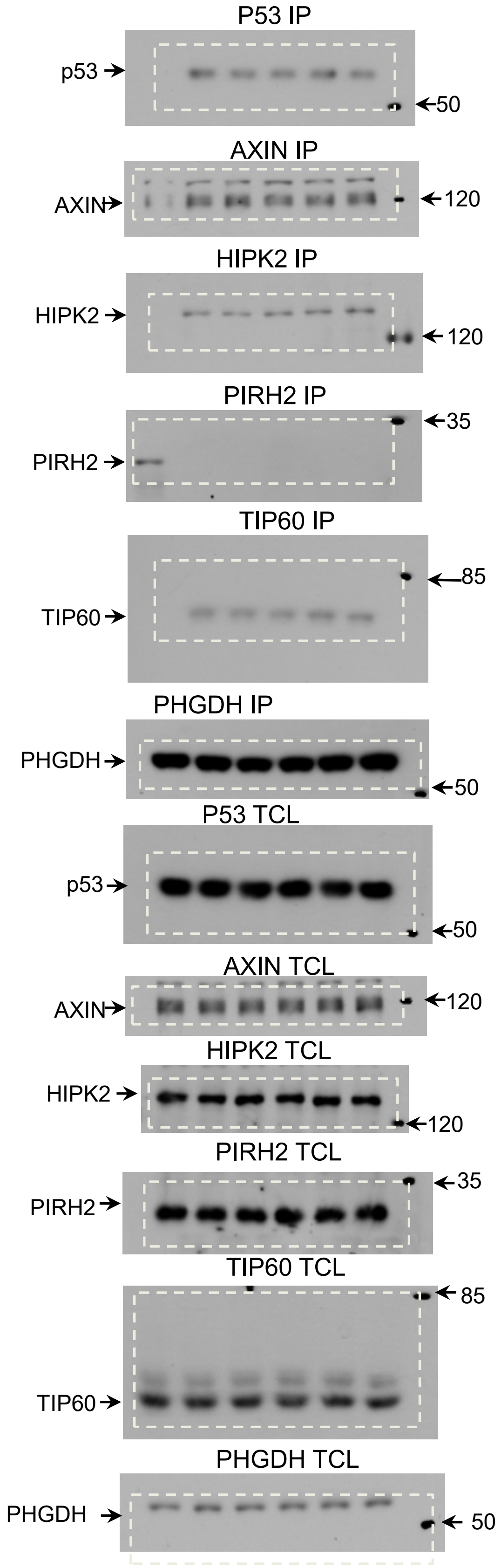

P53 IP
p53
50
AXIN IP
AXIN
120
HIPK2 IP
HIPK2
120
PIRH2 IP
PIRH2
35
TIP60 IP
TIP60
85
PHGDH IP
PHGDH
50
P53 TCL
p53
50
AXIN TCL
AXIN
120
HIPK2 TCL
HIPK2
120
PIRH2 TCL
PIRH2
35
TIP60 TCL
TIP60
85
PHGDH TCL
PHGDH
50

Fig. 3g

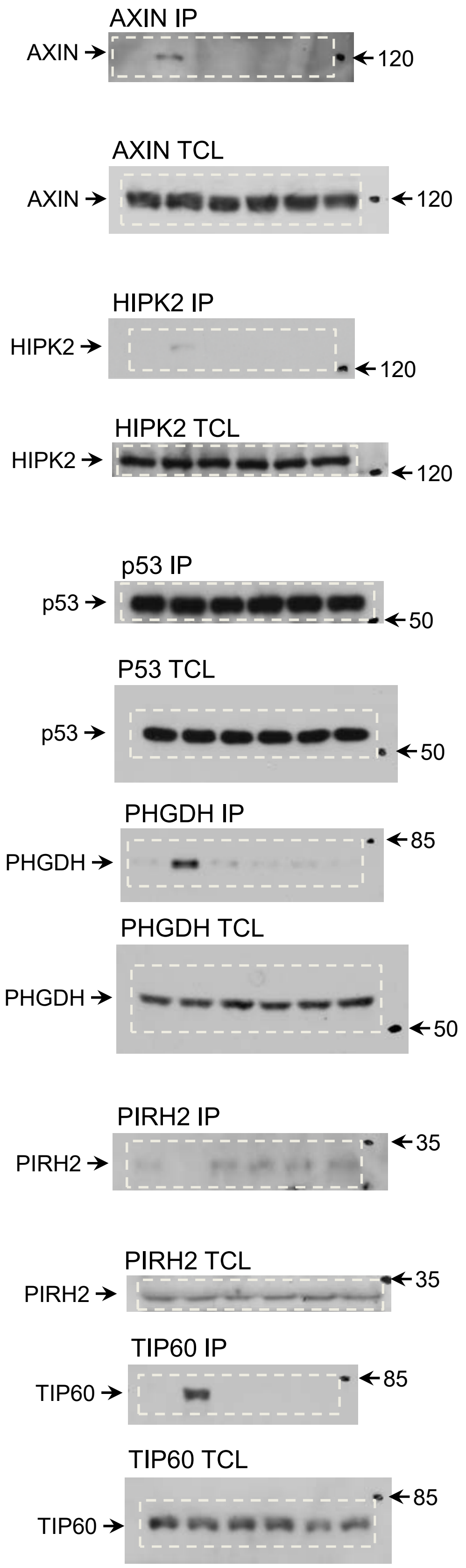

AXIN IP
AXIN
120
AXIN TCL
AXIN
120
HIPK2 IP
HIPK2
120
HIPK2 TCL
HIPK2
120
p53 IP
p53
50
P53 TCL
p53
50
PHGDH IP
PHGDH
85
PHGDH TCL
PHGDH
50
PIRH2 IP
PIRH2
35
PIRH2 TCL
PIRH2
35
TIP60 IP
TIP60
85
TIP60 TCL
TIP60
85

Fig. 3h

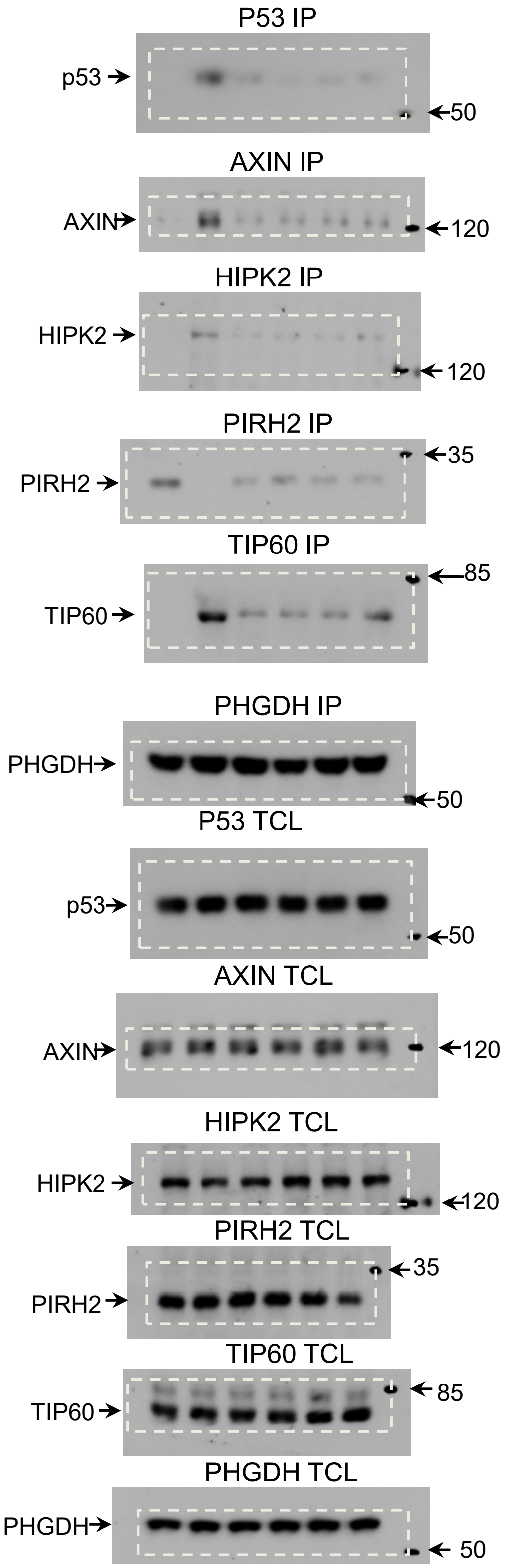

P53 IP
p53
50
AXIN IP
AXIN
120
HIPK2 IP
HIPK2
120
PIRH2 IP
PIRH2
35
TIP60 IP
TIP60
85
PHGDH IP
PHGDH
50
P53 TCL
p53
50
AXIN TCL
AXIN
120
HIPK2 TCL
HIPK2
120
PIRH2 TCL
PIRH2
35
TIP60 TCL
TIP60
85
PHGDH TCL
PHGDH
50

Fig. 3i

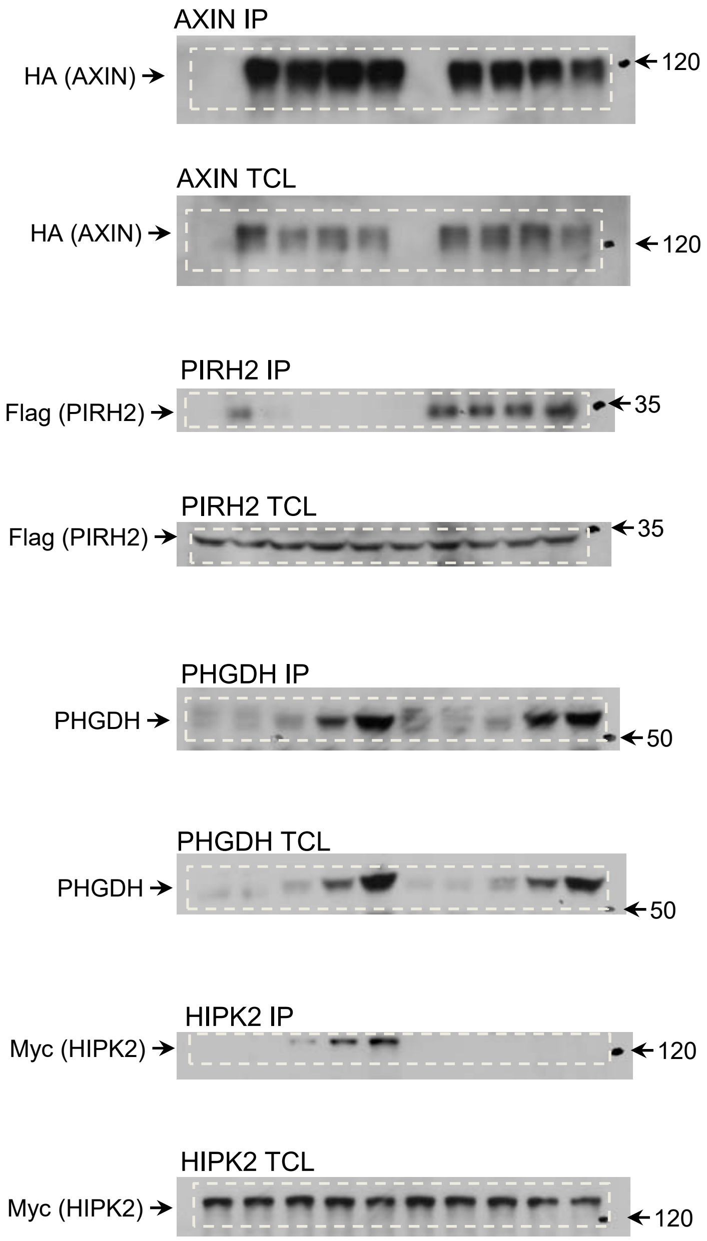

AXIN IP
HA (AXIN)
120
AXIN TCL
HA (AXIN)
120
PIRH2 IP
Flag (PIRH2)
35
PIRH2 TCL
Flag (PIRH2)
35
PHGDH IP
PHGDH
50
PHGDH TCL
PHGDH
50
HIPK2 IP
Myc (HIPK2)
120
HIPK2 TCL
Myc (HIPK2)
120

Fig. 3j

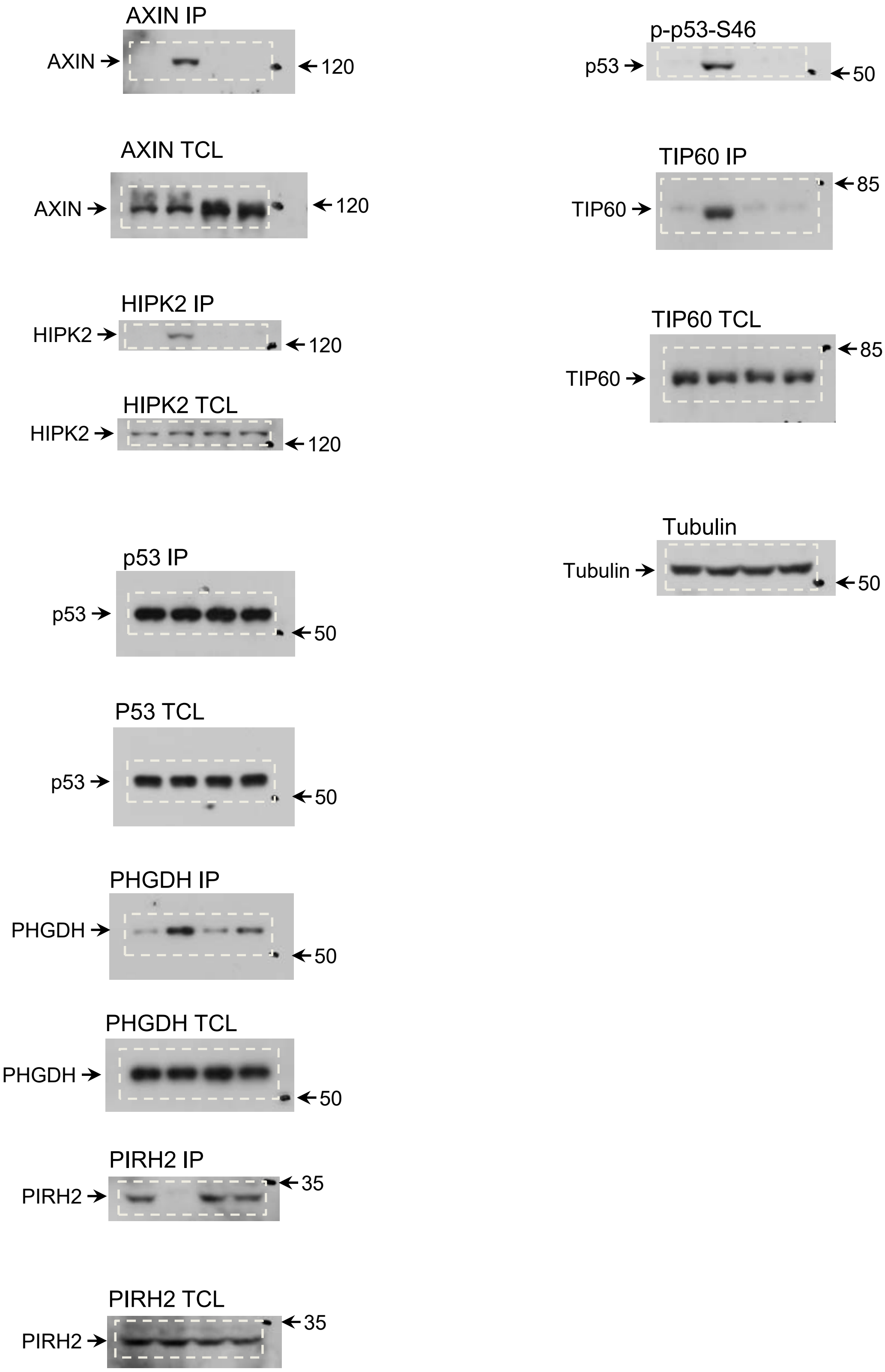
AXIN IP
AXIN
120
AXIN TCL
AXIN
120
HIPK2 IP
HIPK2
120
HIPK2 TCL
HIPK2
120
p53 IP
p53
50
P53 TCL
p53
50
PHGDH IP
PHGDH
50
PHGDH TCL
PHGDH
50
PIRH2 IP
PIRH2
35
PIRH2 TCL
PIRH2
35
p-p53-S46
p53
50
TIP60 IP
TIP60
85
TIP60 TCL
TIP60
85
Tubulin
Tubulin
50

Fig. 4b

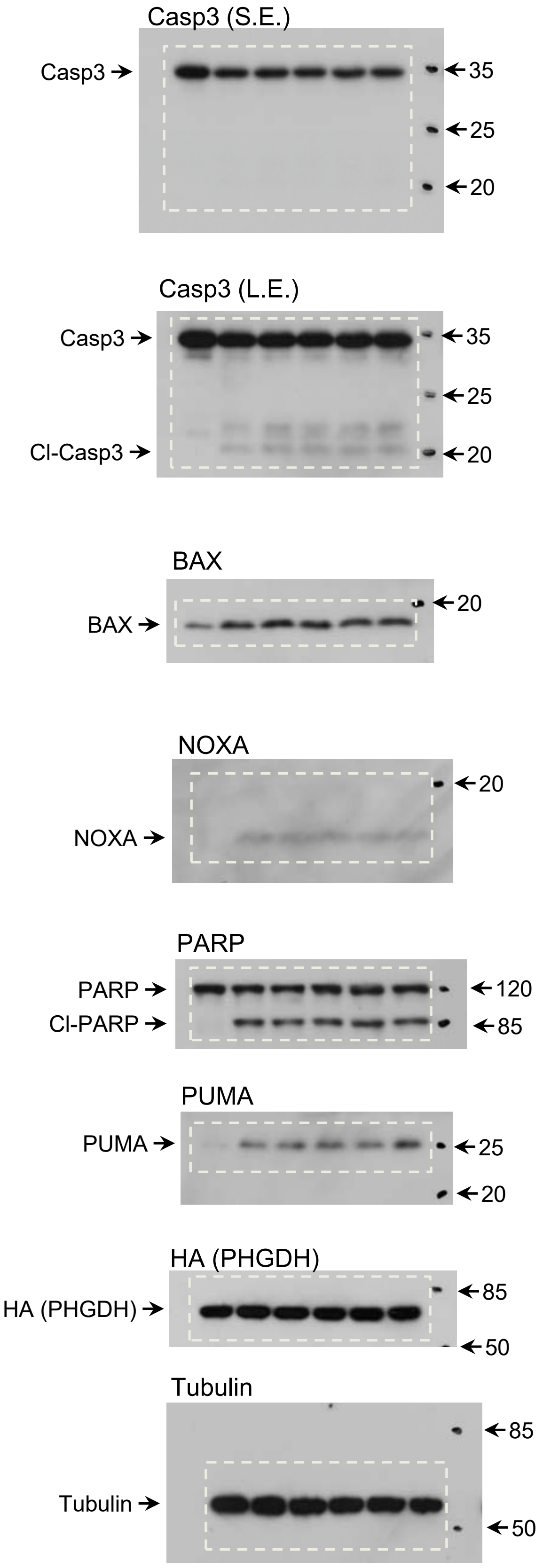
Casp3 (S.E.)
Casp3
35
25
20
Casp3 (L.E.)
Casp3
35
25
Cl-Casp3
20
BAX
BAX
20
NOXA
20
NOXA
PARP
PARP
120
Cl-PARP
85
PUMA
PUMA
25
20
HA (PHGDH)
85
HA (PHGDH)
50
Tubulin
85
Tubulin
50

Fig. 4c

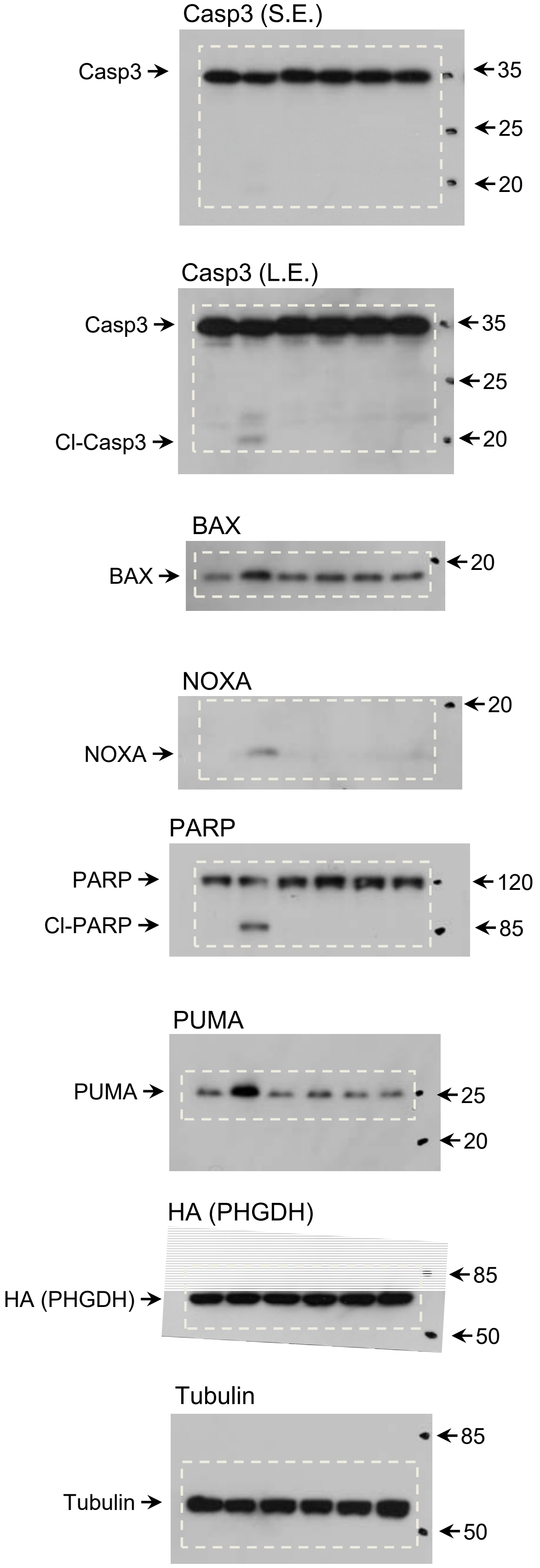
Casp3 (S.E.)
Casp3
35
25
20
Casp3 (L.E.)
Casp3
35
25
Cl-Casp3
20
BAX
BAX
20
NOXA
20
NOXA
PARP
PARP
120
Cl-PARP
85
PUMA
PUMA
25
20
HA (PHGDH)
85
HA (PHGDH)
50
Tubulin
85
Tubulin
50

Fig. 5c

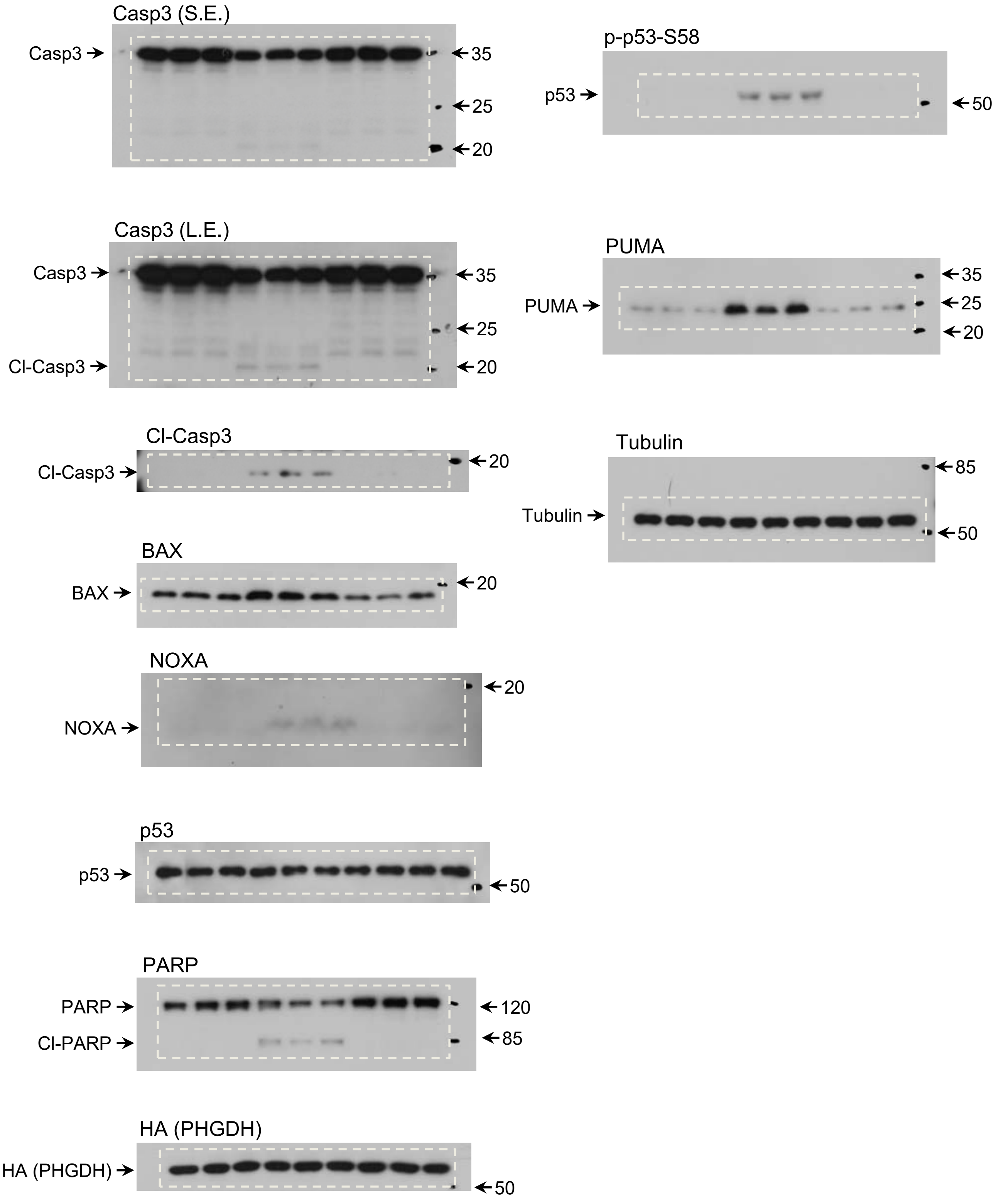
Casp3 (S.E.)
Casp3
35
25
20
Casp3 (L.E.)
Casp3
35
25
Cl-Casp3
20
Cl-Casp3
Cl-Casp3
20
BAX
BAX
20
NOXA
NOXA
20
p53
p53
50
PARP
PARP
120
Cl-PARP
85
HA (PHGDH)
HA (PHGDH)
50
p-p53-S58
p53
50
PUMA
PUMA
35
25
20
Tubulin
Tubulin
85
50

Supplementary information, Fig. S1a

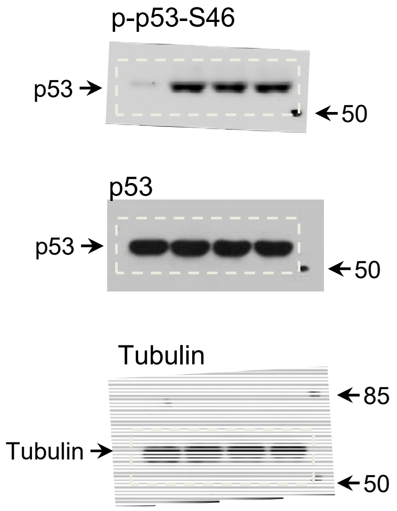

Supplementary information, Fig. S1b

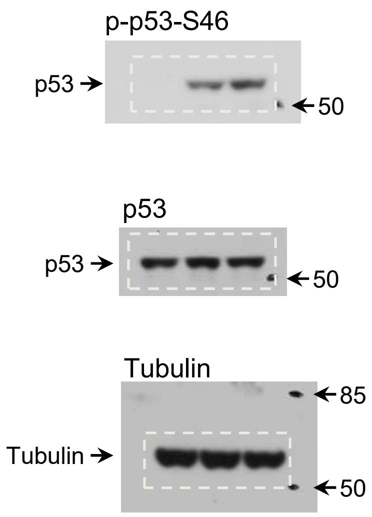

Supplementary information, Fig. S1c

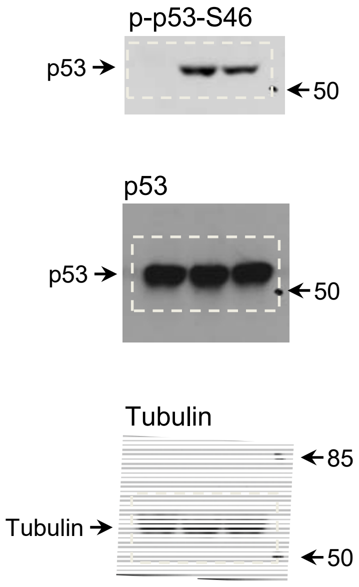

Supplementary information, Fig. S1d

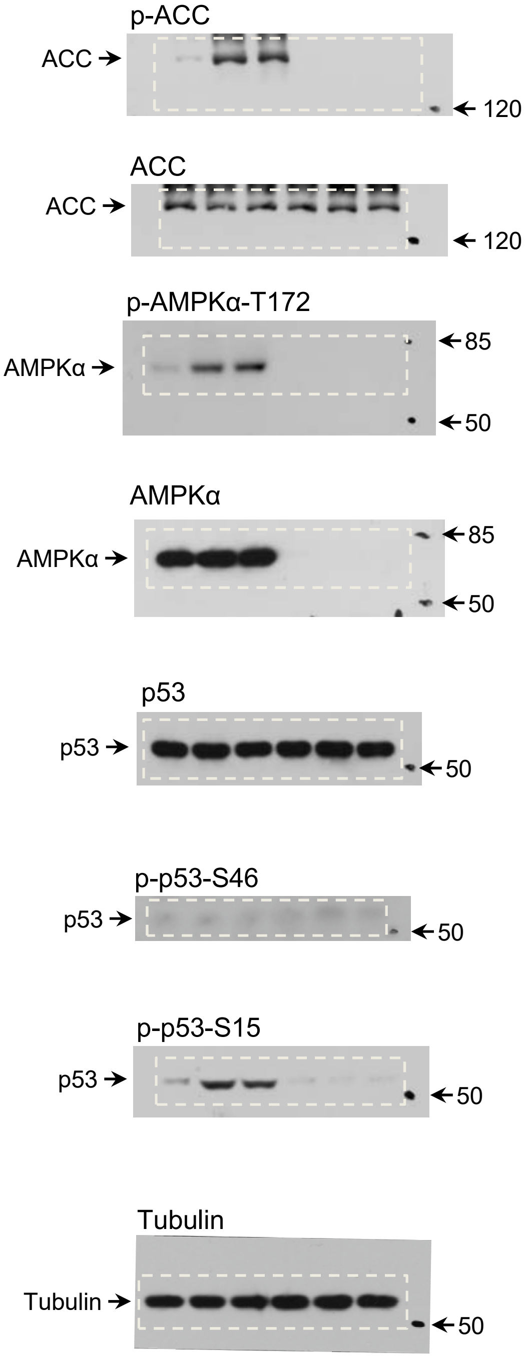

Supplementary information, Fig. S1e

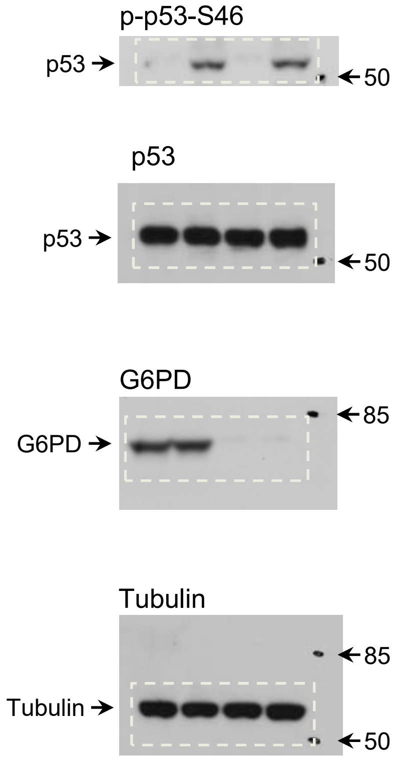

Supplementary information, Fig. S1f

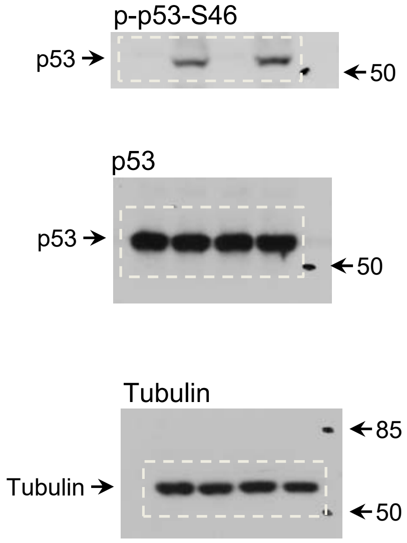

Supplementary information, Fig. S1h

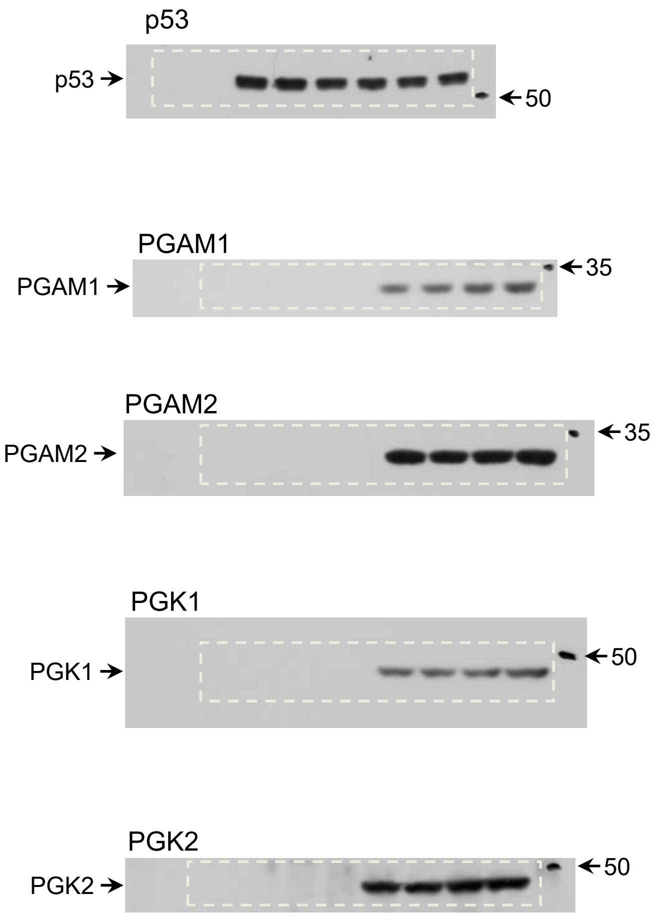

Supplementary information, Fig. S2b

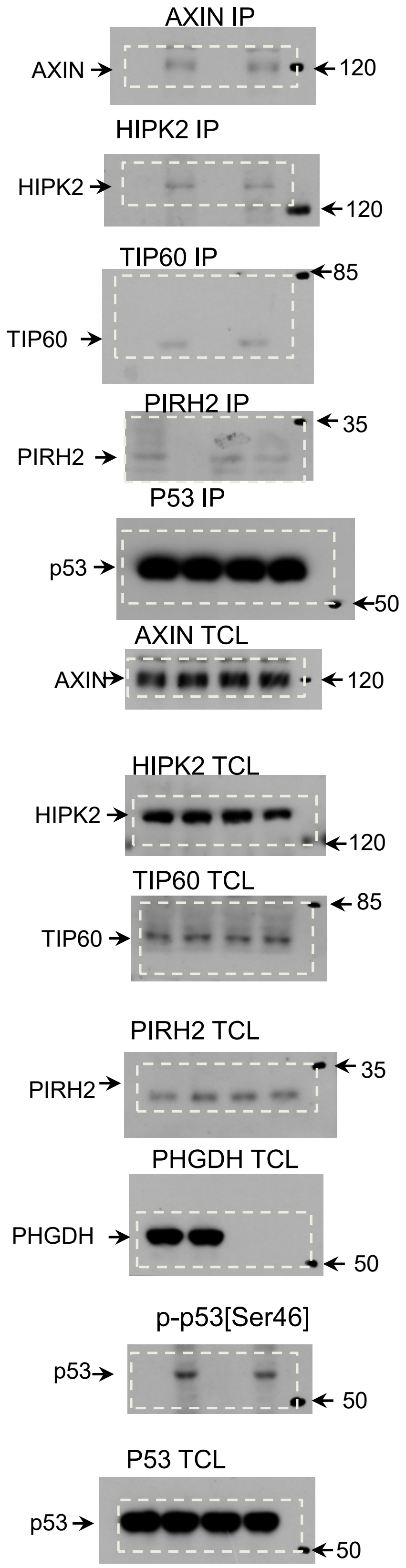

Supplementary information, Fig. S2c(upper)

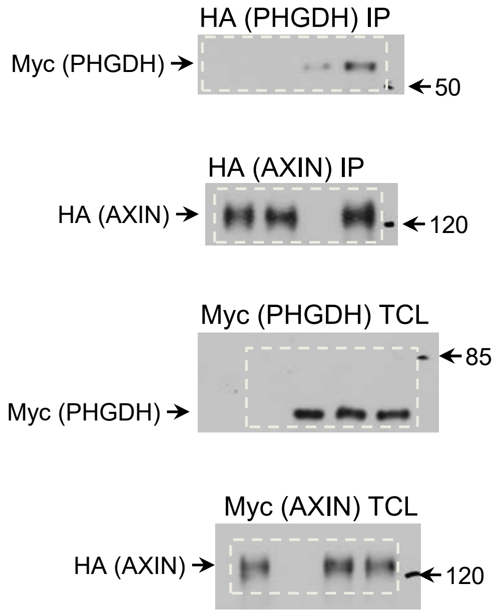

Supplementary information, Fig. S2c(lower)

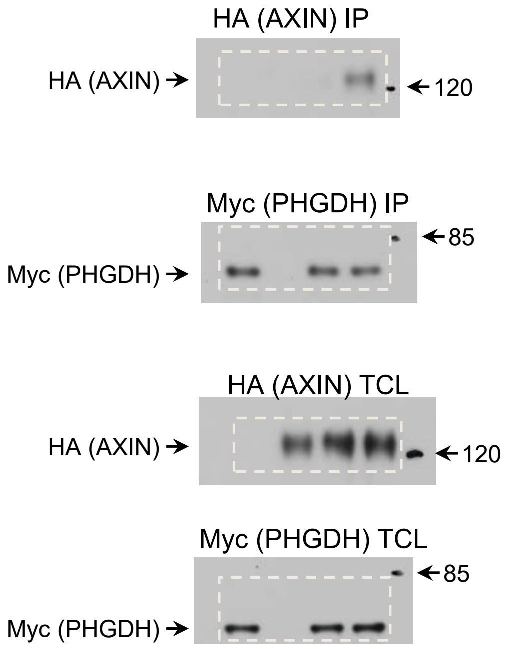

Supplementary information, Fig. S2d

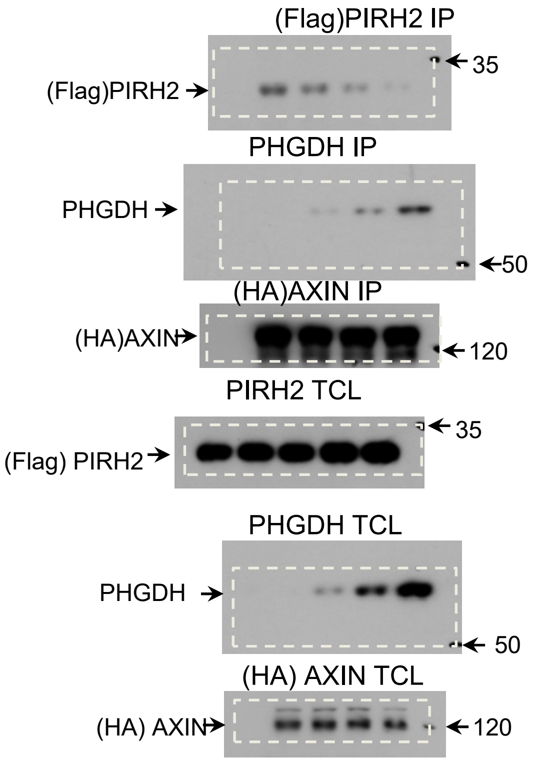

Supplementary information, Fig. S2e

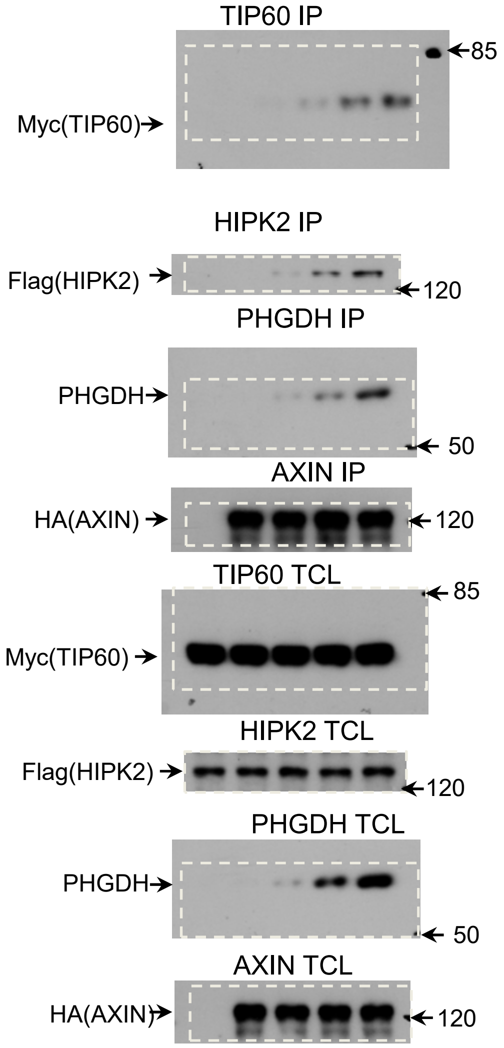

Supplementary information, Fig. S2f

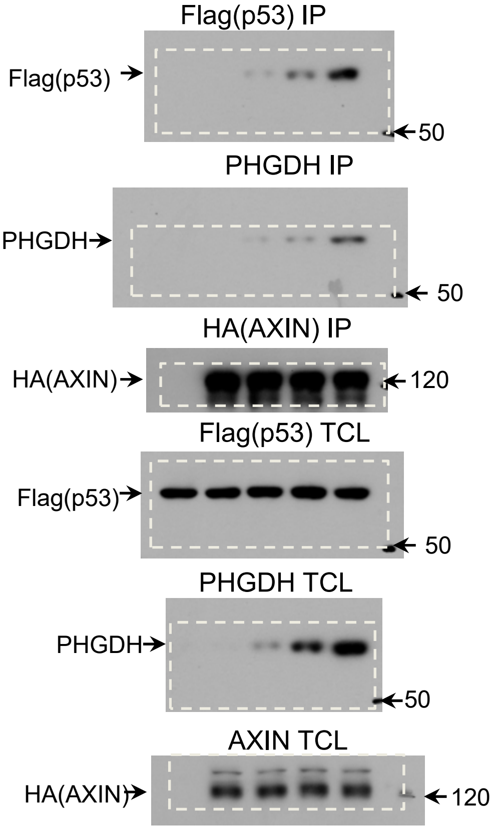

Supplementary information, Fig. S2g

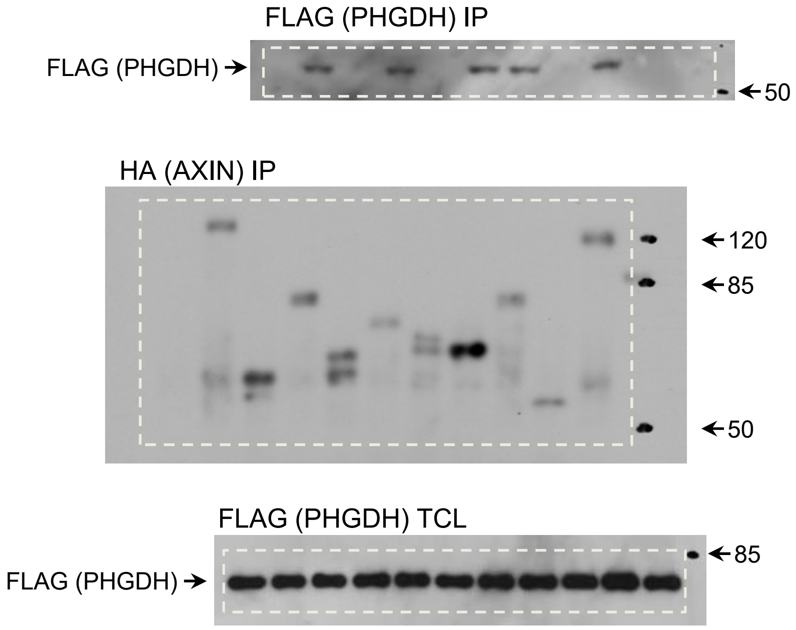

Supplementary information, Fig. S3e

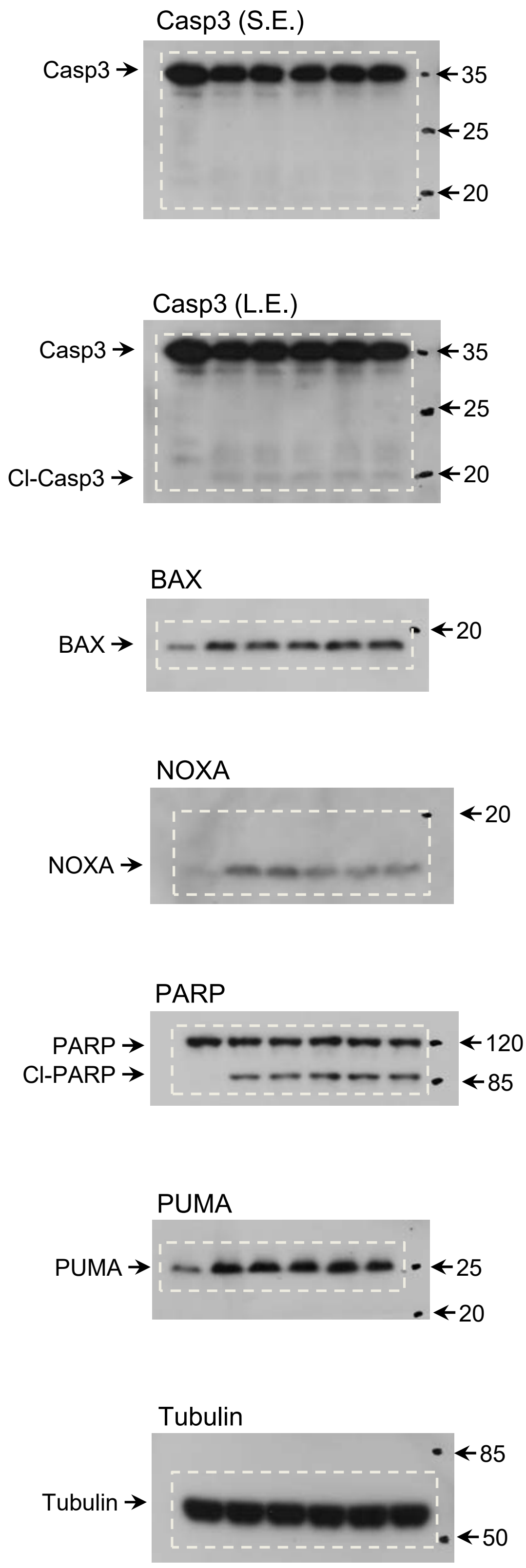

Supplementary information, Fig. S3f

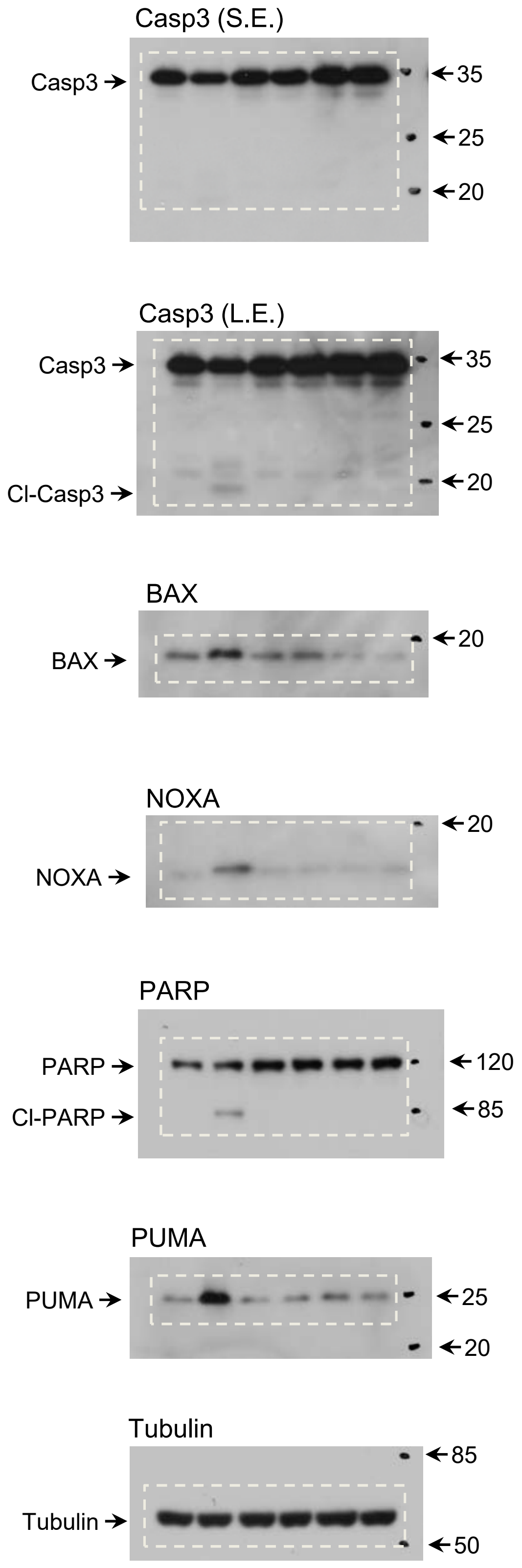

Supplementary information, Fig. S3g

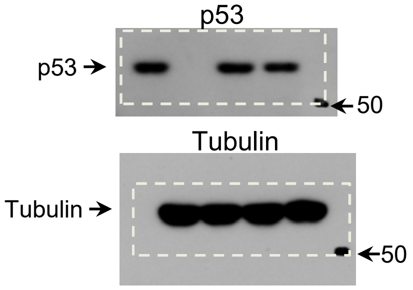

Supplementary information, Fig. S3j

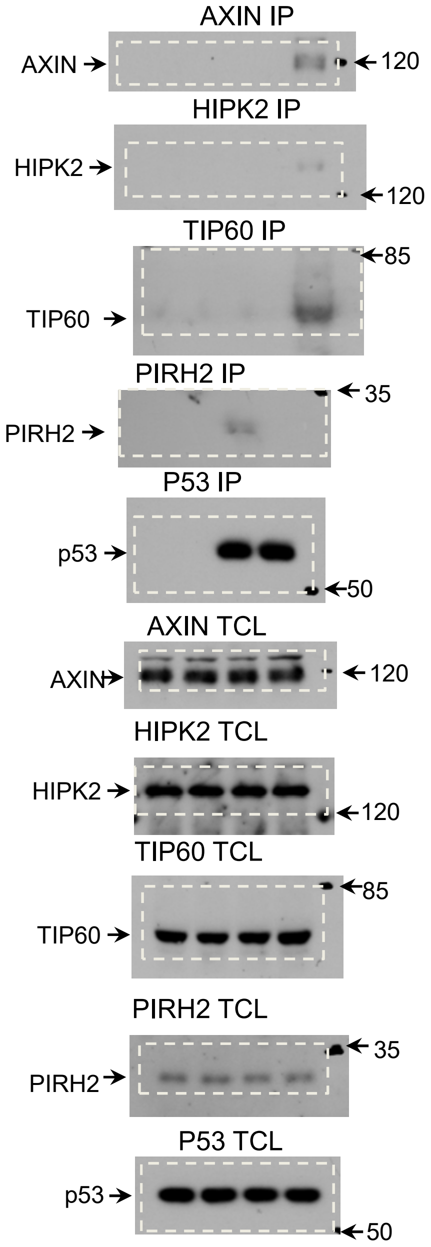

Supplementary information, Fig. S4b

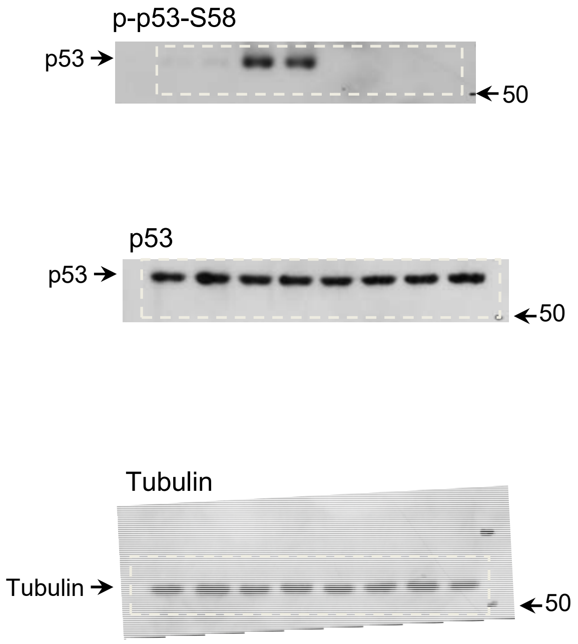

Supplement: Supplementary file 8 — Fig. S8 [file 41422_2023_874_MOESM8_ESM.pdf]
